# Antiretroviral therapy in the peripartum period impairs post pregnancy cardiac reverse remodeling in a rodent model

**DOI:** 10.1038/s41467-026-75510-x

**Published:** 2026-07-17

**Authors:** Nicole Taube, Mateus P. Mori, Sherry Coulter, Madeline Jeshurin, Julia House, Cristina A. Nadalutti, Don A. Delker, Michelle C. Cora, Charan K. Ganta, David Cunefare, Helen Cunny, Suramya Waidyanatha, Georgia K. Roberts, Vicki Sutherland, Dawn M. Fallacara, Sophia A. Cochran, Elena J. Ciesielska, Grzegorz L. Ciesielski, Janine H. Santos

**Affiliations:** 1 Mechanistic Toxicology Branch, Division of Translational Toxicology, National Institute of Environmental Health Sciences (NIEHS), National Institutes of Health (NIH), 111 TW Alexander Dr, Research Triangle Park, NC USA; 2 Integrative Bioinformatics Support Group, Division of Intramural Research, National Institute of Environmental Health Sciences (NIEHS), National Institutes of Health (NIH), 111 TW Alexander Dr, Research Triangle Park, NC USA; 3 Comparative And Molecular Pathogenesis Branch, Division of Translational Toxicology, National Institute of Environmental Health Sciences (NIEHS), National Institutes of Health (NIH), 111 TW Alexander Dr, Research Triangle Park, NC USA; 4 Office of Program Operations, Division of Translational Toxicology, National Institute of Environmental Health Sciences (NIEHS), National Institutes of Health (NIH), 111 TW Alexander Dr, Research Triangle Park, NC USA; 5 Systems Toxicology Branch, Division of Translational Toxicology, National Institute of Environmental Health Sciences (NIEHS), National Institutes of Health (NIH), 111 TW Alexander Dr, Research Triangle Park, NC USA; 6 Battelle Memorial Institute, Research Group for Life Sciences, 505 King Avenue, Columbus, OH USA; 7 Department of Biology, University of North Florida, 1 UNF Dr., Jacksonville, FL USA; 8 Present Address: Global Toxicology & Safety Pharmacology J&J Innovative Medicine, Johnson & Johnson, 1000 U.S. Route 202 South, Raritan, NJ USA

**Keywords:** Energy metabolism, Heart failure

## Abstract

Cardiovascular (CV) disease is the leading cause of maternal mortality worldwide and has been partially linked to the cardiometabolic remodeling required to support fetal growth. In healthy pregnancies, these adaptations reverse postpartum without long-term consequences; however, impaired reverse remodeling (RR) increases future CV risk. Pregnant women living with HIV face additional risk, though it remains unclear whether this is driven by chronic low-level viremia or continuous antiretroviral therapy (ART). ART used during pregnancy often includes nucleoside reverse transcriptase inhibitors (NRTIs), which can affect mitochondria - organelles essential for maintaining cardiac function. Here, we tested the hypothesis that NRTI-containing ART negatively impacts the maternal heart. Using a rat model, we show that ART impairs pregnancy-associated cardiac RR. Mechanistically, ART induced mitochondrial alterations in both the heart and the liver, leading to dyslipidemia that collectively compromised cardiac function. These findings emphasize the importance of maternal cardiovascular health research and the need for further studies on the long-term effects of ART.

## Abstract (web summary)

In rats, HIV medications used in pregnancy alter the heart’s recovery after birth. The drugs alter energy-making parts of cells in the heart and liver and disrupt blood fats, suggesting a vulnerable maternal window.

## Introduction

Pregnancy is a dynamic process associated with significant cardiometabolic changes. During gestation, the increased hemodynamic load leads to cardiac hypertrophy that is accompanied by increased cardiac output and decreased systemic vascular resistance^1^. Metabolically, the mother switches carbon sources to include more lipids or ketone bodies favoring glucose uptake by the growing fetus^2^. Unlike in pathological hypertrophy, these physiological changes reverse after pregnancy – a process termed reverse remodeling (RR) that typically initiates a few weeks after birth, pending lactation, and is largely completed within 6 months post-delivery^3^. Maternal inability to adapt to these changes can expose underlying or previously silent conditions, including cardiac pathology that may persist postpartum. Partially owing to these, cardiovascular disease (CVD) in pregnancy is the leading cause of maternal mortality, including in North America^4^. While this may be due to other factors, such as access to consistent healthcare or pre-existing cardiovascular conditions, cardiometabolic remodeling is an important component of maternal health^5–7^. The mechanisms underlying these profound changes in the maternal cardiometabolic system, including the signals that drive RR, remain elusive. This is partially due to the lack of funding and support for studies during pregnancy.

CVD in pregnancy is likely to be further compounded by environmental factors and comorbidities, such as chronic infections. In this context, CVD has also become a leading cause of morbidity and mortality in pregnant women living with HIV. These individuals face additional physiological challenges due to chronic viremia and the continued exposure to antiretrovirals (ART)^8^. While the effects of ART on cardiovascular health during pregnancy remain poorly understood, studies in the general population of people living with HIV have identified metabolic alterations, including changes in lipid profiles, as key contributors to long-term cardiometabolic risk^9^. Importantly, people living with HIV have an elevated risk of composite CV complications, including stroke, peripheral vascular disease, ischemia, myocardial infarction and heart failure, with disproportionately higher risk observed in individuals under 40 years of age^10^. However, a critical knowledge gap persists in disentangling the relative contributions of HIV infection itself versus ART exposure to this increased risk. Furthermore, the extent to which these factors influence CVD risk in pregnant women living with HIV remains unknown.

Estimates indicate that 50% of the ~40 million patients living with HIV worldwide are women^11,12^^,^. About 1.2 million women living with HIV give birth globally per year, and in the US alone the annual birth rate of pregnant women living with HIV is estimated to be around 7,000^13^. HIV is currently a chronic condition and patients living with HIV who can maintain durable viral suppression through consistent ART use have a normal or near-normal life expectancy. ART has revolutionized healthcare in the management of HIV/AIDS by significantly reducing HIV transmission, preserving immune function, decreasing HIV-related morbidity and mortality, and overall improving the quality of life of people living with HIV. Nevertheless, it remains imperative to understand the cumulative effects of chronic ART exposure, particularly during the perinatal and peripartum periods, given the necessity for lifelong therapy. Notably, nucleoside reverse transcriptase inhibitors (NRTIs), which are known to negatively impact mitochondrial function by affecting mitochondrial DNA (mtDNA)^14,15^ remain a staple of HIV therapy, including during pregnancy where current treatment guidelines indicate the use of combination therapy with at least two NRTIs^16^. Given mitochondria are responsible for 90% of the ATP produced in cardiomyocytes and occupy 40% of their volume, one would predict adverse CV outcomes in pregnant patients taking NRTI-containing ART. Nevertheless, studies on pregnant women living with HIV or animal models have primarily focused on the effects of HIV/AIDS and its management in the offspring^14^, leaving an informational void about the off-target effects of the drugs on maternal health. By continuously exposing adult female rats from gestational day 6 (GD6) to postpartum day 120 (PPD120) to the NRTIs abacavir (ABC) and lamivudine (3TC) in combination with the integrase inhibitor dolutegravir (DTG), we characterized the effects of these drugs on cardiometabolic changes associated with pregnancy and RR. Our data revealed effects in the heart and liver that contribute to blunted RR in the postpartum period, collectively highlighting the need for further studies on maternal health, and the potential effects of NRTI-containing therapies in this vulnerable period of life.

## Results

### cART during the peripartum period does not affect pregnancy outcomes

**Fig. 1:**
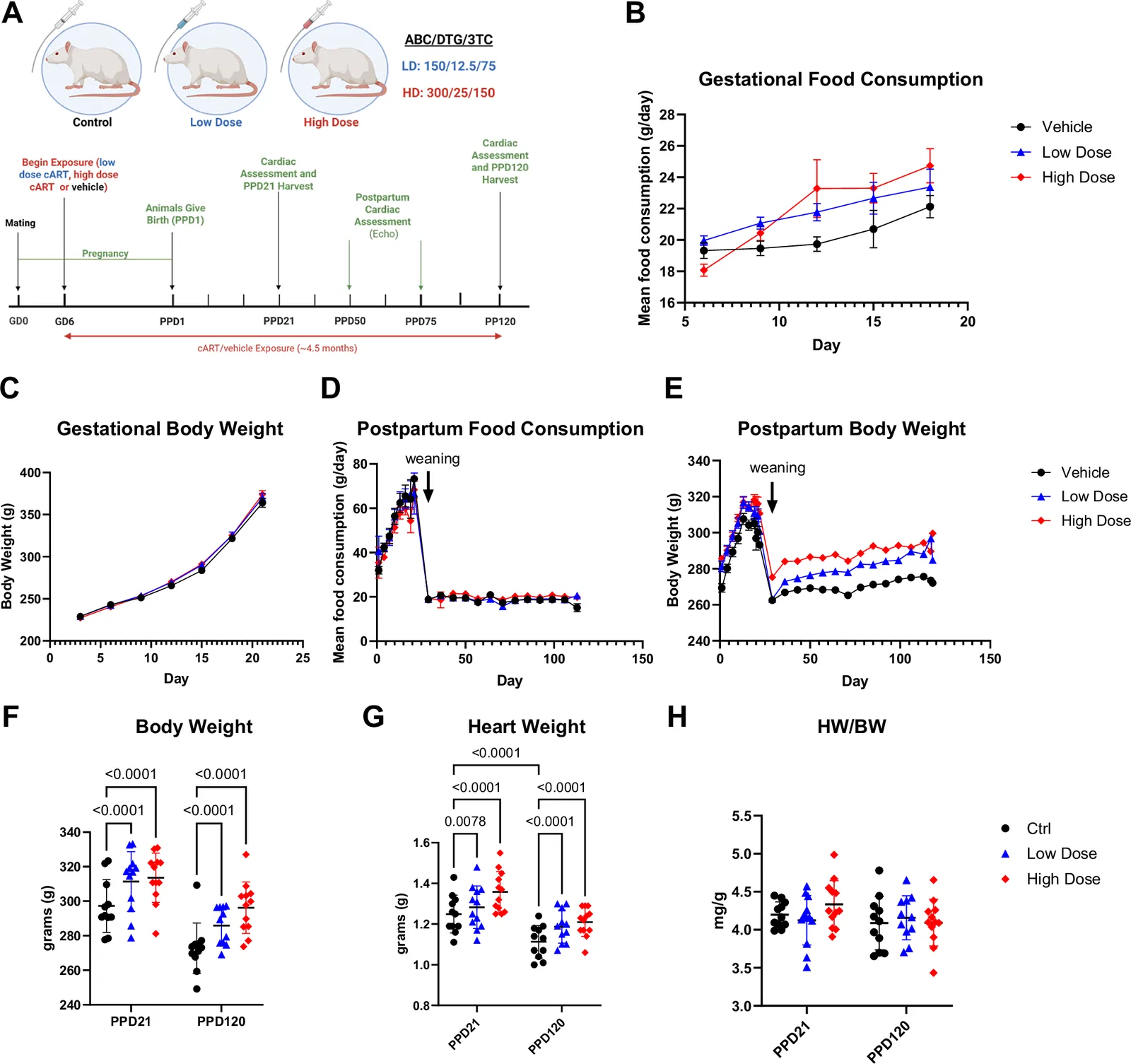
Peripartum ABC/DTG/3TC exposure increases body weight and heart weight in Sprague-Dawley female rats. **A** Diagram of animal exposure to combination antiretroviral therapy (cART) composed of abacavir (ABC), dolutegravir (DTG), and lamivudine (3TC) and timeline of exposure through gestational day (GD) 6 until postpartum day (PPD) 120. Created in BioRender. Taube, N. (https://BioRender.com/yobzefl. Gestational food consumption (**B**) and body weight (**C**) over time (*n* = 22 animals/group). Postpartum food consumption (**D**) and body weight (**E**) over time (*n *= 22 animals/group). Body weight (**F**), heart weight (**G**) and heart weight normalized to body weight (**H**) at PPD21 and PPD120 (*n* = 12 animals/group/timepoint). Data were analyzed via two-way ANOVA, data shown as mean ± SEM.

A diagram of the experimental design is presented in Fig. 1A. Briefly, dams were continuously exposed to either vehicle control of aqueous 0.2% methylcellulose/0.1% Tween 80 (Veh) or one of two doses of ABC/DTG/3TC, which from here on we refer to as combination ART (cART). Exposures occurred from gestational day 6 (GD6) to postpartum day 120 (PPD120). This timeline modeled early embryonic exposure while ensuring consistent confirmation of pregnancy in the rodent model and allowed exposure to continue through the postpartum remodeling period. Until June 2025, this cART regimen represented the preferred guideline-based therapy for pregnant women living with HIV. Since then, updated guidelines have shifted toward an alternative regimen that also includes NRTIs, although ABC and 3TC are no longer part of the preferred combination. Nevertheless, each component of the regimen evaluated in this study remains in use as either preferred or alternative therapy for select peripartum patients, depending on factors such as prior ART exposure, pregnancy-related complications, the presence of chronic, acute or recent HIV infection, as well as their use in prophylactic settings^17^. The doses utilized in this study were selected based on well-established interspecies differences, as well as data from prior toxicokinetic and dose-range–finding studies conducted to characterize drug plasma concentrations and assess potential toxicity (Beames et al., in preparation). In rats, ARTs typically exhibit faster clearance, lower bioavailability, and reduced tissue exposure compared with humans^18,19^, necessitating higher mg/kg dosing to achieve systemic and tissue drug levels comparable to those observed clinically. The dosing regimens used in this study preserved the relative ratio of NRTIs to the integrase inhibitor as administered in patients living with HIV and were given as 150/12.5/75 mg/kg or 300/25/150 mg/kg of ABC/DTG/3TC, respectively. These doses correspond to ~2.5× and 5× the human-equivalent dose for a 60 kg patient and are referred to, throughout the manuscript, as low dose (LD) and high dose (HD), respectively.

Cardiac assessments were performed, followed by tissue harvest, at postpartum day 21 (PPD21) and postpartum day 120 (PPD120) (Fig. 1A). These points were selected to represent the end of lactation (PPD21) and three months thereafter (PPD120), thereby encompassing the period of postpartum reverse cardiac remodeling. No HIV virus or viral proteins were present under our experimental conditions, allowing us to dissect out the effects of the drugs themselves. Pregnancy outcomes were generally similar between the groups, including reproductive performance, fertility and fecundity, gestation length, litter size and numbers as well as clinical observations (Supplementary Fig. 1A-C). There were no significant changes in gross necropsy findings, including in the heart, kidneys, liver, lungs and mesenteric rolls (Supplementary Fig. 1B). All in vivo data associated with this study is available as source data. No changes in food consumption or body weight were observed during pregnancy (Fig. 1B, C). While food intake was not changed in the postpartum period (Fig. 1D), cART-treated animals gained significantly more weight over time, which started at weaning (PPD21, Fig. 1E, F). Heart size also increased by treatment (Fig. 1G), but it was not different than controls if evaluated relative to increased body weight (Fig. 1H). Thus, cART treatment showed no signs of overt toxicity when administered during the time encompassing pregnancy, lactation and the 3 months that followed. This is consistent with previous studies, although clinical safety data on pregnant women living with HIV is sparse particularly when using the combined therapy.

### Differential effects of cART on cardiac function during the peripartum period

**Fig. 2:**
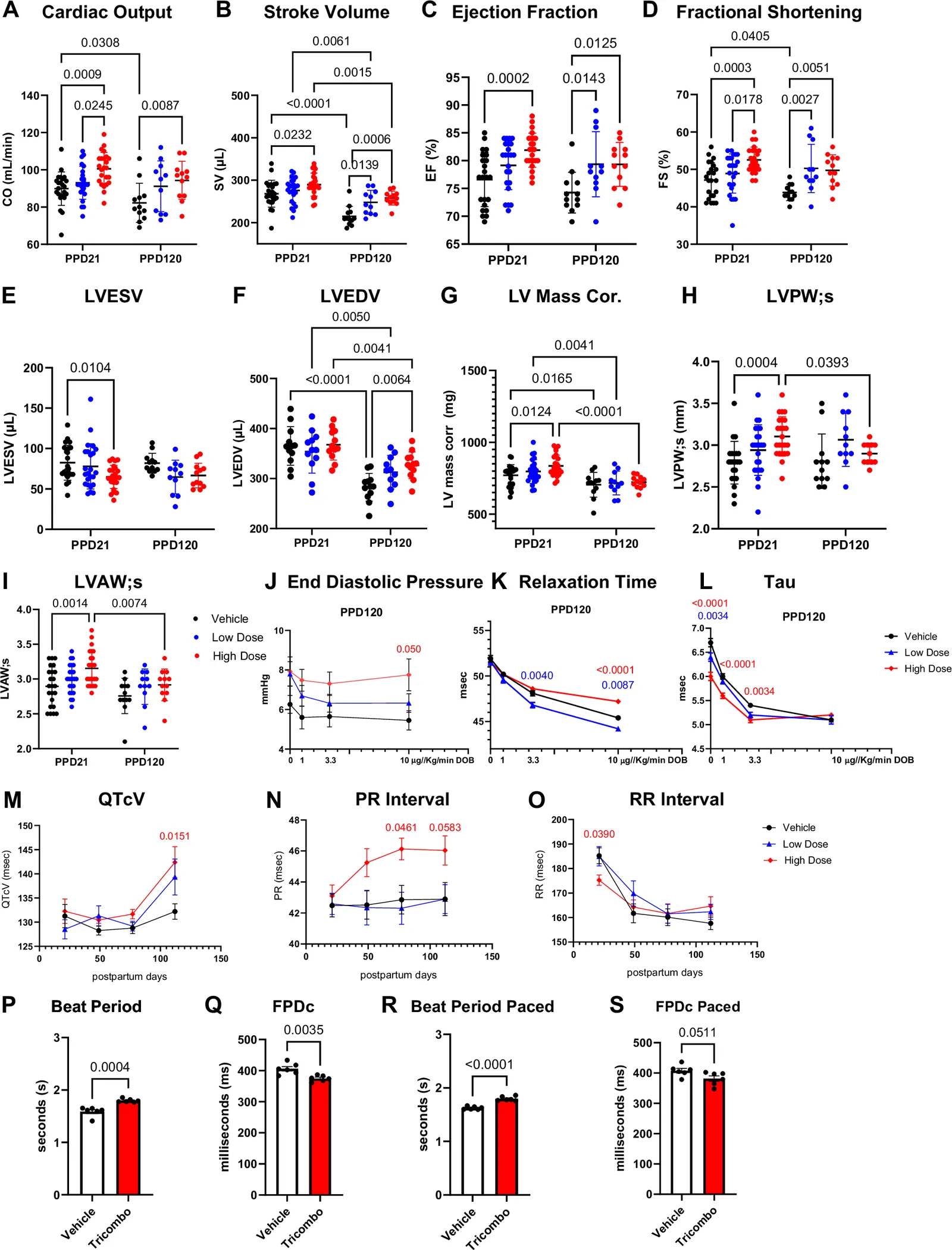
ABC/DTG/3TC administration alters contractile and electrophysiological function postpartum. Echocardiography measurements of vehicle, LD and HD ABC/DTG/3TC-treated dams at PPD21 and PPD120 for (**A**) cardiac output (CO), **B** stroke volume (SV), **C** ejection fraction (EF), **D** fractional shortening (FS), **E** left ventricular end systolic volume (LVESV), **F** left ventricular end diastolic volume (LVEDV), **G** corrected left ventricular mass, **H** left ventricular posterior wall in systole (LVPW;s), **I** left ventricular anterior wall in systole (LVAW;s). Data were analyzed via two-way ANOVA with Tukey’s multiple comparisons (*n* = 24 rats/group (PPD21) or 12 rats/group (PPD120)). **J** End diastolic pressure at PPD120, relaxation time (**K**), and tau (**L**) measured under dobutamine challenge. Data were analyzed via two-way ANOVA with Tukey’s multiple comparisons (*n* = 12 rats/group/timepoint), *p* values shown as group vs. vehicle. Electrocardiogram measurements of vehicle, low and high dose ABC/DTG/3TC dams at PPD21 and PPD120 for (**M**) QTcV (**N**), PR interval and (**O**) RR interval. Results were analyzed via two-way ANOVA with Tukey’s multiple comparisons (*n *= 12 rats/group/timepoint). Multielectrode array measurements for human iPSC cardiomyocytes dosed for 7 days with ABC/DTG/3TC including (**P**) beat period, (**Q**), field potential duration corrected for beat period (FPDc), **R** paced beat period, and (**S**) paced field potential duration corrected for beat period. Data were analyzed via one-way ANOVA, data shown as mean ± SEM (*n* = 6 wells/group).

We next characterized the effects of cART on CV function by echocardiography (ECHO). We used data from PPD21 to capture pregnancy-induced changes, as these begin early in pregnancy and remain through lactation. Data from PPD120 were used to characterize post-pregnancy physiology, including RR. Increased cardiac output (CO), stroke volume (SV), ejection fraction (EF) and fractional shortening (FS) are expected changes associated with pregnancy, which were significantly increased by cART at the HD at PPD21 relative to the controls (Fig. 2A–D). Heart rate (HR) was not altered under baseline measurements (Supplementary Fig. 2A), indicating that increased CO was driven by changes in SV. Animals on the HD showed changes in structural measures at PPD21 that were beyond vehicle control, including increased left ventricular (LV) mass, posterior and anterior wall thickness, and decreased systolic volume, which are all consistent with physiological hypertrophy observed in pregnancy (Fig. 2E–I). Surprisingly, however, these parameters did not fully return to pre-partum physiology at PPD120, including in the LD group, with significant differences relative to the vehicle-controls still identified for CO, SV, EF, FS (Fig. 2A–D, F). These data indicate that, at 4 months postpartum, hearts of treated animals continued to exhibit enhanced function despite the absence of pregnancy - a period during which RR would normally be expected to occur. Notably, indication of RR was found in the control animals as early as 4 weeks post-weaning, but it was significantly blunted in the treated counterparts (Supplementary Fig. 2A). The sustained cardiac workload may reflect the increased body size of the treated animals, which by PPD120 weighed essentially the same as control animals at PPD21 (Fig. 1E, F). Alternatively, but not mutually exclusive, they may reflect failure to initiate or complete RR.

Rats were then submitted to a dobutamine challenge. Dobutamine is a β-adrenergic agonist that stimulates contractility and HR, informing on how the heart responds to sympathetic nervous system stimulation. Several significant differences were observed between the treated and vehicle-controls groups, with increased severity found in HD-treated animals (Supplementary Fig. 2B). Notably, we found increased end diastolic pressure and increased relaxation time at PPD120 (Fig. 2J, K)–features often associated with diastolic dysfunction^20^, including in people living with HIV^10^. While a decrease in tau was initially observed in this group relative to controls, increasing dobutamine concentrations mitigated this difference (Fig. 2L), suggesting a reduced stress response, or decreased contractile reserve, as seen in diastolic dysfunction^21^. Lastly, we found changes in baseline measurements of contractility, including a reduction in contraction time at both time points and increased contraction index (Supplementary Fig. 2B). The milder effects on the LD-treated animals indicate dose-dependency in the severity of the phenotypes, although non-monotonic responses were observed in some parameters (e.g., relaxation time). This is consistent with well-documented toxicological literature including on the effects of DTG in the heart^22,23^. No significant difference in blood pressure was observed between the groups (Supplementary Fig. 2C).

Electrocardiograms (ECGs) were also performed to define any effects on electrical signaling in the heart, which revealed increases in QT (at PPD120) and PR intervals, and a decrease in the RR interval at PPD21 that was significant in the HD-treated animals (Fig. 2M–O). No changes in QRS were observed (Supplementary Fig. 2D). An increase in QT indicates longer time between depolarization and repolarization of the left ventricle whereas an increase in PR reflects delayed electrical signals from the sinoatrial node to the left ventricle. While these indicate that cART may increase the risk of arrhythmias or atrial fibrillation, measuring and interpreting ECG data from rodents is challenging because it can often be influenced by anesthetics and, given intrinsic differences to humans in ion channel expression, be of questionable translatability. To gain more insights into this, we treated induced pluripotent stem cells (iPSC)-derived human cardiomyocytes in culture with the drugs, analyzing different ECG-relevant parameters using an impedance-based multielectrode array (MEA). Cells were exposed to doses equivalent to half (0.5X) of their concentration in the plasma, which is closer to the amounts in the heart (see below). Control experiments demonstrated that cells created a beating syncytium and were viable when kept in culture for up to 10 days. As beating patterns decreased and signs of drug toxicity increased by day 10, experiments herein were done in 7-day cardiomyocyte cultures.

About 60,000 cells per well were exposed to the drugs for 7 consecutive days, when field potential duration (FPD), which reflects the timing between depolarization and repolarization of cells within each well, was measured in non-paced or paced cells. Pacing was used to synchronize the population so that any effects identified would be unrelated to spontaneous beating, and FPD was corrected to account for beat period, which is known to correlate to FPD. No differences between non-treated and vehicle-exposed cells were identified (Supplementary Fig. 2D). We found that treated cells had increased beat period and decreased FPD whether the cells were or not paced (Fig. 2P–S), reproducing the ECG changes observed in the rats, including those associated with QT prolongation. These collective findings suggest that cART exposure in the peripartum period leads to subclinical features of diastolic dysfunction, including altered electrophysiological properties and hypertrophy, that accompanies the blunted cardiac RR identified 4 months postpartum.

### cART alters cardiac mitochondrial DNA content, integrity and structure

**Fig. 3:**
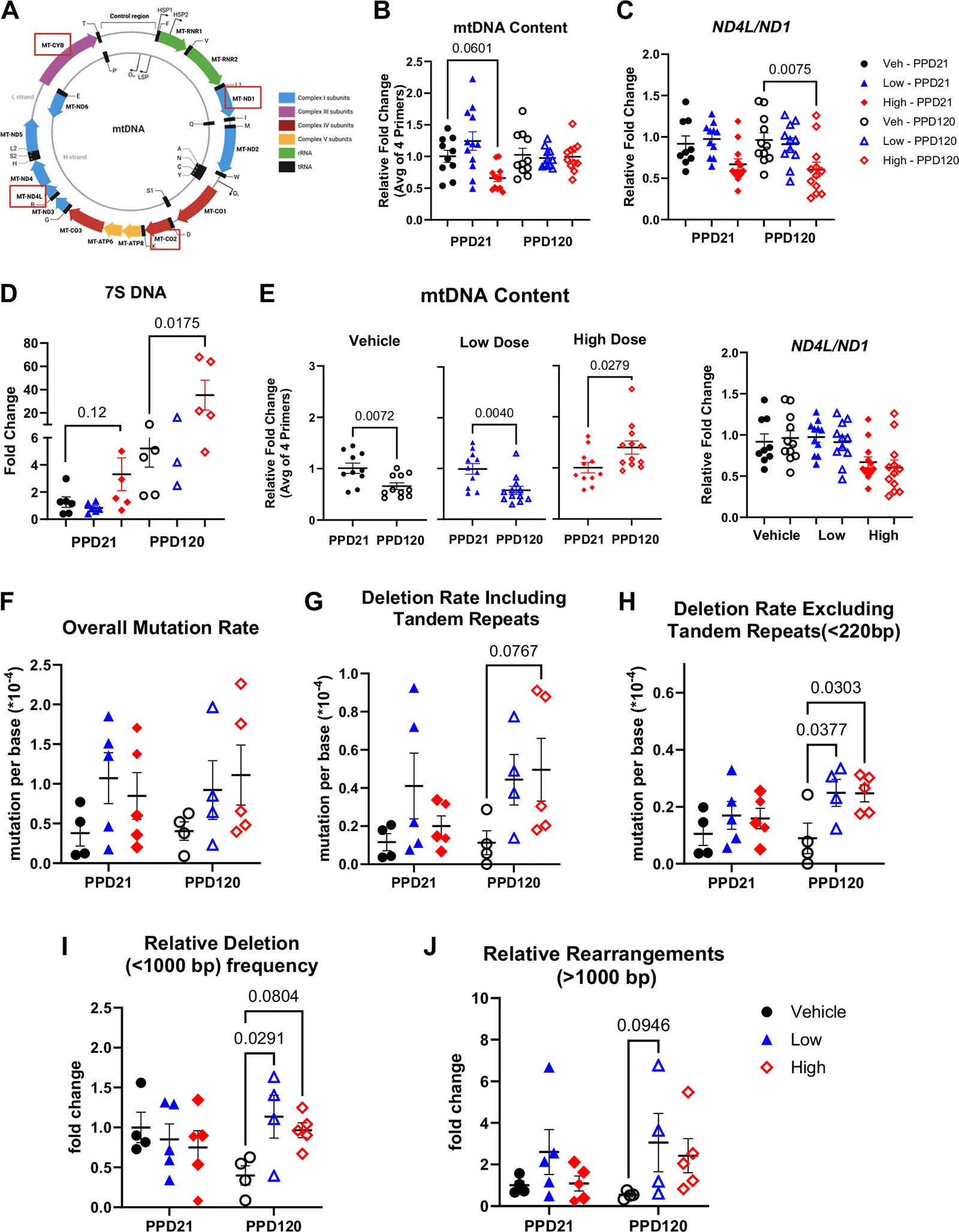
cART causes mitochondrial DNA (mtDNA) depletion and mutations. **A** Diagram of the mammalian mitochondrial genome with the location of genes analyzed indicated with purple rectangles. Created in BioRender. Taube, N. (https://BioRender.com/bcqioac). **B** mtDNA content as pooled data from individual primers for each animal relative to the nuclear genes coding for 18S, **C** *ND4L/ND1*, and **D** 7S DNA measured via qPCR at PPD21 and PPD120, analyzed within timepoints across dose groups. **E** mtDNA content and *ND4L/ND1* analyzed across timepoints within dose group. All PCR data were analyzed via one-way ANOVA, data shown as mean ± SEM (*n* = 12 rats/group). mtDNA was deep-sequenced and mutation rates determined between cART-treated to vehicle-exposed animals at each time point. **F** The overall mutation rate was quantified as the ratio of variants occurrence per base. Deletion rates were quantified as the ratio of their occurrence to the total number of bases across the reads, including (**G**) or excluding (**H**) the sites of tandem repeats. The relative frequency of deletions of <1000 bp (**I**) or >1000 bp (**J**) was quantified as the ratio of reads having the deletion to the total number of reads, normalized to the mean of the untreated group at PPD21. The results were analyzed via two-way ANOVA, data shown as mean ± SEM (n = 5 rats/group).

The exact timing and molecular mechanisms driving pregnancy-associated RR remain poorly understood, but studies show that hormonal and metabolic rewiring, including of mitochondria, are involved^2,24^. As two out of the three drugs used in our cART are NRTIs, many of which have historically been shown to cause mitochondrial DNA (mtDNA) depletion and/or deletions^15,25^, we hypothesized that changes in mtDNA content and metabolism may be associated with the cardiac effects observed. To test this, we first determined the effects of the drugs on mtDNA content. We isolated heart genomic DNA and used primers covering different mtDNA regions (Fig. 3A). Data were normalized to a nuclear gene (*18S*) and, given similar trends (Supplementary Fig. 3A), were pooled to report on the entire genome as the average fold change of all primers for each animal. The HD group showed decreased mtDNA content at PPD21, with no changes observed between groups at PPD120 (Fig. 3B). Data from ND4L, which resides within the site of the common deletion^26^, were normalized to ND1 and used as a proxy for the occurrence of mtDNA deletions. Loss of the ND4L signal relative to ND1 was significant only in the HD group animals at PPD120 (Fig. 3C), suggesting the presence of mutated genomes missing that locus.

An increased steady state of deletion-bearing mitochondrial genomes could trigger mtDNA biogenesis as a compensatory response, which can explain the lack of mtDNA content changes at PPD120 despite the continued presence of the drugs (Fig. 3B). Consistent with this, levels of 7S DNA, a tri-strand intermediate that originates during initiation of mtDNA replication and often used as proxy for mtDNA biosynthesis, was found to be significantly increased in HD-treated hearts (Fig. 3D). When looking at mtDNA content as a function of time (pregnancy vs post-pregnancy physiology), we found lower mtDNA copy number between PPD21 and PPD120 in vehicle and LD animals, but not in the HD group which showed an increase in mtDNA abundance (Fig. 3E). Thus, the drugs had effects on mtDNA content that went beyond those observed during physiological cardiac RR. The distinct effects on copy number observed on the HD-dosed animals suggest compensatory mtDNA biogenesis as a mechanism attempting to support RR.

Whereas the effects of NRTIs on mtDNA content are well established, it remains unclear whether they also induce mtDNA mutations beyond the deletions previously identified by PCR-based methods^15^. This largely reflects the relatively recent availability of deep sequencing approaches for comprehensive mitochondrial genome analysis. To determine whether the cART used in this study induced mtDNA mutations, we performed deep sequencing of cardiac mtDNA. Due to limited sample availability, we employed a PCR-based strategy involving amplification of the entire mitochondrial genome prior to sequencing. Because all samples were processed under identical conditions, any PCR-induced errors would be expected to occur randomly and be evenly distributed across groups. To assess mutation burden, we applied a threshold criterion of ≥40% heteroplasmy, based on short variants including single- and multinucleotide substitutions, small deletions ( < 220 nt), and single-nucleotide insertions. Using this approach, we found that the proportion of animals exhibiting heteroplasmy above this threshold increased with both treatment dose and duration (Supplementary Fig. 3B). For instance, whereas 25% of animals in the vehicle control group met this criterion, 4 out of 5 animals in the high-dose group exceeded it (Supplementary Fig. 3B). Notably, high-frequency mutations were predominantly localized to the ND2, 16S rRNA, ATP6, and D-loop regions (Supplementary Table 1).

When examining overall mutation rates, we observed a trend toward increased mutation frequency in treated animals that did not reach statistical significance (Fig. 3F). Nonetheless, stratification by mutation type revealed a significant increase in deletion rates in treated animals compared to controls, particularly when categorized by deletion size and the presence or absence of tandem repeats (Fig. 3G–J). In contrast, no significant differences were observed in base substitutions (transitions and transversions), single- or multinucleotide insertions, or large ( > 1,000 bp) rearrangements (Supplementary Fig. 3C). Taken together, these results indicate that the cART use in this study not only alters mtDNA content but also increases mtDNA mutation burden primarily through short deletions ( < 1,000 bp). These combined effects likely alter mitochondrial function and contribute to the impaired/delayed cardiac RR observed in the treated animals.

### cART alters cardiac mitochondrial enzymatic function

**Fig. 4:**
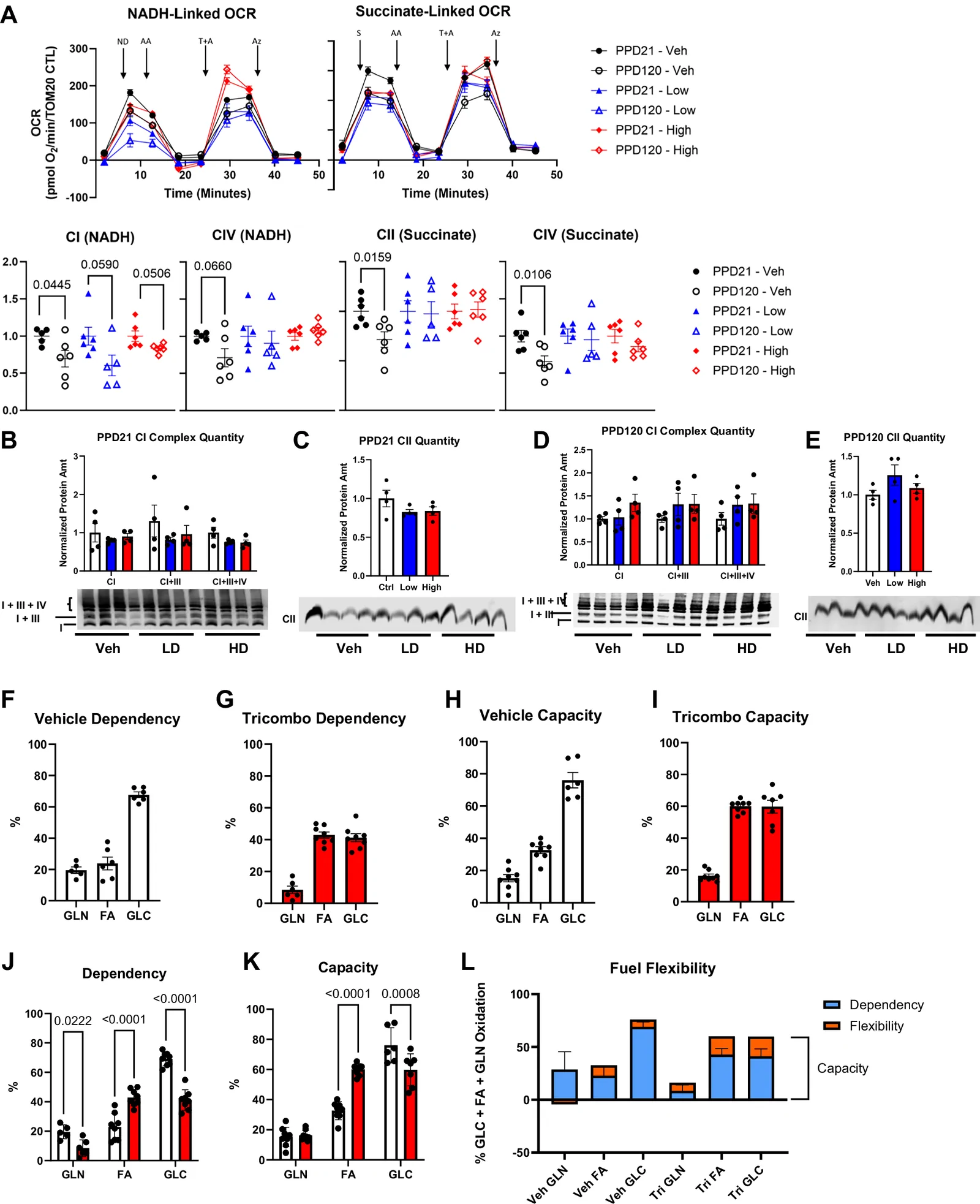
Mitochondrial function is altered in the maternal heart by cART exposure during and after pregnancy. **A** Oxygen Consumption Rates (OCR) traces measured in lysates from frozen heart tissue in PPD21 and PPD120 across timepoints using the Seahorse Flux Analyzer. Tracings over the course of the experimental protocol are shown for both NADH and succinate-linked respiration, as well as quantification of values; data were normalized to protein content based on protein levels of TOMM20 gauged in the same samples. Statistical significance was analyzed via two-sided t-test, data shown as mean ± SEM (*n* = 6 rats/group). **B** Mitochondrial protein quantification by BN-PAGE in heart tissue for complex I-containing complexes and (**C**) complex II for PPD21 and complex I (**D**) and complex 2 (**E**) PPD120. Data were analyzed via one-way ANOVA and are shown as mean ± SD (*n* = 4 rats/group). **F**–**L** hiPSC-CM fuel flex assay examining capacity, flexibility and dependency on glutamine (GLN), fatty acids (FA), and glucose (GLC) as carbon sources. Results were analyzed via one-way or two-way ANOVA as appropriate. Data are shown as mean ± SEM (*n* = 8 wells/group/treatment).

To determine whether the mtDNA changes identified affected mitochondrial function in a biochemically detectable manner, we adapted a published protocol for measuring oxygen consumption in frozen heart lysates^27^, normalizing values to mitochondrial content using either the matrix protein succinate dehydrogenase A (SDHA) or the outer mitochondrial membrane protein TOMM20 (Supplementary Fig. 4A) in the same sample. Because freezing disrupts mitochondrial membrane integrity and abolishes coupling and ADP control, this assay quantifies electron transport chain (ETC) enzymatic capacity rather than integrated, coupled mitochondrial respiration. Comparing PPD21 and PPD120 within each group, we observed a significant decline in oxygen consumption over time across all groups with complex I substrates; a similar decrease was seen in vehicle-exposed animals with the complex II substrate succinate (Fig. 4A). This is aligned with the decreases in mtDNA content observed in vehicle and LD groups and further supports that increased mtDNA content in the HD group is compensatory. We then analyzed changes within each time point comparing treated to control, which revealed no significant differences (Supplementary Fig. 4B). Blue-native PAGE (BN-PAGE) confirmed lack of change in mitochondrial complex I or II protein abundance or overall supercomplex assembly in the intact heart (Fig. 4B–E). This lack of change is also consistent with Western blot showing no change in autophagy marker LC3 (Supplementary Fig. 4C), the main pathway to recycle organelles in post-mitotic cells. These findings indicate that differences in mitochondrial function are largely qualitative; they are also consistent with the observed alterations in mtDNA content and integrity.

Next, we used transmission electron microscopy (TEM) to characterize impacts on cardiac ultrastructure with a primary focus on mitochondria. We found no significant differences in ultrastructure between the pregnant and post-pregnant state in the vehicle control animals (representative image in Supplementary Fig. 4D and summarized pathologist report). Conversely, mitochondria and other features of the cardiomyocyte structure were morphologically altered in both doses, although the number of altered mitochondria and degree of overall ultrastructural changes were more pronounced as the cART dose increased (representative images on Supplementary Fig. 3D). To provide quantitative insights into mitochondrial ultrastructural changes, we first manually measured mitochondrial perimeter at PPD21 and used these measurements to benchmark and validate an artificial intelligence (AI)–assisted approach for quantifying mitochondrial area. Despite the use of different metrics, both methods consistently revealed increased mitochondrial size in HD samples (Supplementary Fig. 4E and F). Based on this concordance, we applied the AI-based analysis at PPD120, which identified a significant increase in mitochondrial area at this time point (Supplementary Fig. 4F). Interestingly, HD samples also exhibited increased glycogen granules (see summarized pathologist report). The concurrent enlargement of mitochondria and accumulation of glycogen suggests a coordinated metabolic adaptation to energy stress. It is worth noting that mitochondria from HD animals had increased cristae width, potentially indicating mitochondrial swelling and maladaption to stress.

Under normal physiological conditions, adult cardiac mitochondria preferentially utilize fatty acids, ketone bodies, amino acids, and lactate, respectively, for bioenergetics, with glucose serving as the primary fuel under stress. During pregnancy, the heart increases its metabolic flexibility to support remodeling, and postpartum cardiac mitochondria were recently shown to respire more efficiently on carbohydrates and amino acids than on other substrates^28^. As glycogen can be mobilized during periods of high demand to supply glucose for mitochondrial bioenergetics and support cardiac function^29^, its accumulation in the treated hearts indicate reduced mitochondrial utilization of carbohydrates. To test this, we treated iPSC-derived cardiomyocytes with cART as described in Fig. 2 and assessed their fuel dependency and flexibility using the Seahorse Flux Analyzer. This analysis showed that, although both control and cART-treated cells could respire using multiple substrates, including fatty acids, amino acids, and glucose, cART-treated cells displayed increased capacity for and reliance on fatty acid oxidation, alongside reduced flexibility and dependence in using carbohydrates as a carbon source (Fig. 4F–L, Supplemental Data 4G). These findings suggest that impaired metabolic flexibility in glucose utilization may contribute to the defective cardiac RR induced by cART.

### Transcriptional profiles reveal effects on cardiac mitochondrial and lipid metabolism

**Fig. 5:**
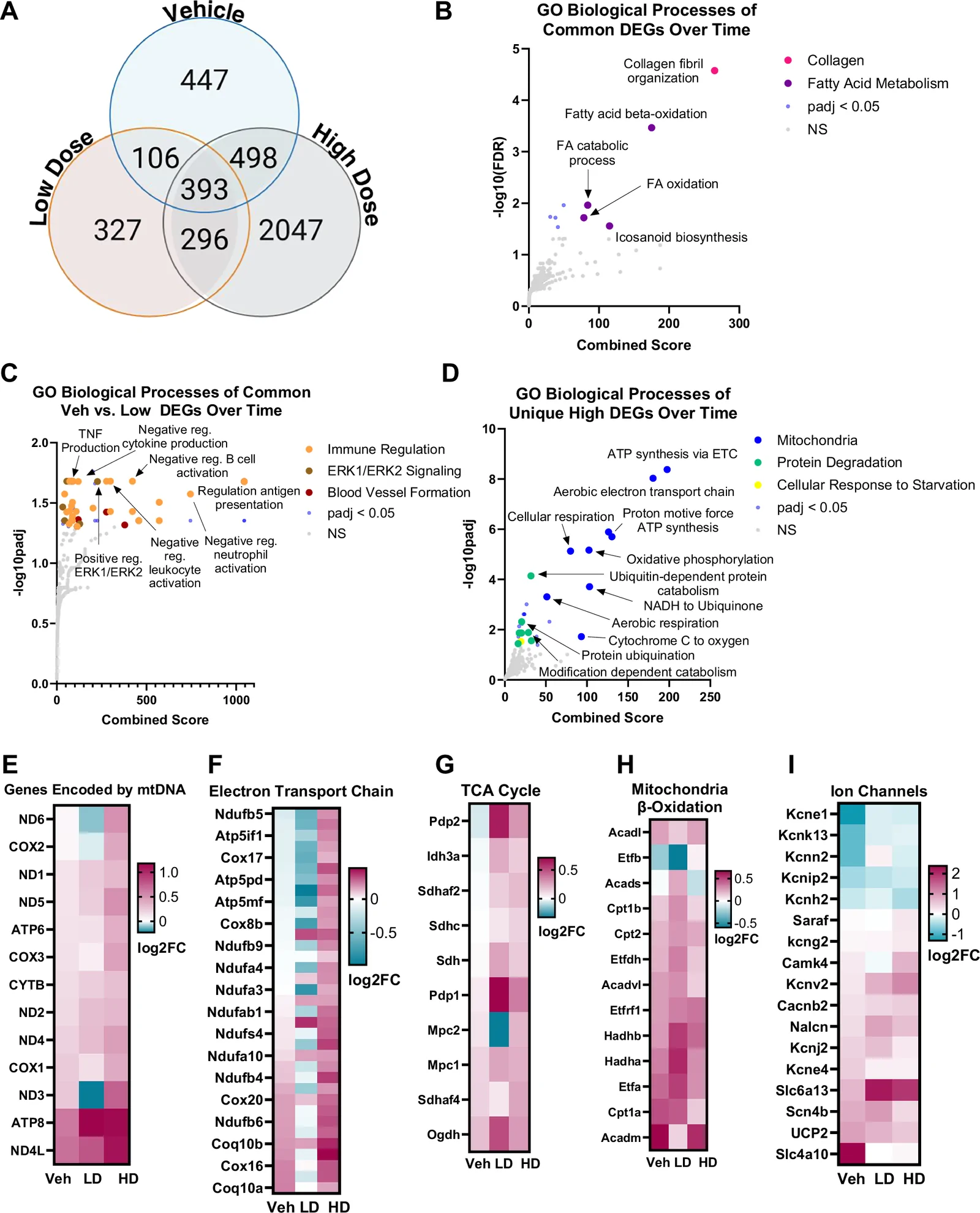
Significant transcriptional remodeling, including in the mitochondria, follows cART exposure in cardiac tissue. **A** Venn diagram depicting the overlap of differentially expressed genes (DEGs) identified using RNA-seq in vehicle-, LD- and HD-treated animals. Statistical significance was determined by DESeq2 with a Benjamini Hochberg multiple testing correction (n = 6 rats/group/timepoint, FDR < 0.05). Created in BioRender. Taube, N. (https://BioRender.com/6882bt3). GO biological processes analyzed from DEGs (**B**) common to all three groups, **C** common to vehicle and low dose groups and (**D**) unique to HD-treated animals over time (PPD21 vs. PPD120). Analyzed via Enrichr, (*n* = 6 rats/group/timepoint, adjusted *p* value < 0.05). Heatmap depicting fold differences in the expression of genes between (PPD21 vs. PPD120) shown as log2 fold change (log2FC) associated with: (**E**) mtDNA, **F** electron transport chain, **G** TCA cycle, **H** mitochondria beta-oxidation and (**I**) ion channels. Genes with FDR < 0.05 were considered statistically significant (*n* = 6 rats/group/timepoint). Data depicted on heatmaps reflect genes that were significantly differentially expressed in at least one of the groups.

The phenotypes so far identified suggest that cART biochemically affects the heart, even when provided in low doses. As we previously showed that progressive mtDNA depletion altered the transcriptional landscape of cells in culture before any detectable signs of mitochondrial dysfunction^30,31^, we next profiled the heart transcriptome. We first established the physiological changes occurring between PPD21 and PPD120 in the vehicle-controls, then determined how the drugs affected those. We found 1444 genes that changed between PPD21 and PPD120 in the control animals (Fig. 5A). In LD-treated animals we found 1222 genes that were differentially expressed over time, while 3234 genes were identified in HD animals; common to all 3 groups were 393 genes (Fig. 5A). These common genes enriched for fatty acid metabolism and collagen (Fig. 5B). Genes that overlapped uniquely between the vehicle and LD animals (106) involved the immune system and ERK signaling (Fig. 5C), which is interesting because ERK has been associated with physiological and pathological cardiac hypertrophy^32–34^. Only 296 genes were common exclusively between the LD and HD (Fig. 5A), and while they involved collagen fibril organization and fatty acid metabolism, we did not find significant similarities associated with mitochondria (Supplementary Table 2).

We next focused on the effects that were unique to each dose and found that the 327 genes that were solely changed in the LD-treated animals were associated with calcium homeostasis (Supplementary Table 2) while the 2,047 uniquely altered in the HD enriched primarily for mitochondria and cellular response to starvation (Fig. 5D). The latter finding is notable given the EM-observed glycogen accumulation in the heart and the reduced capacity of treated iPSCs to utilize glucose, suggesting that despite abundant fuel availability, cardiomyocytes in treated animals were not effectively using available carbon sources. All transcripts from the mtDNA (Fig. 5E) and several nuclear-encoded ETC genes were significantly upregulated in HD- but not LD-treated animals (Fig. 5F), consistent with the changes observed at the mtDNA level (Fig. 3B–E). We also found differential expression of tricarboxylic acid (TCA) cycle and lipid oxidation genes based on the treatments, with mitochondrial beta oxidation seemingly activated in the LD but not in the HD when compared to the vehicle (Fig. 5G–H, and Supplementary Table 2). Considering the differential effects on OXPHOS and beta oxidation with similarities in TCA cycle enzymes, these data suggest distinct fuel utilization by the mitochondria of cardiomyocytes based on the cART dose to which animals were exposed. This is in line with the differential effects of the LD and HD on mitochondrial function over the same period (Fig. 4A). Interestingly, genes and pathways that we previously found to be causally linked to the broader effects of mtDNA depletion in cell culture^30,31^ were also found to be differentially regulated in the treated animals (Supplementary Table 2), indicating important similarities in the broader transcriptional response to mtDNA depletion in vitro and in vivo.

Several genes coding for ion channels and solute carriers were also differentially regulated between the vehicle and the treated groups (Fig. 5I and Supplementary Table 2). The directionality of these changes suggests an increase in the intracellular Na⁺ pool, which could alter cardiac excitability and ion homeostasis, providing a potential mechanistic explanation for the observed ECG phenotypes (Fig. 2O–Q). Notably, the largest differences in gene expression relative to vehicle-exposed controls involved transcripts from *KCNE1* (potassium voltage-gated channel subfamily E regulatory subunit 1) and the sodium bicarbonate exchanger *SLC4a10* (Fig. 5I) – both of which have been associated with long-QT syndrome^35^. Taken together, the RNA-seq data demonstrate the broad transcriptional remodeling that occurs in the heart as it transitions from pregnancy-associated to post-pregnant physiology. Importantly, these data revealed that chronic drug exposure had unique effects, including on mitochondria and their metabolism. Moreover, the similarities and differences in the directionality and magnitude of gene expression changes between the LD and HD groups raise the possibility that the more severe cardiac alterations observed in the latter are linked to the extent of cART-induced mitochondrial effects.

### Cardiac lipid metabolism is significantly modulated by cART

**Fig. 6:**
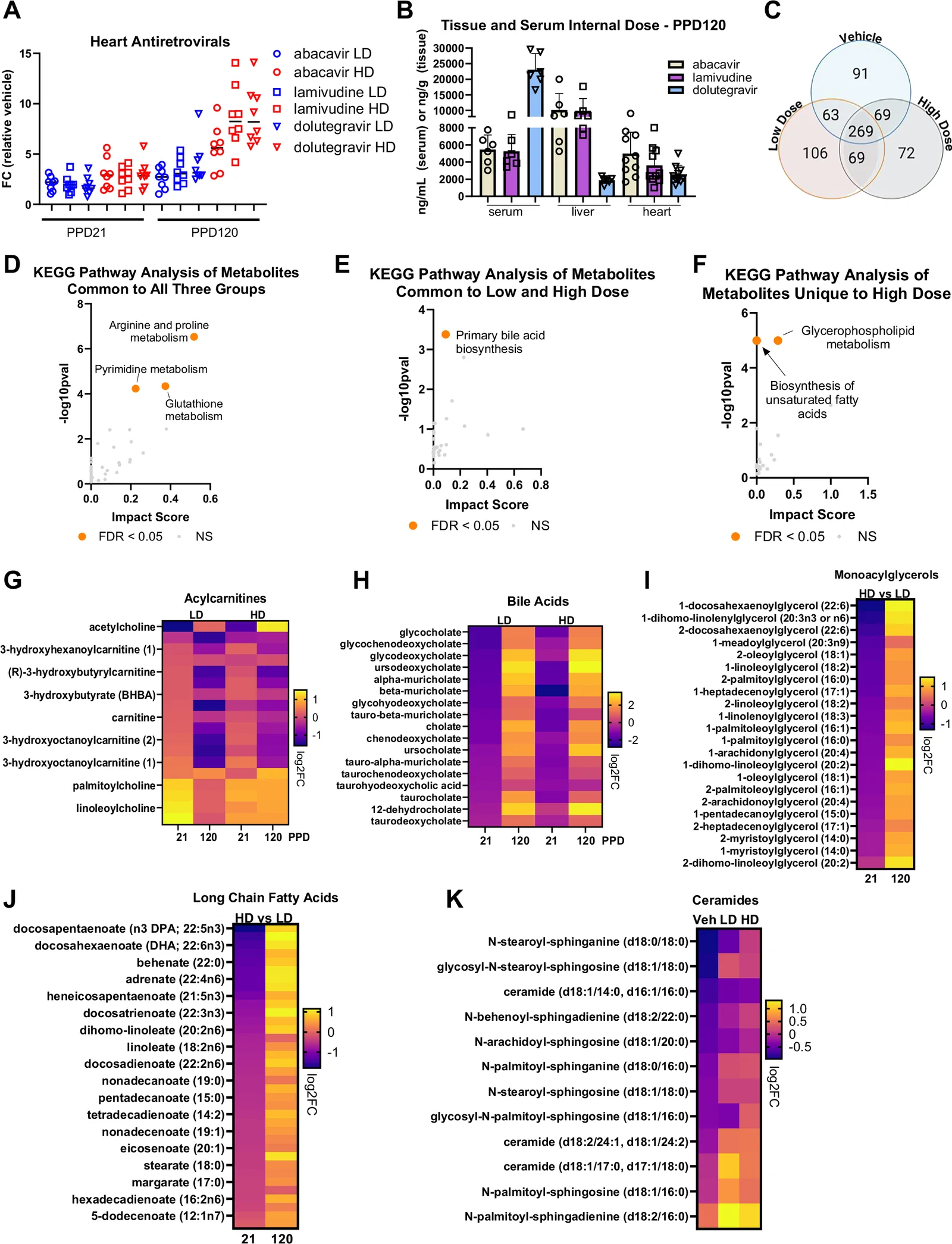
Metabolomics analysis indicates significant lipid remodeling in the heart, including the accumulation of bile acids, by cART. **A** Estimated levels of antiretrovirals shown as fold change detected in the heart by mass spectrometry at PPD21 and PPD120 in animals treated with the LD and HD cART (*n* = 8 hearts/group/timepoint). **B** Internal dose of ABC/DTG/3TC in the serum, heart and liver of animals exposed to the HD at PPD120. Data are shown as mean ± SD (*n* = 10 rats/group/timepoint). **C** Venn diagram depicting overlaps of metabolites significantly changed over time (PPD21 vs. PPD120) in vehicle-, LD and HD-treated animals. Differential metabolite abundance was assessed using ANOVA on log-transformed metabolomics data, and fold changes were calculated relative to the control group; metabolites with an adjusted significance level (FDR < 0.05) are shown. Created in BioRender. Taube, N. (https://BioRender.com/eluhunq). KEGG pathway analysis of differentially expressed metabolites (**D**) common to all three groups (**E**) common to LD and HD, and (**F**) unique to HD-exposed rats, determined via Metaboanalyst (adjusted *p* value < 0.05, *n* = 8 rats/group/timepoint). Heat maps depicting fold changes in LD- and HD-exposed animals at PPD21 and PPD120 for (**G**) acyl-carnitines and (**H**) bile acids. Heat map depicting difference in levels of metabolites (as log2 fold change) when comparing HD- vs LD-treated animals at PPD21 and PPD120 for (**I**) monoacylglycerols and (**J**) long chain fatty acids. Heat map depicting the changes between PPD21 vs. PPD120, each individual group for (**K**) ceramides. (FDR < 0.05, *n *= 8 rats/group/timepoint). Data depicted on heatmaps reflect metabolites that were differentially enriched in at least one of the groups.

The pronounced transcriptional impact on mitochondria observed in HD-treated animals, together with reported cardiometabolic effects of ART^9^, prompted us to profile the cardiac metabolome. Using untargeted mass spectrometry, we were able to detect the drugs in cardiac tissue when comparing vehicle to dosed groups, with apparent accumulation between PPD21 and PPD120 (Fig. 6A). Because the same daily dose was delivered by gavage, we hypothesized that this accumulation might reflect changes in blood volume between the pregnancy (PPD21) and post-pregnancy (PPD120) states^36^. The relationship between plasma and tissue concentrations of ART, especially when administered as the tri-drug combination used clinically, remains poorly understood. To address this, we leveraged a different mass spectrometry method we recently validated to quantify ABC, 3TC and DTG in biological samples^37^, allowing us to gain quantitative insights into tissue-level drug exposure. The liver was included in this analysis because it is the first site of metabolism and detoxification of exogenous drugs. Although heart samples were available from both doses and time points, serum and liver were only available from animals at PPD120. Despite this limitation, analysis of these samples provided valuable comparative tissue-level information. We found that the NRTIs were present in similar quantities in the liver, heart and serum (Fig. 6B, compare tan and violet bars), though cardiac levels were lower than in the liver. In contrast, DTG (blue bar) was primarily detected in the serum, which is consistent with previous literature demonstrating it is 90% plasma bound^38^. With levels of DTG in other tissues ~10-fold lower than in serum (Fig. 6B), we surmise that it is unlikely to be a significant contributor to the observed cardiac phenotypes.

Using metabolomics analysis, we identified a total of 736 metabolites in the heart that were changed among the 3 groups, of which 269 were common to all (Fig. 6C). These shared metabolites enriched for amino acids, nucleic acids and redox metabolism (Fig. 6D, Supplementary Table 3), representing normal changes associated with physiological RR that were not affected by the drugs. Common to the two treated groups were only 69 metabolites that were surprisingly associated with primary bile acids biosynthesis (Fig. 6C, E), whereas HD-treated animals exhibited additional changes in lipid species (Fig. 6C, F). Thus, unlike the transcriptional effects where the drugs had impacts that went well beyond the normal changes associated with the transition from pregnancy (PPD21) to post-pregnancy physiology (PPD120), at the metabolome level, the effect of the drugs was more muted. Nevertheless, several unique responses were noteworthy. Accumulation of acyl-carnitines and bile acids was observed in both treated groups, with more pronounced effects in HD-treated animals at PPD120 (Fig. 6G, H); several intermediates associated with glycogen metabolism were also identified (Supplementary Table 3). Changes in phospholipids were prominent in the treated animals (Supplementary Table 3), which is notable because lysophospholipids have been linked to inflammation, dyslipidemia and increased CVD in patients living with HIV^39^. Conversely, monoacylglycerols (Fig. 6I) and long-chain fatty acids (Fig. 6J) were decreased at PPD21 in HD- relative to LD-treated animals, but were significantly increased by PPD120. This lipid accumulation may result from increased synthesis, decreased utilization, augmented lipolysis, and/or altered gut absorption. Ceramide levels were elevated in treated animals (Fig. 6K), consistent with increased triglyceride breakdown. Altered lipid metabolism, including through changes in lipolysis, has been shown to contribute to cardiac dysfunction^40^.

### cART-induced changes in liver metabolism impact systemic lipid levels

The detection of bile acids in the heart was unexpected because these lipids are generated in the liver and secondarily metabolized in the gut, after which they return to the liver via the portal vein to complete the enterohepatic circulation. However, a small amount of bile acids can escape this cycle, entering the systemic circulation thus reaching the heart^41^. As accumulation of bile acids in the heart has been shown to alter cardiac function, including through the inhibition of mitochondrial fatty acid beta oxidation^42^, these data suggested that, in addition to directly affecting the heart, cART utilized also affected the liver. More importantly, it led to the hypothesis that the combined effects of cART in both tissues influenced cardiac function. To test this, we started by evaluating liver-related clinical chemistry parameters. While we only had serum and liver available from animals at PPD120, we note that bile acids accumulated in the heart between PPD21 and PPD120 (Fig. 6H). Thus, we presumed that liver function, if altered, would be affected throughout the experimental timeline.

**Fig. 7:**
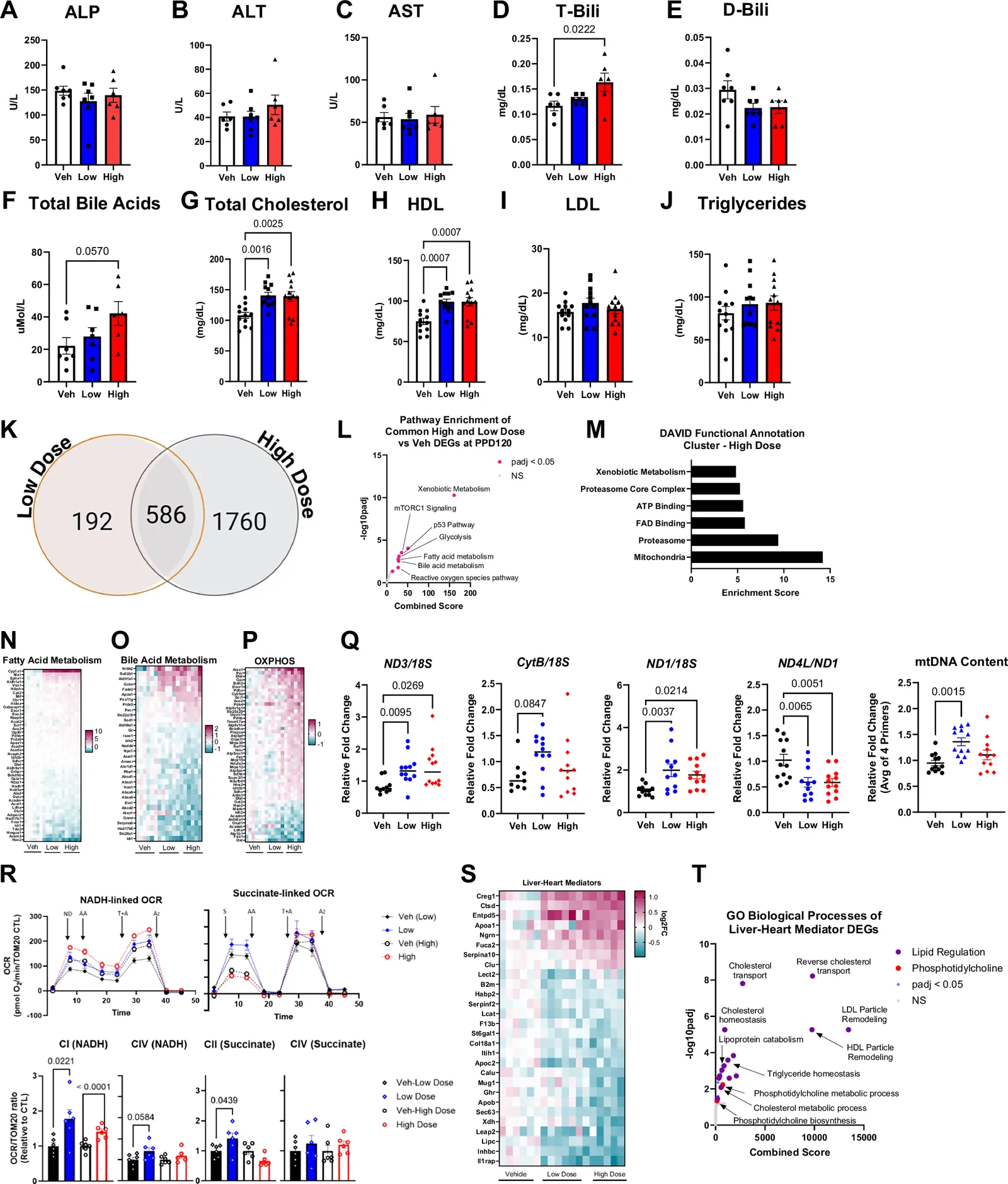
cART administration alters metabolism and mitochondria in the liver, leading to altered plasma lipid levels. Clinical chemistry measurements on serum collected at PPD120 for the HD group (**A**) alkaline phospohotase (ALP), **B** alanine transaminase (ALT), **C** aspartate transferase (AST), **D** total bilirubin (T-bili), **E** direct bilirubin (D-Bili), **F** total bile acids, **G** total cholesterol, **H** high-density lipoprotein (HDL), **I** triglycerides and (**J**) low-density lipoprotein (LDL). Results were analyzed via one-way ANOVA with multiple comparisons. Data are shown as mean ± SD (n = 7 rats/group/timepoint). **K** Venn Diagram of unique and overlapping liver genes differentially regulated by cART at the LD or HD relative to vehicle controls. Created in BioRender. Taube, N. (https://BioRender.com/3jsze73). **L** Pathway analysis of genes that were common between the two doses using MSigDB Hallmarks analyzed for pathways via Enrichr (adjusted *p* value < 0.05). **M** Functional annotation clustering (FAC) using DAVID based on RNA-seq from liver at PPD120 and heat maps showing differences as fold-changes relative to vehicle for (**N**) fatty acid metabolism, **O** bile acid metabolism and (**P**) oxidative phosphorylation (FDR < 0.05, *n* = 6 rats/group). **Q** mtDNA content assessed by qPCR as in Fig. 2A. Data were analyzed via one-way ANOVA and shown as mean ± SEM (n = 12 livers/group). **R** OCR measured in frozen liver tissue from HD-treated animals at PPD120. Tracings over time shown for both NADH and succinate-linked respiration, as well as quantification of values, accounting for mitochondrial content based on levels of TOMM20 in the same samples. Values were analyzed via two-sided t-test, data shown as mean ± SEM (*n* = 6 livers/group). **S** Heatmap of liver-heart mediators identified via RNA-seq in liver as per Cao et al.^45^. **T** GO biological processes pathway analysis using the identified differentially expressed liver-heart mediator genes shown in (**Q**), analyzed via Enrichr (adjusted *p* value < 0.05, *n* = 6 livers/group).

Using serum from PPD120 animals, we assessed liver function by monitoring the activity alkaline phosphatase (ALP), aspartate aminotransferase (AST), and alanine aminotransferase (ALT). We found no significant changes in enzymatic function, indicating an absence of hepatocellular injury (Fig. 7A–C). These findings are consistent with the pathology data that did not identify gross histological changes, and suggest that drug treatment did not cause overt liver damage. In contrast, total bilirubin was mildly increased in the HD-treated group (Fig. 7D), without significant changes in the conjugated (direct) fraction (Fig. 7E), alongside borderline increases in total circulating bile acids (Fig. 7F). Together, these findings point to mild cholestasis and/or perturbations in bile acid metabolism. We also observed increases in total and high-density lipoprotein (HDL) cholesterol (Fig. 7G, H), with no significant changes in low-density lipoprotein (LDL; Fig. 7I) or triglycerides (Fig. 7J). Notably, rodents and humans differ in cholesterol transport. Whereas humans primarily carry cholesterol in LDL particles, rodents predominantly rely on HDL due to the absence of cholesterol ester transfer protein (CETP), which mediates lipid exchange between lipoproteins^43,44^. Collectively, these data highlight a close concordance between cardiac metabolic remodeling and systemic lipid alterations in the circulation.

Next, we analyzed the transcriptome of the livers using RNA-seq. Comparing LD and HD-treated livers to vehicle controls, we identified a total of 2538 genes that were differentially expressed in the treated animals (Supplementary Table 4); about 586 genes were common to both groups while 192 genes were unique to the LD group and 1760 DEGs were solely identified in the HD (Fig. 7K). Pathway enrichment analysis of genes shared between the LD- and HD-treated groups revealed significant enrichment in xenobiotic, fatty acid and bile acid metabolism, as well as glycolysis (Fig. 7L). In contrast, analysis of the 1760 genes unique to the HD group identified mitochondria as the most prominently enriched pathway (Fig. 7M), whereas no significant pathway enrichment was observed among the 192 genes uniquely modulated in the LD group (Supplementary Table 4). Transcriptional changes in hepatic fatty acid (Fig. 7N) and bile acid metabolism genes (Fig. 7O) aligned with the elevations in circulating lipids and bile acids detected in plasma. Although the largest fold changes were observed in fatty acid metabolism genes (Fig. 7N), ~7% of the differentially expressed genes were associated with mitochondrial pathways (Supplementary Table 4). Notably, 51 of these genes were involved in OXPHOS, suggesting altered hepatic mitochondrial function (Fig. 7P). At the mitochondrial level, mtDNA content was increased at PPD120 in treated animals, accompanied by decreased normalized ND4L levels (Fig. 7Q), suggesting that cART induced mitochondrial biogenesis as a compensatory response to damaged mtDNA also in the liver. Functional assessment in frozen mitochondrial-enriched lysates supported this interpretation (Fig. 7R).

A recent study leveraged a bioinformatics framework based on natural genetic variation to identify mediators of inter-organ communication. By analyzing the global transcriptomes of the heart and liver across 100 diverse inbred mouse strains, the authors identified a set of 100 candidate hepatic genes predicted to mediate liver–heart crosstalk. Using complementary genetic and molecular approaches, they further demonstrated a causal link between the secretion of a liver-derived protein and heart failure characterized by diastolic dysfunction with preserved ejection fraction^45^. To determine whether liver transcriptional changes might influence cardiac function under our experimental conditions, we interrogated our liver RNA-seq dataset against this candidate gene set. Remarkably, 30 of the 100 genes were differentially regulated in the livers of treated animals (Fig. 7S). Pathway enrichment analysis of these genes revealed a strong association with lipid-related processes (Fig. 7T). Taken together, these findings suggest that cART-induced impairment of postpartum cardiac physiological RR may not solely arise from direct drug effects on the heart. Rather, our data support a model in which perturbations in liver homeostasis, characterized by altered lipid and bile acid metabolism, mitochondrial stress responses, and activation of xenobiotic and OXPHOS pathways, contribute to a systemic milieu that may indirectly influence cardiac function, particularly in the context of pregnancy. While this supports the emerging concept of a liver-heart axis in drug-induced cardiometabolic dysfunction, our results take it a step further by implying that hepatic adaptation to mitochondrial stress could act as a mediator of long-term cardiovascular effects under chronic cART exposure.

## Discussion

In this study, we demonstrate that maternal cardiac function, particularly postpartum physiological RR, is impaired in the presence of ABC/DTG/3TC. Although ABC/DTG/3TC has recently been replaced as the preferred guideline-recommended therapy for pregnant women living with HIV, many patients remain on alternative cART during pregnancy, including regimens outside those recommended as first-line options in national guidelines. While some individuals require regimen changes because of adverse side effects, for patients who achieved strong viral suppression before pregnancy, expert guidance often supports continuing the existing regimen, even when it is not considered a first-line option for use during pregnancy. We find that the cardiac effects identified in this study were associated with alterations in mtDNA content and integrity, leading to impaired mitochondrial function. They were further accompanied by significant changes in lipid metabolism in both the heart and the circulation, pointing to additional contributions from the liver. Although serum clinical chemistry and histopathological analyses did not indicate overt hepatic injury, transcriptomic profiling revealed marked mitochondrial and lipid remodeling also in this tissue. Notably, these changes included differential regulation of mediators previously implicated in liver–heart crosstalk and diastolic dysfunction with preserved ejection fraction^45^ - phenotypes that, while subclinical, were observed herein.

Dyslipidemia is a common comorbidity in individuals living with HIV and is associated with an increased risk of CVD. While earlier epidemiological studies suggested that dyslipidemia was largely driven by the virus itself, supported by improvements in lipid profiles following initiation of cART^46,47^, more recent evidence has linked cART exposure to the development of dyslipidemia and adverse cardiometabolic outcomes^9^. Notably, ABC and 3TC have traditionally been considered to carry relatively low risk^46,47^, but it is possible that the metabolic changes associated with pregnancy create a permissive environment that exacerbates their effects Mechanistically, how cART can lead to dyslipidemia is poorly understood, but our transcriptional and metabolic data in the heart and the liver - in addition to internal dose measurements—strongly implicate NRTIs and their effects on mitochondria. Mitochondrial activity declined during physiological remodeling (PPD120) in the heart of vehicle-treated animals, which was not observed in HD-exposed counterparts and may have been influenced by activation of mtDNA biogenesis. The HD-treated animals also exhibited increased mitochondrial complex activity in the liver. These effects on mitochondria are likely compensatory in both tissues, although their functional implications and underlying cause may differ. Both the heart and liver rely on fatty acid oxidation for ATP production. However, the liver plays a central role in systemic metabolic regulation, including glucose production during fasting via gluconeogenesis and fatty acid synthesis during feeding through de novo lipogenesis. In addition, the liver possesses significant regenerative capacity, with hepatocytes able to proliferate in response to injury. Accordingly, changes in mitochondrial activity in the liver may reflect anabolic reprogramming or adaptations to altered lipid and bile acid metabolism induced by cART. In contrast, the heart is composed largely of terminally differentiated cardiomyocytes that rely on mitochondrial bioenergetics to sustain contractile function. Thus, compensatory mitochondrial changes in the heart are more likely to reflect increased energetic demand rather than anabolic remodeling.

The accumulation of acyl-carnitines and glycogen in the heart, together with the transcriptional signature of cellular starvation and decreased capacity of treated iPSC-derived cardiomyocytes to utilize glucose, suggest that the tissue is under metabolic stress with impaired utilization of both fatty acids and carbohydrates. In addition, the accumulation of free fatty acids, long-chain fatty acids, and monoacylglycerols is indicative of increased lipolysis and collectively points to a mismatch between substrate availability and utilization. Interestingly, bile acids, which were found to accumulate in both the circulation and the heart, are known to inhibit mitochondrial fatty acid oxidation, providing a potential additional mechanism by which mitochondrial activity may be suppressed under our experimental conditions. Although it remains unclear whether the specific bile acids identified here directly impair cardiac mitochondrial function, our studies in non-pregnant animals exposed to the same cART regimen revealed similar increases in circulating cholesterol and bilirubin, but not bile acids. Notably, these animals did not exhibit overt cardiac dysfunction beyond mild hypertrophy (Marchese, Taube et al., submitted). These findings raise the possibility that cART exerts differential effects on hepatic metabolism in the context of pregnancy or, alternatively, that pregnancy confers increased susceptibility. In this context, the observation that mitochondrial function shifts substrate flexibility during pregnancy and the postpartum period, favoring carbohydrates over fatty acids^28^ suggests that, in the absence of such metabolic remodeling, the effects of cART on the heart may be attenuated. These questions are the focus of ongoing investigations in the laboratory.

Beyond these findings, several questions arise from our results. For example, the degree to which the effects of the drugs on the mitochondria were causative of the cardiac phenotypes and how these may mechanistically mediate the local (heart) and systemic (likely driven, in part, by the liver) lipid remodeling needs further investigation. The dose dependency of the effects on mitochondria and on the cardiac phenotypes suggests that a causative relationship between those two is plausible. It remains unclear whether the postpartum cardiac changes reflect impaired or merely delayed pregnancy-associated RR, as animals were not monitored beyond 4 months postpartum. Extended longitudinal studies could reveal either full recovery or persistent cardiovascular deficits, particularly under continued cART exposure. Addressing these questions will be critical to fully understand the interplay between cART, mitochondrial function, and maternal cardiovascular health. Irrespective of these uncertainties, it is noteworthy that epidemiological studies have reported an increased prevalence of heart failure with preserved ejection fraction (HFpEF) in women living with HIV, particularly those on NRTI-containing regimens^48^. These patients also face a heightened risk of coronary artery disease, characterized by persistent immune activation despite viral suppression under cART, and increased plaque deposition^49^. Although it is not clear whether these outcomes derive from off-target effects of the drugs or from larger viral reservoirs in difficult to access tissues^49^, the absence of the virus or viral proteins in our experimental model suggests that the medications contribute to the observed cardiac phenotypes. Further studies in the presence of the virus will help define the extent to which cART may have additive or synergistic effects with HIV infection. Notably, recent evidence indicates that women with ≥ 5 live births are at greater risk of developing HFpEF later in life, highlighting how repeated cycles of physiological hypertrophy and postpartum RR when combined with a second hit, such as cART exposure, may further increased the susceptibility of women living with HIV to cardiovascular dysfunction^50^.

In summary, our results underscore the need to focus not only on offspring but also on maternal health in populations exposed to cART. While the observed cardiac phenotypes were mild and subclinical, they may become clinically relevant under additional stresses or environmental exposures. Limitations of this study include the absence of parallel human data and in vivo mechanistic analyses, which are difficult to perform in rats due to limited genetic tools, although this species provide a more cardiophysiologically relevant model for humans than mice. Despite these constraints, our findings fill key knowledge gaps and provide a foundation for future studies to investigate how cART affects maternal cardiovascular health, complementing its well-established benefits in reducing vertical transmission, lowering HIV-related morbidity and substantially improving quality of life.

## Methods

### Animals and cART treatment

Animal care and use were in accordance with the Public Health Service Policy on Humane Care and Use of Animals. All animal studies were conducted in an animal facility accredited by AAALAC International. Studies were approved by the Battelle (Columbus, OH) Animal Care and Use Committee (ACUC protocol T06055) and conducted in accordance with all relevant National Institutes of Health animal care and use policies and applicable federal, state, and local regulations and guidelines. Time-mated adult female Sprague Dawley rats, aged 11-14 weeks, were received from Envigo (Indianapolis, IN), housed individually during gestation and with their respective litters during lactation, until weaning (lactation day 21), after which they were group-housed. Animals were maintained under specific pathogen-free conditions in a 12 h light cycle with water and food ad libitum. Time-mated rats were dosed daily via oral gavage (5 mL/Kg) starting at gestational day 6 (GD6) until about 13 weeks after weaning, totaling 4.5 months. This timeframe was selected to begin at the start of implantation, continue through pregnancy and lactation and encompass the reverse remodeling period after pregnancy. Female rats were dosed with abacavir (ABC), dolutegravir (DTG) and lamivudine (3TC) in a combination ratio of 12:1:6, respectively, representative of clinical formulation. Formulations were prepared and analyzed for concentration using validated analytical methods by RTI International (Durham, North Carolina). All formulations were within ± 10% of target concentrations. Females were dosed with either aqueous 0.2% methylcellulose/0.1% Tween 80 (vehicle), 150 ABC/12.75 DTG/75 3TC mg/Kg (low dose) or 300 ABC/25 DTG/150 3TC mg/Kg (high dose). Prior to use in studies, the identity and purity of abacavir sulfate (Tecoland Corporation, Irvin, CA; purity ≥ 99.6%), sodium dolutegravir (Gojira Fine Chemicals, Inc. LLC, Bedford Heights, OH; purity ≥ 99.8%), and lamivudine (Tecoland Corporation, Irvin, CA; purity ≥ 99.9%) were confirmed by RTI International (Durham, North Carolina) using multiple analytical methods.

Cardiovascular assessments, electrocardiography (ECG) and echocardiography (ECHO), were performed in the animals prior to weaning ( ~ PPD20), at 4 weeks ( ± 7 days), at 8 weeks (±9 days), and at 13 weeks (PPD120) post weaning. Assessments within a target date were staggered based on assay throughput and selected to ensure adequate representation across dose groups. Once the animal order was established at the preweaning assessment, this order was repeated at Week 4, Week 8, and Week 13 timepoints to ensure the time between assessments for an individual animal was similar across all animals.

### Electrocardiogram

All animals were lightly anesthetized with isoflurane to effect (1.75%) via induction chamber and then maintained with ~1.5 to 3% isoflurane with a cone mask for the conduct of cardiovascular data collection. Both ECG and ECHO were collected in the same session. Three electrodes were attached to animals for lead II ECG monitoring and recorded on a computer using the IOX Data Acquisition system (EMKA Technologies). The following ECG parameters were recorded and assessed: PQ Interval (msec), QRS Interval (msec), QT Interval (msec) and QTcV (QT corrected for heart rate using Van de Water’s formula) (msec).

### Transthoracic echocardiography

Following stabilization under anesthesia (1.75%), left ventricular (LV) function and dimensions were monitored and recorded using a Vevo 3100 Ultrasound machine with a 25 to 55 MHz probe (FUJIFILM VisualSonics Corporation, Canada). Transthoracic two-dimensional (2D) B-mode, as well as 2D-guided M-mode, echocardiography was obtained from the long- and short-axis views at the level of papillary muscles. A simultaneous single-lead (e.g., lead II) ECG was obtained and recorded for the determination of cardiac cycle intervals during ECHO evaluation with the ECHO images and cine loops. The recorded images and cine loops were exported and analyzed offline. The following ECHO parameters were recorded and assessed: heart rate (beats per minute [bpm]), systolic diameter (mm), diastolic diameter (mm), systolic volume (µL), diastolic volume (µL), stroke volume (µL), ejection fraction (%), fractional shortening (%), cardiac output (mL/min), left ventricular (LV) Mass (mg), LV Mass Corrected (mg), systolic LV anterior wall thickness (mm), diastolic LV anterior wall thickness (mm), systolic LV posterior wall thickness (mm) and diastolic LV posterior wall thickness (mm).

### Hemodynamic assessment with dobutamine challenge

For the hemodynamic assessments, anesthesia was induced by isoflurane (up to 5%) via a gas chamber and maintained via face mask with 95% O_2_/5% CO_2_. Following anesthesia induction, the animal was shaved for vascular access and positioned in dorsal recumbency on a heated surgery table. Infiltration anesthesia at the incision site(s) (i.e., femoral triangle, jugular furrow, or ventral cervical region) was done with 2% lidocaine ( ~ 0.05 mL diluted with 0.9% NaCl) administered prior to the instrumentation. A tracheostomy was performed for ventilatory support, and the animal was ventilated ( ~ 100 breaths/min, ~2.5 mL tidal volume with 95% O_2_/5% CO_2_) with an adjustable small animal ventilator (Harvard Apparatus). A suitable vein (left or right jugular or femoral vein) was isolated and cannulated for the administration of dobutamine.

Transthoracic needle electrodes forming a single-lead ECG (e.g., lead II) were placed. To record arterial pressures, a 1.4 F or 2 F high-fidelity micromanometer catheter (Millar Instruments) was placed via a cut-down and inserted into a femoral artery or carotid artery and advanced towards the aorta. To record LV pressures, the right carotid artery was isolated, dissected free from the surrounding tissue, and cannulated with a 1.4 F or 2 F high-fidelity micromanometer catheter (Millar Instruments). This catheter was advanced retrograde across the aortic valve and into the LV chamber. A vein (i.e., the other jugular or femoral vein) was isolated and cannulated with an appropriately sized IV catheter or polyethylene (PE) tubing for drug administrations, as necessary. Heparin ( ~ 100 IU, IV) may be administered after the surgery. Following a baseline/hemodynamic stabilization period (up to 20 min), the overall cardiovascular responses were monitored for up to 120 min following baseline data collection.

Dobutamine challenges were administered by IV infusion for dams by intraperitoneal (IP) injection. Dobutamine was delivered in a dose escalation pattern at 0, 1, 3.33, and 10 µg/kg by IV infusion targeting 5–7 min at each dose. Hemodynamic recovery was allowed (up to 10 min) between each challenge. All animals were euthanized at the end of the experiment while they were under a surgical anesthetic plane. The following HEMO parameters were recorded and assessed for the left ventricle: Left Ventricle End Diastolic Pressure, Peak Left Ventricular Pressure (Pmax), dP/dt Max (dP/dt + ), dP/dt Min (dP/dt-), Contractility Index (CI), Tau (T_1/2_), Heart Rate (bpm), Systolic Ejection Period (SEP), Diastolic Filling Period (DFP), Contraction Time (CT) and Relaxation Time (RT). For Systemic Blood Pressure: Systolic Blood Pressure (mmHg), Diastolic Blood Pressure (mmHg), Mean Arterial Blood Pressure (mmHg) and Pulse Pressure (mmHg).

### Tissue harvest and collection

While under anesthesia (isoflurane), blood collection was performed using the catheter placed during cardiovascular assessment. For serum collection, blood was collected in serum separator tubes and kept on wet ice or ice packs until serum isolation, within 60 min of collection, using a refrigerated centrifuge at room temperature for at least 10 min at a relative centrifugal force of at least 1800g. Once the serum was isolated, it was saved in aliquots, and each sample was flash frozen in liquid nitrogen within 2 hours of collection and stored in a freezer set to maintain −85 to −60 °C. Following blood collection, while under anesthesia (isoflurane), animals were humanely euthanized via exsanguination. As soon as feasible following euthanasia, the heart was removed, weighed, rinsed with Phosphate Buffered Saline (PBS) and sectioned. The heart was bisected along its longitudinal axis parallel to the interventricular septum (IVS), with the IVS retained with the right side of the heart. The apex of the heart was removed from the left side and flash frozen. The remainder of the left side was flash frozen. The samples were maintained on dry ice until stored in a freezer set to maintain −85 to −60 °C. The right side of the heart was immersed in NBF for at least 24 h. After 24 h in NBF, the heart was removed, and a thin slice of the right ventricular free wall was transferred to glutaraldehyde and processed for ultrastructural analysis. The remainder of the right side of the heart was returned to NBF until fully fixed and processed for histopathology. For animals removed at 13 weeks after weaning, following collection of the heart samples, two samples ( ~ 250 mg total) from the left liver lobe were collected, cut into smaller pieces (~5 mm^3^), and flash frozen. The samples were maintained on dry ice until stored in a freezer set to maintain −85 to −60 °C.

### Human induced pluripotent stem cell-derived cardiomyocyte culture and MEA

Cryopreserved hiPSC-CM (iCell Cardiomyocyte^2^) from a female donor were obtained from FUJIFILM Cellular Dynamics (FCDI, Madison, WI, Cat. C1058). These cells were plated with iCell Cardiomyocytes Plating Medium (FCDI) and maintained in iCell Cardiomyocyte Maintenance Medium (FCDI) according to manufacturer’s protocols. For multielectrode array, cells were plated on Axion Biosystems CytoView MEA Plates (Axion BioSystems, USA) according to manufacturer’s protocols. Cells were treated with ABC/DTG/3TC at concentrations equivalent to 50% of each respective drug concentration quantified in serum, equating to 8 µg/mL / 9 µg/mL / 2 µg/mL, respectively. Cells were treated every 48 hours in iCell Maintenance Medium. After 7 days of treatment, cell functionality was analyzed via multielectrode array using Maestro Pro MEA platform (Axion Biosystems). Cardiac field potential recordings were performed using the Maestro Pro MEA platform (Axion BioSystems) at 37 °C and 5% CO_2_. Real-time data was collected simultaneously across all 768 electrodes (12.5 kHz sampling rate, 1–2000 Hz band-pass). Analysis was performed using AxIS Navigator software and the Cardiac Analysis Tool (Axion BioSystems). Local extracellular action potential (LEAP) recordings were performed using the Maestro Pro MEA platform (Axion BioSystems) at 37 °C and 5% CO_2_. Real-time data was collected simultaneously across all 768 electrodes (12.5 kHz sampling rate, 0.01–2000 Hz band-pass). LEAP induction was performed by enhancing cell coupling through electrical stimulus for 10 min using the AxIS Navigator software (Axion BioSystems). Analysis was performed using AxIS Navigator and the Cardiac Analysis Tool software (Axion BioSystems). After measurement, cells were harvested and snap frozen for further analysis.

### DNA isolation, mitochondrial copy number and deletions analyses by PCR

DNA was isolated from heart and liver tissue and hiPSC-CMs using DNeasy Blood and Tissue Kit (QIAGEN, Valencia, CA) following manufacturer protocol. DNA concentration was estimated in NanoDrop^TM^ 2000 and Qubit^TM^ 3 fluorometer. After isolation, DNA was diluted in DEPC water to a concentration of either 0.5 ng/µL (hiPSC-CM), 0.25 ng/µL (liver) or 0.05 ng/µL (heart) for qPCR analysis of mitochondrial genes as surrogates for mitochondrial copy number and mitochondrial genome deletions. PCR amplification was performed using primers for *18S, ND1, ND4L, CYTB, COX2* and *ND3*. Sequences for these primers can be found in the supplement (Supplementary Fig. 4A). PCR amplification was performed using CFX384 Real-Time PCR System (Bio-Rad, Hercules, CA) and analyzed using primer efficiencies. 7S DNA level was quantified as previously described^51^, adjusting the primers to the *Rattus norvegicus* mtDNA sequence. Briefly, ΔCt was calculated as the difference between the Ct corresponding to primers amplifying the 7S and mtDNA (7S-A and 7S-B1) and the Ct corresponding to primers amplifying mtDNA only (7S-A and 7S-B2) (Supplementary Fig. 4A), followed by normalization to the average value of the PPD21 control samples. The formula 2^(−ΔΔCt) was used to calculate the fold enrichment.

### Blue native PAGE

Experiments were performed using Native PAGE as described previously^52^. Briefly, 100 μg of mitochondrial fractions were thawed on ice for 30 min. Samples were mixed with digitonin 5% (w/v), 4X Native PAGE sample buffer, and a volume of deionized water to total 24 μL. Samples were incubated on ice for 20 min and centrifuged at 20,000 × *g* at 4 °C for 10 min. 20 μL of the supernatant was transferred into new tubes. 4 μL of Coomassie G-250 sample additive was added per sample, mixed, and 10 μL of each sample was loaded into two gels. Electrophoresis in Native PAGE™ 3−12% gels was performed in XCell™ SureLock™ with NativePAGE™ (NP) buffer and Coomassie Brilliant Blue G-250 (CBBG) 0.02% (w/v) in NP buffer (dark blue cathode) at 150 V for 30 min. After 30 min, dark blue cathode buffer was removed and replaced with CBBG 0.002% (w/v) in NP buffer (light blue cathode). A BN-PAGE gel was transferred to methanol-activated PVDF membranes in Dunn carbonate buffer (10 mM NaHCO_3_, 3 mM Na_2_CO_3_) while a secondary gel was used as a loading control for blot analysis. Transfer was performed at a current of 300 mA at 4 °C for 1 h using XCell™ Blot Module (Invitrogen™). CBBG was removed by washing with methanol. Membranes were blocked using EveryBlot Blocking Buffer (Bio-Rad©) and incubated overnight with either NDUFS1 (Cell Signaling, 1:1000, Rabbit mAb) for detection of complex I or SDHA (Cell Signaling, 1:1000, Rabbit mAb) for detection of complex II. Detection was performed in Odyssey® CLx Imager (LI-COR©) after 30-min incubation of secondary antibody (goat-anti rabbit IRDye 680RD, LI-COR 925-681817, 1:10,000). Full uncropped blots are available in source data, and each blot was repeated 2-3 times for accuracy of findings.

### SDS-Page and immunoblotting

Experiments for detection of LC3 were performed using SDS-Page as described previously^52^. Briefly, protein samples were lysed in cold RIPA with Halt™ Protease and Phosphatase Inhibitor Cocktail (Thermo Scientific^TM^) and centrifuged at 20,000 × *g* for 10 min. Supernatant was then mixed with NuPage LDS sample buffer (Invitrogen^TM^) and loaded into a 4–16% Tris-glycine SDS-PAGE, then transferred to a PVDF membrane. 40 µg of protein was loaded per well. Detection was performed in Odyssey® CLx Imager (LI-COR©). To determine presence of autophagy, blots were incubated with primary antibody for anti-MAP1LC3A (Millipore Sigma, L8918, 1:1000), HSP90 as housekeeping (Invitrogen, 37-9400, 1:1000) and secondary antibody for detection (goat-anti rabbit IRDye 800RD, LI-COR 925-681817, 1:10,000, donkey-anti mouse IRDye 680RD, LI-COR, 1:10,000). Full uncropped blots are available in Supplemental Information.

### MtDNA sequencing

mMDNA was enriched from the total heart DNA using REPLI-g Mitochondrial DNA Kit (Qiagen). The primer set was customized (Supplementary Fig. 4B) for use with *Rattus norvegicus*, according to manufacturer’s guidelines. Enriched samples were purified with the DNA Clean & Concentrator kit (Zymo Reserarch) and double stranded DNA was quantified with Qubit. Next Generation Sequencing was performed by Azenta Life Sciences, including transposase-based sheering and library prep followed by Illumina 2 × 150bp shotgun sequencing. Bioinformatic analysis was performed in the Geneious Prime® 2026.0.2 (https://www.geneious.com). Fastq files were paired, trimmed using BBDuk, and aligned to the *Rattus norvegicus* SR/Jr mitochondrion refence sequence (Accession GU997611) using Geneious mapper at highest sensitivity setting with detection of structural variants. Variants of minimum frequency set to 1% and minimum p-value of strand-bias >65% of 0.0001 were annotated. Only variants with average base quality equal to or greater than 30 were considered for further analysis.

### Oxygen consumption rate (OCR) in hiPSC-CM

OCR in intact cells was performed as previously described^52^. Briefly, OCR was assessed using the Seahorse XFe96 Analyzer (Agilent Technologies). Seahorse XFe96 Cell Cultured Microplates (Agilent Technologies) were coated with 50 µg/mL fibronectin and seeded with hiPSC-CM (10,000 cells/well) following manufacturer’s protocol (FIDC) and were incubated at 37 °C in a 5% CO_2_ humidified incubator. Cells were treated as previously described with ABC/DTG/3TC in the cell culture microplate every other day for 7 days, at which point they were washed twice by dilution with supplemented XF Assay medium and incubated in a O_2_-free BOD-type incubator at 37 °C for 1 h. The Fuel Flex protocol was used to assess mitochondrial capacity and dependency on different fuel sources. At the end of the assay, the medium was removed, and the cells were washed with PBS. PBS was removed, and cells were lysed with 20 µL of RIPA Lysis and Extraction Buffer supplemented with Halt™ Protease and Phosphatase Inhibitor Cocktail (Thermo Scientific^TM^). Protein content was estimated using Pierce™ BCA Protein Assay Kits (ThermoFisher™). OCR values were corrected by protein content of each given well. Each well was considered one independent sample. OCR results were transformed in O_2_ pmol/min/mg protein.

### OCR in frozen tissue

OCR was assessed in frozen cardiac lysate in a Seahorse XFe96 Analyzer (Agilent Technologies). Our group adapted a protocol that has been previously published detailing how to measure mitochondrial respiration in frozen biological samples^27^. Briefly, 20 mg of tissue was homogenized in physiologic buffer (70 mM sucrose, 220 mM mannitol, 5 mM KH2PO4, 1 mM EGTA, 2 mM HEPES) using an OMNI homogenizer with a soft tissue attachment (Omni International). Samples were spun down to enrich for a mitochondrial fraction and supernatant was collected. Protein content was estimated using Pierce BCA Protein Assay Kits (ThermoFisher) and used to standardize content across wells. Protein diluted in physiologic buffer was plated in Seahorse XFE96 Cell Culture Microplates. Before running, 115 µL of physiologic buffer + 11.8 µg/mL cytochrome C was added to every well. Final concentration of substrates and inhibitors was: NADH 1 mM or Succinate 5 mM + Rotenone 2 µM (Port A), Antimycin A (Port B), TMPD 0.5 mM + Ascorbate 1 mM (Port C), and Sodium Azide 50 mM (Port D) (all Sigma-Aldrich). OCR values were corrected by TOMM20 (Novus Biotechnology, NBP1-81556, 1:50) as determined by the ProteinSimple Jess System (Bio-Techne). The average of 6–8 replicates from the original lysate was considered one independent sample (*n* = 6). OCR results were transformed into O_2_ pmol/min/TOMM20.

### Transmission electron microscopy of cardiac tissue

Electron microscopy of cardiac tissue was performed as previously described^52^. Briefly, cardiac sections were fixed for 1 hour in fixative and washed twice in 0.1 M cacodylate buffer prior post-fixation in osmium tetroxide 1%. Tissue was rinsed in distilled water and dehydrated in an ethanol series, which transitioned to acetone. Samples were infiltrated with resin and polymerized, and blocks were cut, mounted on glass slides and stained in 1% toluidine blue in 1% sodium borate for quality check examination with a light microscope. After trimming, ultrathin sections were cut from blocks, placed in blocks and counterstained with 2% uranyl acetate and lead citrate. Using an FEI Co. Tecnai T12 transmission electron microscope operated at 80 kV, slides were visualized and digitally captured. For quantification, at least 100 images for each study group were captured, and EM subcellular analysis was made using Gatan Digital Micrograph software. All images were imported into the Artificial Intelligence (AI) based Image Analysis Software platform (Visiopharm). In each image, all whole mitochondria were manually segmented. Any mitochondria that were cut off by the edge of the image were not included. Additionally, if the same mitochondrion was seen in multiple images, only one instance of the mitochondrion was included. From these segmentations, the area was calculated for each mitochondrion using Visiopharm.

### RNA isolation, RNA-seq and gene expression analysis

RNA was extracted from frozen cardiac or liver tissue for RNA-seq using the RNeasy Kit (QIAGEN). The total RNA concentration was determined using the Qubit Flex Fluorometer (Thermo Fisher Scientific) and the RNA integrity was analyzed using an Agilent’s TapeStation 4200. Only samples with an acceptable RIN (8 or higher) were used. RNASeq libraries were created using Illumina’s TruSeq Starnded mRNA kit and verified using an Agilent TapeStation 4200. The RNAseq libraries were sequenced on Illumina’s NovaSeq platform at a read length of 75 bp and with a single end format. Single-end 76-cycle reads were aligned to the rat rn7 reference genome with STAR version 2.6. An average of 52 and 44 million uniquely mapped reads were obtained per heart and liver sample, respectively. Raw read counts were obtained using Rsubread version 2.14. Normalized counts and differentially expressed genes were obtained using DESeq2 version 1.42. For age-related comparisons, PPD120 heart samples were compared to PPD21 heart samples in the control, low dose and high dose groups. For treatment comparisons, livers at PPD120 were compared using vehicle control as the baseline vs low dose- or high dose-treated animals. Genes with a fold change ≥ 1.5 and FDR < 0.05 were considered statistically significant. Pathway enrichment analysis of differentially expressed genes was performed using Enrichr, focusing on Gene Ontology (GO) Biological Process annotations unless otherwise stated. Terms with an adjusted *p* value < 0.05 were considered significantly enriched, using the submitted differentially expressed gene list for analysis. Differentially expressed genes identified from RNA-seq analysis were also subjected to functional enrichment analysis using DAVID (Database for Annotation, Visualization and Integrated Discovery, v6.8). Gene Ontology and KEGG pathway terms were considered significantly enriched at a Benjamini–Hochberg adjusted *p* value < 0.05, using all expressed genes as the background set.

### Metabolomics

Metabolomic profiling was completed on hearts from dams at PPD21 and PPD120 and stored at −80 °C. Equal volumes of all samples were extracted and run across the Metabolon Inc. (Durham, NC) Precision Metabolomics discovery platform. Tissue extracts were prepared by automated methanol protein precipitation with the addition of recovery standards for quality control. Extracts were divided into four fractions and analyzed using complementary reverse-phase and hydrophilic interaction liquid chromatography (HILIC) ultra-performance liquid chromatography–tandem mass spectrometry (UPLC-MS/MS) methods in both positive and negative electrospray ionization modes. All analyses were conducted on a Waters ACQUITY UPLC coupled to a Thermo Scientific Q Exactive high-resolution/accurate-mass spectrometer equipped with a heated electrospray ionization (HESI-II) source and Orbitrap mass analyzer operated at 35,000 mass resolution. Quality assurance procedures included pooled matrix controls, process blanks, and internal standards to monitor instrument performance and analytical reproducibility. Raw data were processed using Metabolon’s proprietary informatics platform, and metabolites were identified by comparison with an in-house library of authenticated standards based on retention index, accurate mass, and MS/MS spectral matching. Metabolite abundances were quantified by area-under-the-curve peak integration, with abundance defined as peak area. Metabolon analysts performed additional quality control and curation to verify the consistency of metabolite identification and peak integration across samples. For multi-day analytical runs, data were normalized to correct for inter-day instrument variation.

Metabolomics data were log-transformed prior to statistical analysis. Unsupervised principal component analysis (PCA) was performed to assess overall metabolic variation and sample clustering. Differential metabolite abundance between experimental groups was evaluated using analysis of variance (ANOVA), with post hoc comparisons performed as appropriate. Statistical analyses were conducted using Metabolon’s standard bioinformatics pipeline and R-based analytical tools. Significance was defined as *p* < 0.05, with false discovery rate (FDR) correction applied where indicated.

### Clinical Chemistry

The following clinical chemistry parameters were analyzed using a Roche Cobas c311 chemistry analyzer (Indianapolis, IN): total cholesterol, low-density lipoprotein (LDL) cholesterol, high-density lipoprotein (HDL) cholesterol and triglycerides. Alkaline phosphatase (ALP), alanine aminotransferase (ALT), aspartate aminotransferase (AST), total bilirubin, direct bilirubin and total bile acids were analyzed using a Beckman Colter AU480 chemistry analyzer (Brea, CA).

### Statistical Analysis

All data was analyzed using GraphPad Prism Version 10.0.1 (La Jolla, CA). Statistical outliers were identified and excluded using the ROUT method (Q = 1%) for clinical cardiac data, clinical pathology and qPCR. When comparing two groups, an unpaired Student’s t test was used. Statistical comparisons between multiple groups were determined using an ordinary one-way or two-way ANOVA with multiple comparisons test as appropriate. All tests were two-sided. Significance was set for all experiments at p < 0.05.

### Reporting summary

Further information on research design is available in the Nature Portfolio Reporting Summary linked to this article.

## Supplementary information

Supplementary Information

Description of Additional Supplementary Files

Supplementary Data

Reporting Summary

Transparent Peer Review file

## Source data

Source Data

## Data Availability

The data generated in this study are provided in Supplementary Information/Source Data files. Metabolomics data is available in Supplemental Data 3. RNA-seq genomics data are deposited at the GEO and can be accessed through GSE325635, mtDNA sequencing data is available through the SRA accession number PRJNA1480161 [PRJNA1480161-SRA-NCBI]. Source data are provided with this paper.
