# Supplementary material for: Antiretroviral therapy in the peripartum period impairs post pregnancy cardiac reverse remodeling in a rodent model
Source: Nat Commun. 2026 Jul 17;17:8765. doi: 10.1038/s41467-026-75510-x (PMC13494002; doi:10.1038/s41467-026-75510-x)
Supplement: Supplementary file 1 [file 41467_2026_75510_MOESM1_ESM.pdf]

Supplementary Information

A

Dams	vehicle control	low dose	high dose
Group size	34	33	33
pregnant	25	27	27
fertility	0.735	0.818	0.818
littered	24	26	26
litter size (mean±SEM)	12.5±2.6	13.1±1.8	13.1±1.7
viable	24	26	26
fecundity	0.96	0.96	0.96
gestation length in days (mean±SEM)	22.3±0.1	22.2±0.1	22.4±0.1
**Pups**			
live births (% ±SEM)	93.13±2.16	96.47±1.23	86.11±4.08
survival (% ±SEM - PND0-1	94.38±4.16	88.35±4.17	88.30±2.85
PND1-4	99.73±0.27	97.37±0.97	96.65±0.99
PND4-7	100±0.0	100±0.0	99.48±2.55
PND7-12	100±0.0	100±0.0	100±0.0
live males (% ±SEM)	45.81±3.02	49.06±3.81	50.81±3.23
live females (% ±SEM)	54.19±3.03	50.94±1.29	49.19±4.14
average weight in grams at PND1 (mean ±SEM) - males	6.93±0.18	6.25±0.30	5.88±0.15
average weight in grams at PND22 (mean ±SEM) - males	53.06±0.96	47.45±1.40	45.14±1.58
average weight in grams at PND1 (mean ±SEM) - females	6.70±0.18	6.18±0.29	5.56±0.15
average weight in grams at PND22 (mean ±SEM) - females	52.53±1.27	46.83±1.46	43.55±1.39

B

Removal Reason(s): ALL<br>Summary: Average Grade (Mean)	Female		
	0 mg/kg	150/12/75 mg/kg	300/25/15 mg/kg
Number of Animals:	12	12	12
Number of Completed Animals:	12	12	12
**liver**			
Examined	12	12	12
No Visible Lesions	12	12	11
periportal; infiltrate, mononuclear cell	0	0	1
.... minimal	0	0	1
.... Average Grade	0.0	0.0	1.0
**lung**			
Examined	12	12	12
No Visible Lesions	0	2	3
subpleura; interstitium; inflammation; chronic	8	8	7
.... minimal	6	7	6
.... mild	2	1	1
.... Average Grade	1.3	1.1	1.1
alveolus; infiltration cellular; histiocyte	10	3	4
.... minimal	7	3	3
.... mild	3	0	1
.... Average Grade	1.3	1.0	1.3
blood vessel; infiltration cellular; eosinophil, perivascular	2	0	0
.... minimal	2	0	0
.... Average Grade	1.0	0.0	0.0
metaplasia; osseous	1	0	0

C

Removal Reason(s): ALL<br>Summary: Average Grade (Mean)	Female		
	0 mg/kg	150/12/75 mg/kg	300/25/15 mg/kg
Number of Animals:	12	12	12
Number of Completed Animals:	12	12	12
**lung (Continued...)**			
blood vessel; mineral	1	1	0
.... minimal	1	1	0
.... Average Grade	1.0	1.0	0.0
**diaphragm**			
Examined	12	12	12
No Visible Lesions	12	12	12
**mesenteric roll**			
Examined	12	12	12
artery 1; no visible lesions	9	8	8
artery 2; no visible lesions	10	9	8
artery 3; no visible lesions	11	10	9
artery 4; no visible lesions	12	10	9
artery 5; no visible lesions	12	10	9
artery 1; mineral	3	4	4
.... minimal	3	4	4
.... Average Grade	1.0	1.0	1.0
artery 2; mineral	2	3	4
.... minimal	2	3	4
.... Average Grade	1.0	1.0	1.0
artery 3; mineral	1	2	3

Removal Reason(s): ALL<br>Summary: Average Grade (Mean)	Female		
	0 mg/kg	150/12/75 mg/kg	300/25/15 mg/kg
Number of Animals:	12	12	12
Number of Completed Animals:	12	12	12
**mesenteric roll (Continued...)**			
.... minimal	1	2	3
.... Average Grade	1.0	1.0	1.0
artery 4; mineral	0	2	3
.... minimal	0	2	3
.... Average Grade	0.0	1.0	1.0
artery 5; mineral	0	2	3
.... minimal	0	2	3
.... Average Grade	0.0	1.0	1.0

D

Observation Type: All Types<br>Sex: Female<br>From Day 22 (Littering (A)) to 120 (Littering)	0 mg/kg	150/12.5/ 75 mg/kg	300/25/ 150 mg/kg
Number of Animals Examined:	12	12	12
Total Number of NAD Animals:	8	9	8
**Normal**			
Number of Animals Affected	12	12	12
% of Affected Animals	100	100	100
First to Last seen	22 - 118	22 - 118	22 - 118
**Alopecia, Dorsum**			
Number of Animals Affected	1	0	0
% of Affected Animals	8	0	0
First to Last seen	36 - 36	-	-
**Alopecia, Fore Limb**			
Number of Animals Affected	0	0	1
% of Affected Animals	0	0	8
First to Last seen	-	-	57 - 118
**Alopecia, Head**			
Number of Animals Affected	1	0	2
% of Affected Animals	8	0	17
First to Last seen	22 - 29	-	22 - 29
**Breathing, wheezing**			
Number of Animals Affected	0	1	0
% of Affected Animals	0	8	0
First to Last seen	-	67 - 67	-
**Coat Ruffled**			
Number of Animals Affected	0	1	0
% of Affected Animals	0	8	0
First to Last seen	-	67 - 67	-

Observation Type: All Types<br>Sex: Female<br>From Day 22 (Littering (A)) to 120 (Littering)	0 mg/kg	150/12.5/ 75 mg/kg	300/25/ 150 mg/kg
Number of Animals Examined:	12	12	12
Total Number of NAD Animals:	8	9	8
**Discharge, Eye, Right, Red**			
Number of Animals Affected	0	1	1
% of Affected Animals	0	8	8
First to Last seen	-	106 - 118	118 - 118
**Lethargic**			
Number of Animals Affected	0	1	0
% of Affected Animals	0	8	0
First to Last seen	-	67 - 67	-
**Maloccluded/Broken, Teeth**			
Number of Animals Affected	2	0	0
% of Affected Animals	17	0	0
First to Last seen	99 - 106	-	-
**Scab, Head, Left**			
Number of Animals Affected	0	1	0
% of Affected Animals	0	8	0
First to Last seen	-	118 - 118	-

Supplementary Figure 1. General phenotypic observations in dams administered ABC/DTG/3TC. Fertility and fecundity parameters (A), gross organ pathology (B) and clinical observations (C) from cohorts of Sprague-Dawley female rats exposed to two different doses of ABC/DTG/3TC for 4.5 months (n=12 rats/group/timepoint).

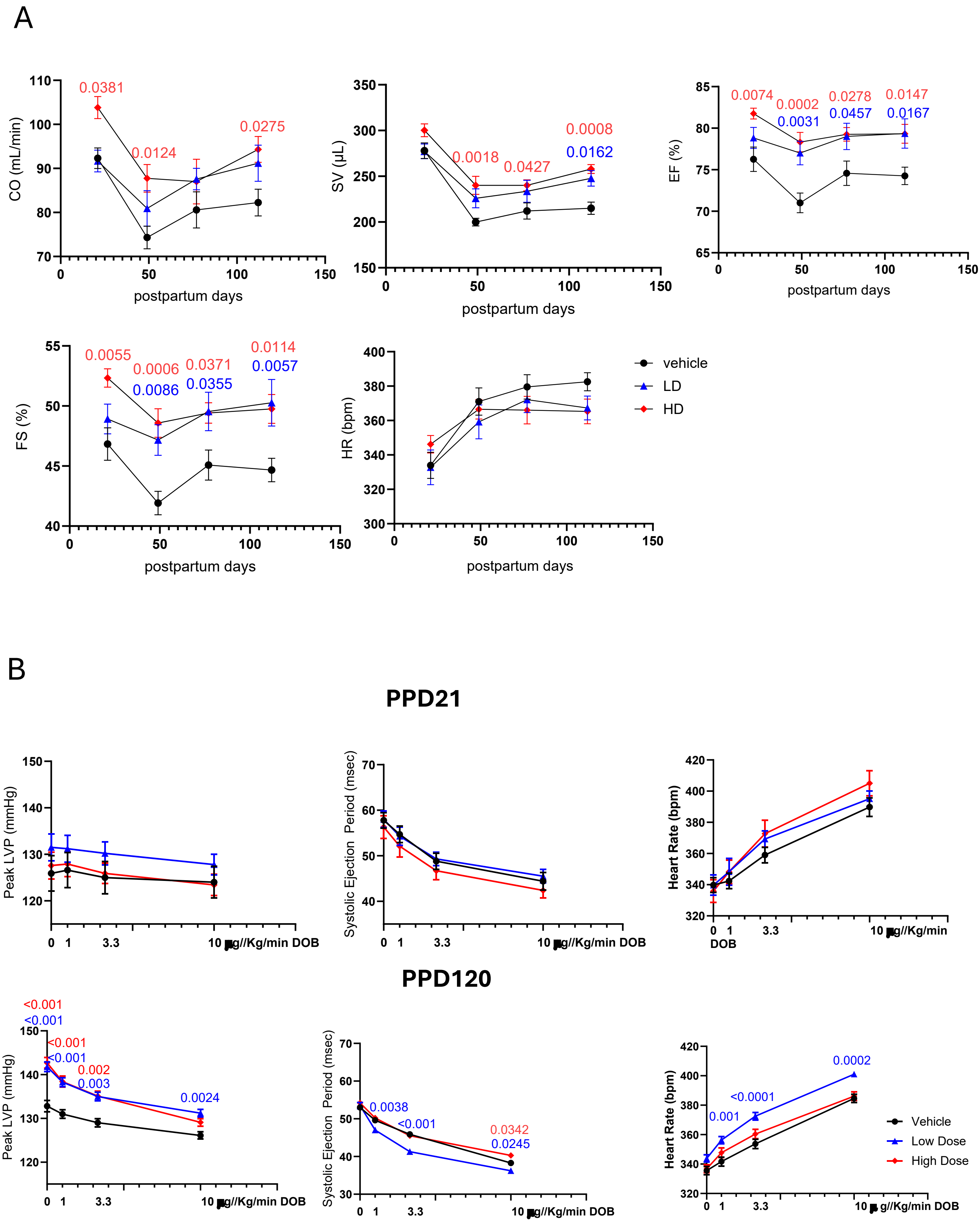
A
CO (mL/min)
postpartum days
0.0381
0.0124
0.0275
SV (µL)
0.0018
0.0427
0.0008
0.0162
EF (%)
0.0074
0.0002
0.0031
0.0278
0.0457
0.0147
0.0167
FS (%)
0.0055
0.0006
0.0086
0.0371
0.0355
0.0114
0.0057
HR (bpm)
vehicle
LD
HD
B
PPD21
Peak LVP (mmHg)
Systolic Ejection Period (msec)
Heart Rate (bpm)
10 µg//Kg/min DOB
PPD120
<0.001
<0.001
<0.001
<0.001
0.002
0.003
0.0024
0.0038
<0.001
0.0342
0.0245
0.0002
0.001
<0.0001
Vehicle
Low Dose
High Dose

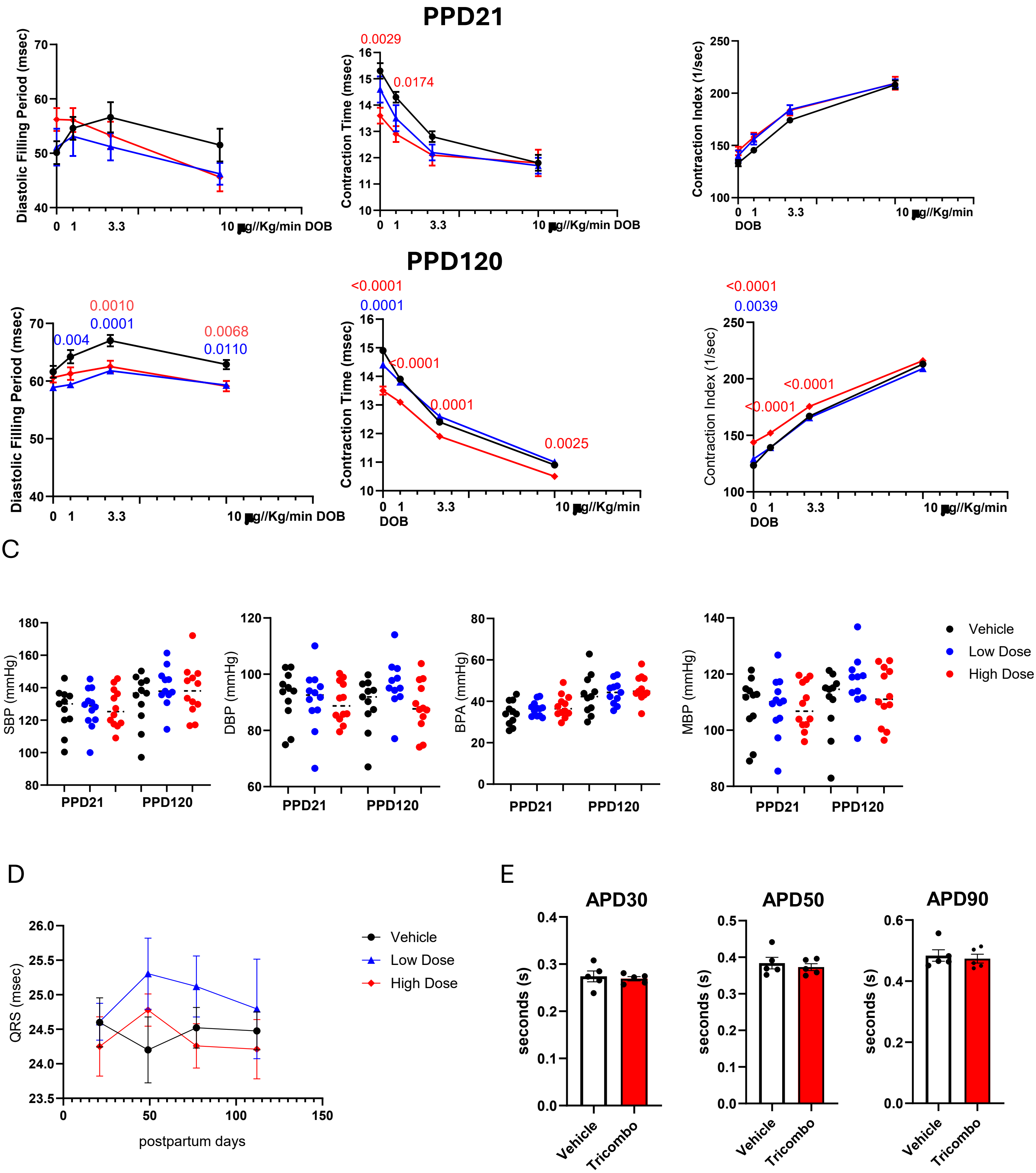

Supplementary Figure 2. Cardiac parameters evaluated in dams administered ABC/DTG/3TC. (A) Cardiac parameters shown as a function of time where repeated measures on the same animals evaluated cardiac output (CO), stroke volume (SV), ejection fraction (EF), fractional shortening and heart rate (HR), (FS) (n=12 rats/group). Analysis performed via two-way ANOVA with multiple comparisons, p-values shown are corresponding group vs. vehicle control. (B) Dobutamine challenge results at both postpartum day 21 (PPD21) and postpartum day 120 (PPD120) (n= 12 rats/group). Increasing doses of dobutamine were infused per minute, which are depicted in the figure. Analysis performed via two-way ANOVA with multiple comparisons, p-values shown are corresponding group vs. vehicle control. (C) Blood pressure measurements at PPD21 and PPD120 (n=12 rats/group). (D) QRS interval as measured by ECG (n=12 rats/group). (E) Action potential duration (APD) at 30%, 50% and 90% as measured via multi-electrode array in hIPSC-CMs exposed to ABC/DTG/3TC (n=6 wells/group). Statistical significance established via t-test.

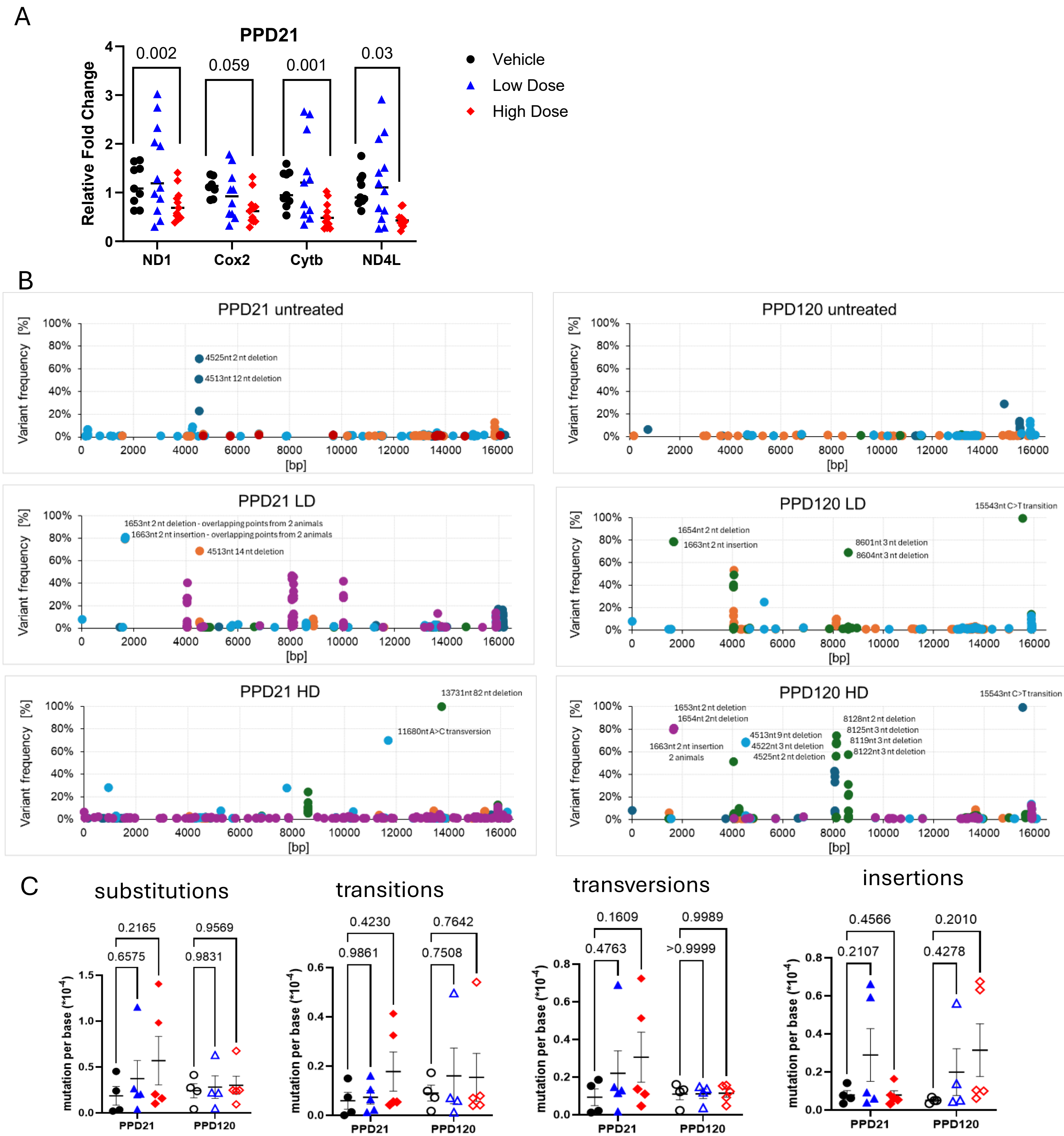

Supplementary Figure 3. Mitochondrial DNA measurements (A) Mitochondrial DNA genes amplified at PPD21 and PPD120 via qPCR, including *ND1, COX2, CYTB, ND4L* and *18S* (n=12 rats/group). (B) Detailed information on variant frequency and deletions and mutations from mitochondrial DNA of dams from PPD21 and PPD120 in all dose groups (n=4-5 rats/group). (C) Mutations shown as substitutions, transitions, transversions and insertions (n=5 rats/group). Analyzed via one-way ANOVA with multipole comparisons.

A

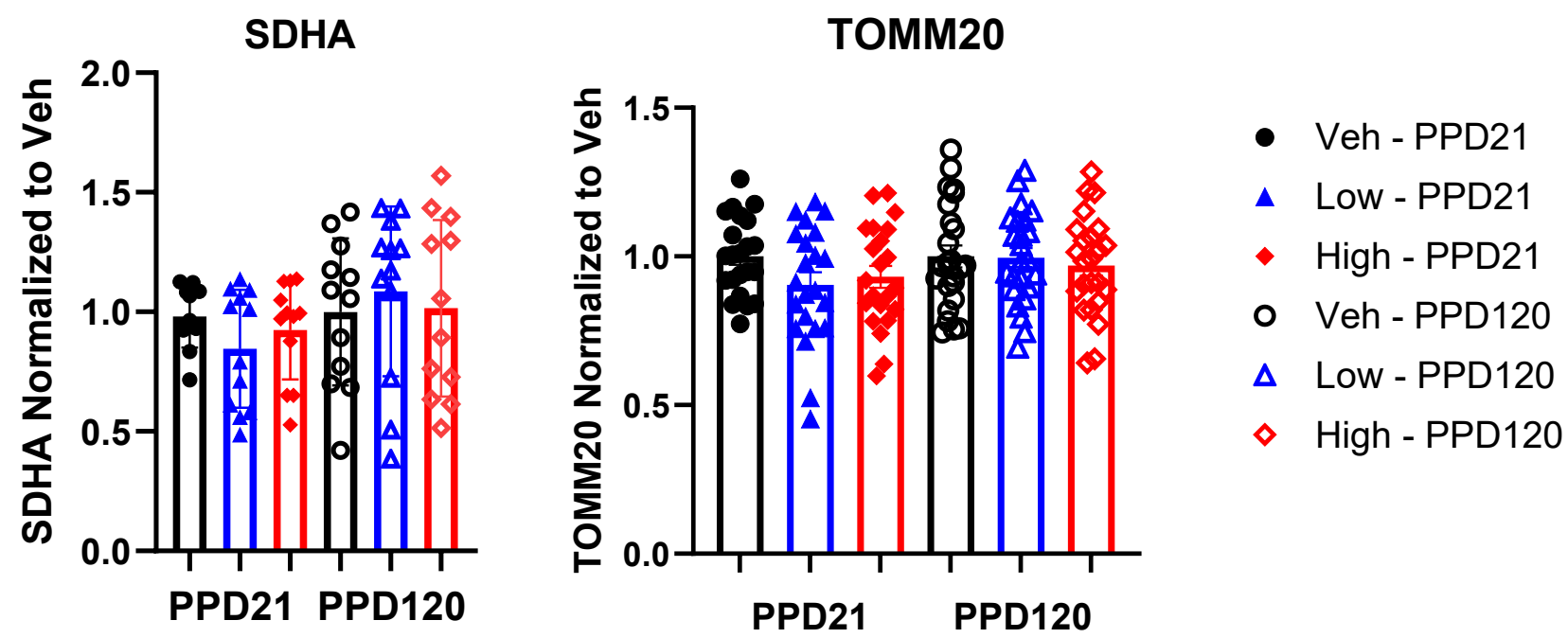
SDHA
SDHA Normalized to Veh
2.0
1.5
1.0
0.5
0.0
PPD21
PPD120
TOMM20
TOMM20 Normalized to Veh
1.5
1.0
0.5
0.0
PPD21
PPD120
Veh - PPD21
Low - PPD21
High - PPD21
Veh - PPD120
Low - PPD120
High - PPD120

B

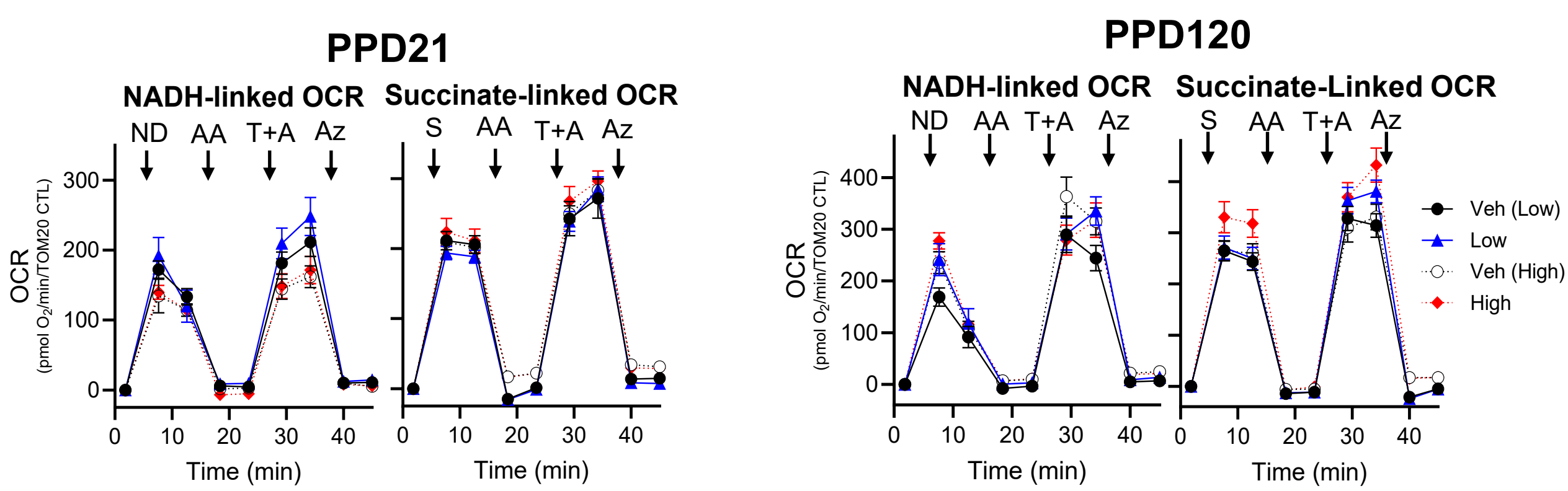
PPD21
NADH-linked OCR
Succinate-linked OCR
ND
AA
T+A
Az
S
AA
T+A
Az
OCR
(pmol O2/min/TOM20 CTL)
300
200
100
0
0
10
20
30
40
Time (min)
Time (min)
PPD120
NADH-linked OCR
Succinate-Linked OCR
ND
AA
T+A
Az
S
AA
T+A
Az
OCR
(pmol O2/min/TOM20 CTL)
400
300
200
100
0
Time (min)
Time (min)
Veh (Low)
Low
Veh (High)
High

C

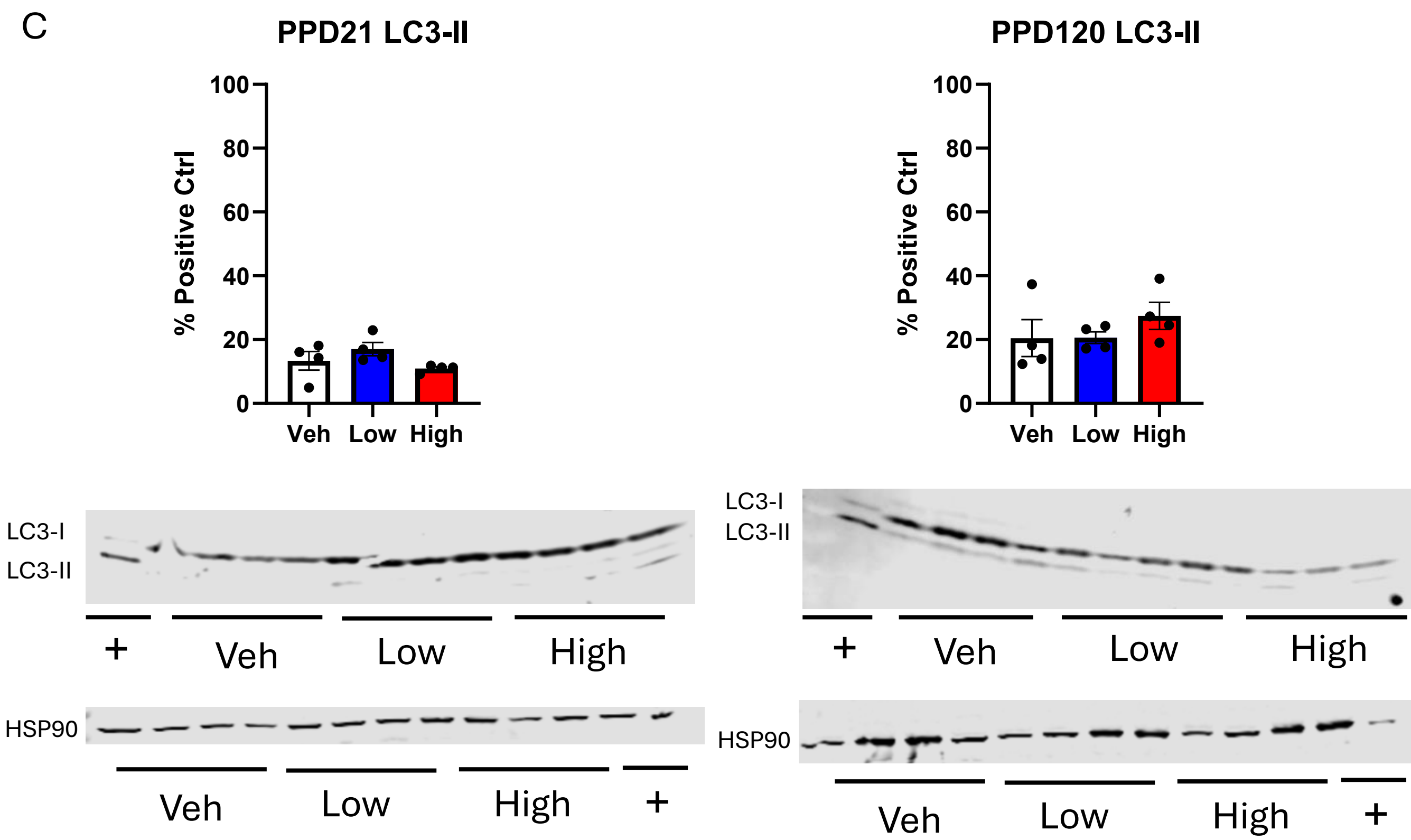
PPD21 LC3-II
% Positive Ctrl
100
80
60
40
20
0
Veh
Low
High
PPD120 LC3-II
% Positive Ctrl
Veh
Low
High
LC3-I
LC3-II
+
Veh
Low
High
HSP90
Veh
Low
High
+
LC3-I
LC3-II
+
Veh
Low
High
HSP90
Veh
Low
High
+

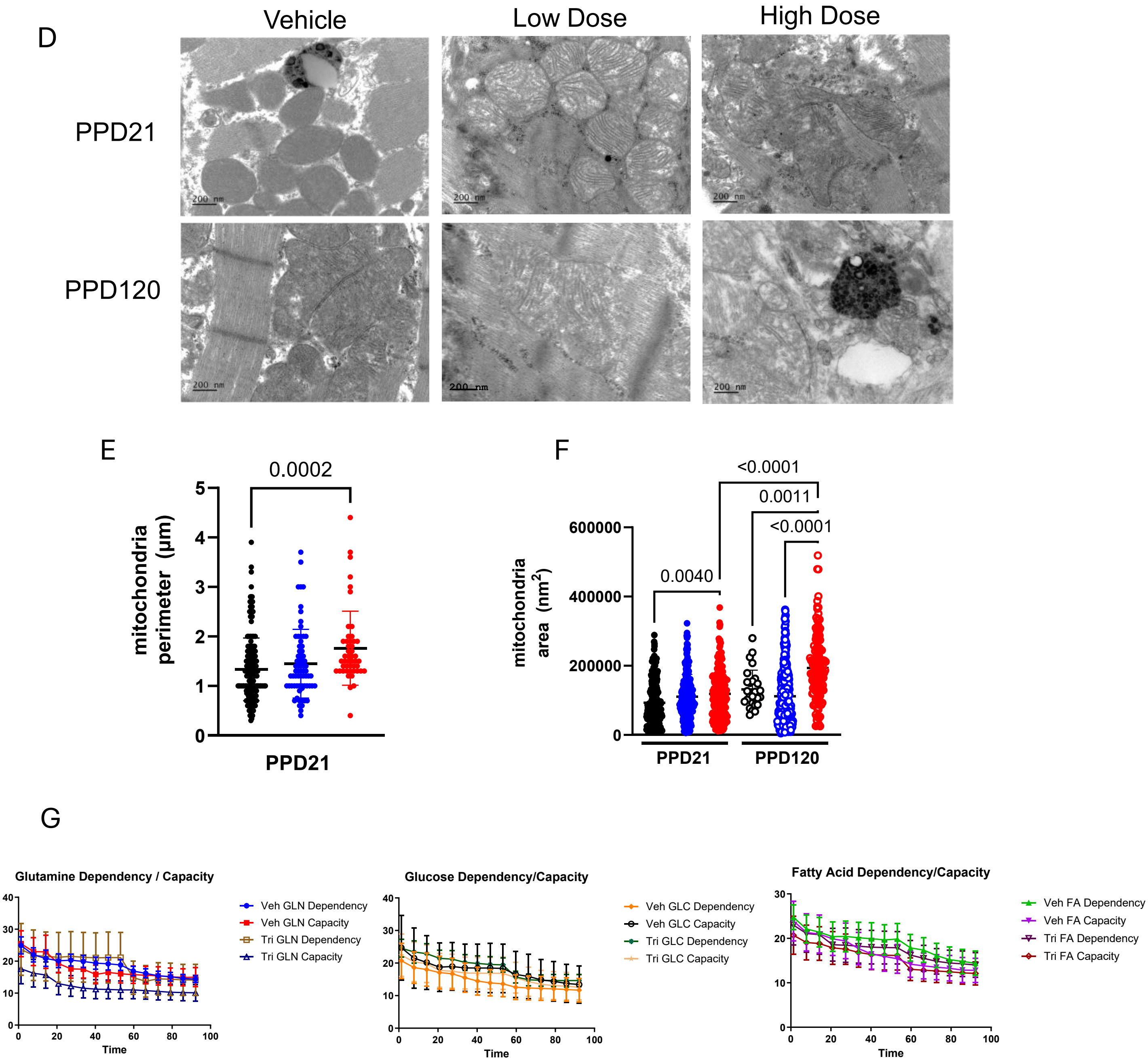

Supplementary Figure 4. Additional mitochondrial measurements for rats and hiPSC cardiomyocytes administered ABC/DTG/3TC. (A) SDHA and TOMM20 protein levels in the hearts of rats exposed to low and high dose ABC/DTG/3TC measured by SimpleWestern Jess at PPD21 and PPD120 (n=12 hearts/group) and normalized to total protein. (B) Oxygen consumption rate of hearts of dams at PPD21 and PPD120 using frozen tissue measured within timepoint (n=6 rats/group). (C) SDS-Page of LC3 and quantification of LC3-II to determine presence of autophagy in hearts (n=4 dams/group). Lipidized LC3 (LC3-II) was normalized to total LC3 for each sample, and these were normalized to positive control. Positive control for this assay is cell lysate from HEK293 dosed with 10µM FCCP for 24 hours to induce autophagy. Housekeeping was established via probe of HSP90 in a separate membrane with the same samples. Repeated twice for continuity. Analyzed via one-way ANOVA with multiple comparisons. (D) Electron microscopy images in heart tissue of rats exposed to low and high dose ABC/DTG/3TC; data are representative of n=2/dose/timepoint with 2 independent blocks analyzed per animal. About 100 images were taken/block. (E) Mitochondrial perimeter and (F) area as measured by AI visualization and segmentation. (G) Mitochondrial respiration tracings in iPSC cardiomyocytes exposed to either DMSO 0.02% or ABC/DTG/3TC for 7 days (n=6/group) using SeaHorse Flux Analyzer and the Agilent Fuel Flex Assay (n=8 wells/group).

A

Gene Name	Organism	F1 Sequence	R1 Sequence
18S	Rat	TCAACTTTCGATGGTAGTCGCC	TTGGATGTGGTAGCCGTTTCT
ND1	Rat	TTCCTAGGCCCCTTATATCACA	ATCGAAAACGGGGGTAGGATG
ND4L	Rat	TGCGAAGCAGCAGTAGGTTTA	TTTGTACGTAGTCTGTTCCGT
CYTB	Rat	CCTCCCATTCATTATCGCCG CCCTTGC	GTCTGGGTCTCCTAGTAGGTC TGGGAA
COX2	Rat	ATCCGAAGACGTCCTCGACT	ACTGTAGCTTGGTTTAGGCGG
ND3	Rat	CCCATATGAATGTGCCTTCGAC	TTG AAA AAG GAA GGC GTG CAG
7S-A	Rat	GAGGATGGTAGAAATAGAGA CC	
7S-B1	Rat	CCCCAAGCATATAAGCATGT AATA	
7S-B2	Rat	ACCATCAACACCCAAAGCTG	
18S	Human	CGGACAGGATTGACAGATT	CCAGAGTCTCGTCGTTATCG
ND1	Human	CGATTCCGCTACGACCAACT	AGGTTTGAGGGGGAATGCTG
ND4L	Human	ATCGCTCACACCTCATATCCT	GGAGTGGGTGTTGAGGGTTAT

B

primer 1	FWD	AAAGTGTGCTTG*G*A	1007-1020	
primer 2	REW	ACAAGTGATTAT*G*C	2027-2040	
primer 3	FWD	ACATCAAGTCTT*T*C	3061-3074	
primer 4	REW	ATATTTTGTTGC*T*G	4067-4080	
primer 5	FWD	TGGAAGGATTCA*A*A	5087-5100	
primer 6	REW	ATATCCGAAGGG*T*T	6121-6174	
primer 7	FWD	TCATCAGCTC*C*C	7105-7116	
primer 8	REW	GAAGGTTGGTT*G*A	8161-8173	
primer 9	FWD	GACGGAATTTA*C*G	9168-9180	
primer 10	REW	TGGGAGGAGTA*T*G	10188-10200	
primer 11	FWD	TTGATAGCAACA*T*G	11221-11234	
primer 12	REW	ATTGCTTGGA*G*G	12241-12252	
primer 13	FWD	ATTGCCCTAGA*A*C	13232 -13244	
primer 14	REW	CCTGTGAGGA*T*T	14269 – 14280	
primer 15	FWD	TACTCTGGTCT*T*G	15301-15313	
primer 16	REW	GGTGTATGTGG*A*A	16300-16313	
		* phosphorothioate bonds		

Supplementary Figure 5. qPCR primer sequences used for mitochondrial DNA copy number, 7S DNA and sequencing. (A) Primer sequences used for qPCR including 7S DNA. (B) Primer sequences used to determine deletions and mutations in mitochondrial DNA.

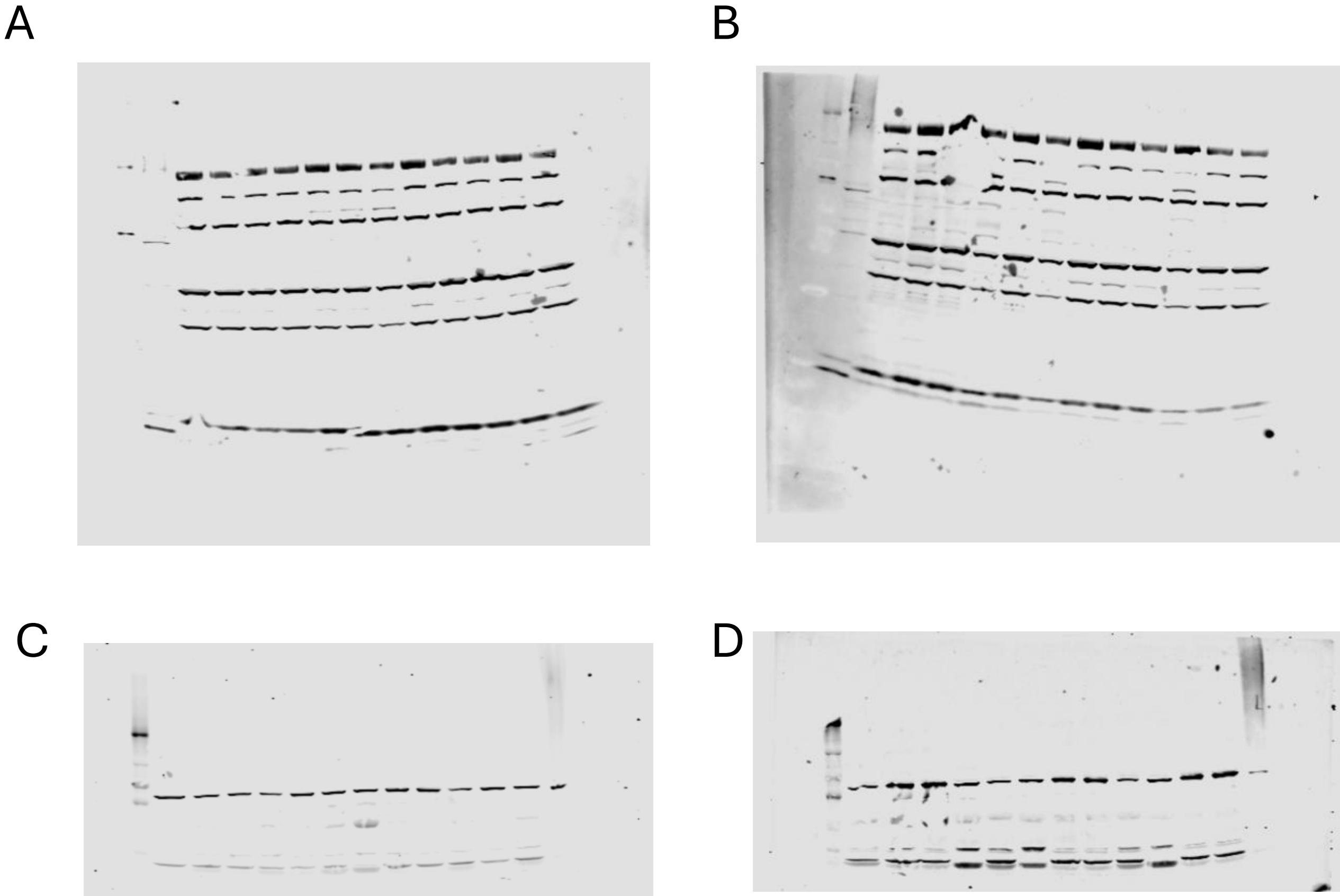

Supplementary Figure 6. Uncropped blots from Supplementary Figure 4C. (A) LC-3 blot from hearts from PPD21 dams and (B) LC-3 blot from hearts from PPD120 dams. (C) HSP90 blot from hearts from PPD21 as loading control and (D) HSP90 blot from hearts from PPD120 as loading control.

Computer data acquisition systems used

Computer System	Manufacturer	Data Type
InTempConnect	Onset Computer Corporation	Temperature Monitoring (refrigerators and freezers)
Elemental Machines	Elemental Machines	Environmental Storage
EMCS	Siemens	Animal Facility Environment
NTP Provantis	Instem	Animal Toxicology and Organ Weights
IOX2	EMKA Systems	Hemodynamic
Easy Matrix	EMKA Technologies	Electrocardiographic
Vevo 3100 Imaging Systems	FUJIFILM VisualSonics Corporation	Echocardiographic
Provantis[a]	Instem	Clinical Pathology

a. Data exported from testing facility Provantis and imported into NTP Provantis was used for clinical pathology summary data reporting and was maintained electronically by the NTP. The raw data collected in testing facility Provantis was retained and archived per GLP regulatory requirements.

1.5 STATISTICS

Appropriate animal numerical data were analyzed using statistical tests available through NTP Provantis. All appropriate quantitative in-life data collected at the testing facility using the Provantis system were analyzed for test article effects by parametric or nonparametric analysis of variance (ANOVA). For all data, normality was determined by the Shapiro-Wilks test and homogeneity of variances was determined by Levene's test. Data may have been log-transformed to meet parametric assumptions. For parametric data determined to be normally distributed and homogenous among groups, an ANOVA F-test was used to determine whether there were differences among the group means. If the ANOVA F-test was significant, then tests for differences between the vehicle group and each of the comparison groups were conducted using Dunnett's test, which adjusts for multiple comparisons. For nonparametric data that were not normally-distributed and/or non-homogenous, a Kruskal Wallis test was used to determine whether there are differences among the group means. If the Kruskal-Wallis test was significant, then tests for differences between the vehicle group and each of the comparison groups were conducted using Wilcoxon tests and the Bonferroni-Holm method to correct for multiple comparisons. Statistical tests for normality and homogeneity were performed at the 0.05 level of significance ($p < 0.05$). All other statistical tests were performed at the 0.001, 0.01, and 0.5 levels of significance after accounting for multiple comparisons, where indicated.

The occurrence of clinical observations was not analyzed statistically but were reported as an incidence of a given finding by group and sex.

The cardiovascular data are assumed to be normally distributed and a one-way ANOVA with repeat measurement was used. See Appendix G for the description of the statistical approaches used.

1.6 SUMMARY

The design of the study is summarized below. A copy of the protocol outline is included in Appendix K, a copy of the study protocol and protocol amendments, and a summary of relevant deviations are included in Appendix L.

Summary of experimental design, materials, and methods

**Animals and Animal Maintenance**	
Strain/Species	Sprague Dawley (HSD:Sprague Dawley®SD®) rats
Receipt Date	Shipment A: 20 May 2021<br>Shipment B: 27 May 2021<br>Shipment C: 03 Jun 2021<br>Shipment D: 10 June 2021<br>Shipment E: 17 Jun 2021<br>Shipment F: 24 Jun 2021
Animal Source	Envigo (Indianapolis, IN)
Age at Receipt	Listed in the order of shipment A to shipment F:<br>97 days (13 weeks); Date of Birth = 12 Feb 2021<br>97 days (13 weeks); Date of Birth = 19 Feb 2021<br>97 days (13 weeks); Date of Birth = 26 Feb 2021<br>97 days (13 weeks); Date of Birth = 05 Mar 2021<br>97 days (13 weeks); Date of Birth = 12 Mar 2021<br>97 days (13 weeks); Date of Birth = 19 Mar 2021
Quarantine Period	Shipment A: 20 May 2021 to 21 Jun 2021<br>Shipment B: 27 May 2021 to 21 Jun 2021<br>Shipment C: 03 Jun 2021 to 21 Jun 2021<br>Shipment D: 10 Jun 2021 to 16 Jul 2021<br>Shipment E: 17 Jun 2021 to 16 Jul 2021<br>Shipment F: 24 Jun 2021 to 15 Jul 2021
Health Evaluations	Listed in the order of shipment A to shipment F:<br>Veterinary exams: 21 May 2021 and 18 Jun 2021<br>Veterinary exams: 27 May 2021 and 18 Jun 2021<br>Veterinary exams: 04 Jun 2021 and 18 Jun 2021<br>Veterinary exams: 10 Jun 2021 and 16 Jul 2021<br>Veterinary exams: 18 Jun 2021 and 16 Jul 2021<br>Veterinary exams: 25 Jun 2021 and 13 Jul 2021<br><br><u>Quarantine Disease Screening (Non-mated females)</u>:<br>24 May 2021 (shipment A) and 14 Jun 2021 (shipment D), 10 females; Opti-Spot, fur swab, and fecal exams. No significant findings.

Summary of experimental design, materials, and methods (continued)

Animals and Animal Maintenance	
Health Evaluations (continued)	4-Week Disease Screening (Non-mated females): 21 Jun 2021 (shipment A) and 12 Jul 2021 (shipment D), 10 females; Opti-Spot, fur swab, fecal exams, and gross necropsy. No significant findings.<br>Unscheduled Disease Screening, Core Cohort (F_1 animal): 18 Aug 2021; 1 female sentinel animal, fur swab, fecal exam, and gross necropsy. No significant findings.<br>Terminal Disease Screening, Core Cohort (F_1 Animals): 10 Nov 2021; sentinel animals, 5 male/4 females; Opti-Spot, fur swab, fecal exams, and gross necropsy. No significant findings.<br>Terminal Disease Screening, 12-Month Cohort (F_1 Animals): 13 Jul 2022; randomly selected animals from the control group, 5/sex; Opti-Spot, fur swab, and fecal exams, and gross necropsy. No significant findings.
Method of Animal Distribution	Date of Dam randomization:<br>Shipment A: 22 May 2021<br>Shipment B: 30 May 2021<br>Shipment C: 06 Jun 2021<br>Shipment D: 13 Jun 2021<br>Shipment E: 25 Jun 2021<br>Shipment F: 27 Jun 2021<br><br>On GD 4 F_0 females were assigned to one of three concentration groups (0, 150/12.5/75, or 300/25/150 mg/kg) using NTP Provantis software (Instem, Stone, UK), which assigned animals to study groups by body weight stratification so that the group mean weights at randomization were similar.<br><br><ins>PND 4 Biological Sampling and F_1 Litters:</ins> The number of pregnant dams was insufficient to fill the required biological sampling requirements, at the directive of the NIEHS Project Officer the dams and pups listed in Table 10 were utilized for PND 4 collections. All other litters were standardized on each litter's respective PND 4 to a maximum of 8 pups (4/sex) using NTP Provantis (selections were random). When fewer pups of a sex are available, the remaining positions were filled with the opposite sex. All culled pups from control dams were also utilized for PND 4 biological sample collections. Any litter that had less than 8 total pups were not reduced.<br><br><ins>F_0 Dams and F_1 Animals:</ins> F_0 Dams were randomized to Cohorts (Interim and Core) and their pups were

Summary of experimental design, materials, and methods (continued)

Animals and Animal Maintenance	
Method of Animal Distribution (continued)	distributed to the same Cohorts. (Interim, Core) using Microsoft Excel with all remaining pups being distributed to the 12 Month Cohort.<br>Date of randomization<br>F0 Dams and F1 Animals:<br>Shipment A: 23 Jun 2021<br>Shipment B: 29 Jun 2021<br>Shipment C: 06 Jul 2021<br>Shipment D: 08 Jul 2021<br>Shipment E: 15 Jul 2021<br>Shipment F: 22 Jul 2021
Animal Identification	F_0 Dams: Indelible tail mark and cage card (upon arrival)<br>F_1 Pups: Appendage tattoo (PND 0)<br>F_0 Dams and F_1 Animals: Tail tattoo and cage card (PND 25-28) (see DR-T06055-AMP-0007)
Age at First Dose (Day 0)	F_0 Dams:<br>Subset A: 102 Day (GD 6)<br>Subset B: 102 Day (GD 6)<br>Subset C: 102 Day (GD 6)<br>Subset D: 102 Day (GD 6)<br>Subset E: 102 Day (GD 6)<br>Subset F: 102 Day (GD 6)
Weight at First Dose (Day 0)	F_0 Dams:<br>Subset A: 210.1 g to 253.4 g<br>Subset B: 227.5 g to 257.4 g<br>Subset C: 222.4 g to 254.5 g<br>Subset D: 222.3 g to 268.2 g<br>Subset E: 217.9 g to 265.1 g<br>Subset F: 222.4 g to 265.8 g
Method of Termination	CO_2 inhalation: F_0 dams not delivered<br>Decapitation: fetuses and pups through PND 10, PND 4 biological sampling animals<br>CO_2/O_2 or isoflurane prior to exsanguination: Interim, Core, and 12-Month Cohort animals

Summary of experimental design, materials, and methods (continued)

Animals and Animal Maintenance	
Date(s) of Scheduled Termination	F$_0$ Dams:<br>PND 4 Removals: 6/14/21, 6/21/21, 6/28/21, 7/19/21<br>No Delivery Removals: 6/14/21, 6/21/21, 6/28/21, 7/5/21, 7/12/21, 7/19/21<br>Interim Dam Removals: 7/1/21, 7/8/21, 7/15/21, 7/21/21, 7/29/21, 8/5/21<br>Core Dam: 10/6/21, 10/13/21, 10/20/21, 10/27/21, 11/3/21, 11/10/21
Age at Scheduled Termination	F$_0$ Dams:<br>PND 4 Removals: 122 days<br>No Delivery Removals: 122 days<br>Interim Removals: 138-139 days<br>Core Removals: 236 days
Feed	Irradiated NIH-07 and NTP-2000 wafer feed manufactured by Zeigler Brothers (Gardners, PA), available *ad libitum*. NIH-07 feed was provided from receipt until completion of the perinatal phase. NTP-2000 feed was provided following weaning.<br>All feed lots were analyzed for contaminants by Neutron Products, Inc. (Dickerson, MD). Results were reviewed by the Attending Veterinarian and are included in the study file. Results were acceptable for use.
Feeders	Stainless-steel feeders (Lab Products, Inc., Seaford, DE), integrated into cage units, changed at least weekly.
Maximum Storage Time for Feed	180 days post-milling
Storage Conditions for Feed	JM10-1-701:<br>Temperature ≤ 72ºF; relative humidity ≤ 59%

Summary of experimental design, materials, and methods (continued)

Animals and Animal Maintenance	
Water	Water from the Village of West Jefferson water supply was provided on an *ad libitum* basis via the automatic watering system (Edstrom Industries, Waterford, WI), sanitized at least every 2 weeks (see DR-T06055-AMP-0022 for exceptions).<br><br>Once during the study, a sample collected from the animals' drinking water source was submitted to Belmont Labs (Englewood, OH) and analyzed for organic, inorganic, and bacterial contaminants. Results were reviewed by the Attending Veterinarian and are included in the study file. Results were within acceptable limits.
Animals per Cage	Dams: Individually housed; housed with litters once delivered; group housed (up to 3/cage) after weaning
Cages	22"L x 12.5"W x 8"H polycarbonate cages, changed and sanitized at least once weekly through PND 4, and at least twice weekly thereafter (Lab Products, Inc, Seaford, DE).<br><br>Animals were assigned to cages sequentially by individual animal study number and then assigned to vertical columns within like concentration groups using Microsoft Excel. Columns were randomly assigned to positions on the study racks. Cage rotation occurred at each rack change (at least every 2 weeks) in accordance with testing facility SOP (AMPNTOX.V-05).
Bedding	Irradiated hardwood bedding (Sani-Chips, P.J. Murphy Forest Products Corporation, Montville, NJ), changed with cages. Bedding was monitored for microbial analysis; reports provided by Envigo (Madison, WI) Analytical reports were reviewed by the Attending Veterinarian. Results were within acceptable limits.

Summary of experimental design, materials, and methods (continued)

Animals and Animal Maintenance	
Enrichment	Irradiated natural crinkled kraft paper (Crink-l'nest™, The Andersons, Maumee, Ohio) was used in the cages of dams upon receipt and dams with litters. On GD 19, natural crinkled kraft paper was removed from the cages of pregnant dams until PND 4. Analytical reports for the natural crinkled kraft paper were received and monitored by the Testing Facility.<br><br>Red rectangular polycarbonate shelters (Rat Retreats™, Bio-Serv, Flemington, NJ) were placed in each cage of the non-mated females and all group housed rats upon weaning. Enrichment items were replaced at each cage change according to SOP AMPCOR.V-020.
Racks	Stainless steel (Lab Products, Inc., Seaford, DE), changed, sanitized, and rotated at least every 2 weeks
Rack Filters	Spun-bonded polyester (National Filter Media Corporation, Olive Branch, MS) changed (with racks) at least every 2 weeks.
Cage and Feeder Washer	LYNX Model 536 LXI Tunnel Washer (LYNX Product Group, Wilson, NY)
Rack Washer	LYNX Model 430LX Cage and Rack Washer (LYNX Product Group, Wilson, NY)
Equipment Washing Compounds	Cage-Klenz 180, LpH ag, and Pro Klenz Two (STERIS Corporation, St. Louis, MO)
Pest Control	Conducted according to the Integrated Pest Management (IPM) program specifically created for the testing facility by an outside consultant. Structural and mechanical means of pest control were used along with high standards of facility sanitation and housekeeping. No compounds were used that would have interfered with the conduct of this study.
Room Sanitization	Prior to housing animals, the study room was disinfected in accordance with SOP AMPARF.III-009.
Animal Room Environment	Specified temperature range 69 to 75°F<br>Specified relative humidity range 35 to 65%<br><br>JM10-1-514:<br>Actual temperature range 71 to 73°F; 1392/1392 (100%) of readings within range.<br>Actual relative humidity range 46 to 72%: 1386/1392 (99.6%) of readings within range.<br><br>JM10-1-516:<br>Actual temperature range 70 to 73°F; 4200/4200 (100%) of readings within range.<br>Actual relative humidity range 44 to 75%: 4191/4200 (99.8%) of readings within range.

Summary of experimental design, materials, and methods (continued)

Animals and Animal Maintenance	
Animal Room Environment	JM10-1-755:<br>Actual temperature range 71 to 73°F; 1392/1392 (100%) of readings within range.<br>Actual relative humidity range 46 to 72%: 1386/1392 (99.6%) of readings within range.
Room Air Filters	Air changes and pressurized readings were monitored at least twice yearly by an outside consultant to ensure environmental controls provided a minimum of 10 fresh air changed per hour. Reports were reviewed by the Attending Veterinarian and are included in the study file. Fresh air passed through a bank of pre- and post-filters prior to being supplied to the study room.
Room Lighting	A 12 hours on/12 hours off fluorescent light cycle was controlled electronically. The light intensity was checked during the rat quarantine confirming light intensity of 27 to 33 foot-candles 3.3 feet from the floor (SOP ARF.III-010). Operation of the timer controlling the light cycle was verified three times per week, in the evening and in the morning, while the room was occupied with animals.
**Experimental Design**	
Concentration Groups	0, 150/12.5/75, and 300/25/150 mg/kg ABC/DTG/3TC
Size of Study Groups	<u>F_0 Dams (Perinatal Phase):</u><br>0 mg/kg – 34 females<br>150/12.5/75 mg/kg – 33 females<br>300/25/150 mg/kg – 33 females<br><br><u>F_0 Dams (Interim Cohort):</u><br>0 mg/kg – 11 females<br>150/12.5/75 mg/kg – 12 females<br>300/25/150 mg/kg – 12 females<br><br><u>F_0 Dams (Core Cohort):</u><br>12 females/group

Summary of experimental design, materials, and methods (continued)

Experimental Design	
Size of Study Groups (continued)	Sentinels:<br>5 males/5 females
Duration of Dose Administration/Exposure	F_0 Dams: Once daily administration from GD 6 through PND 21, and continuing up to 13 weeks post-weaning<br><br>F_1 Cohorts: Pups received the test article during *in utero* exposure and up to 3 weeks via the dam's milk.
Type and Frequency of Observations	Rats were observed 2 times per day for mortality/moribundity, with at least 6 hours between, 7 days per week, except on the day of receipt when they were observed once.<br><br>Perinatal Phase Dams:<br>Individual body weights were recorded on arrival, on GD 4 (for randomization), 6, 9, 12, 15, 18, and 21. Body weights for surviving littered dams were recorded on PND 1, 4, 7, 10, 13, 16, 19, and 21, some interim dams were removed from study and all of their collections ended on PND 20. Parturition checks were performed twice-daily beginning on GD 21 until pups were delivered or through GD 24, whichever came first. During gestation, food consumption measurements began with the exposure of dosed feed on GD 6 and were recorded on GD 9, 12, 15, 18, and 21. During lactation, food consumption measurements per cage began on PND 1 and were recorded on PND 4, 7, 10, 13, 16, 19, and 21 (please see DEV-004083). During gestation, formal clinical observations were recorded daily from GD 6 through delivery. During lactation, formal clinical observations were recorded daily from PND 1 through PND 21.<br><br>Pups:<br>Birth examinations were conducted on PND 0 and viability, sex, and live/dead count were recorded for each litter daily beginning on PND 0. Litter weights (live total and live by sex) were recorded on PND 1. Individual body weights were recorded on PND 1, 4, 7, 10, and 13.<br><br>Beginning on PND 14 (following randomization), NTP Provantis pup collections were completed using the F_1 animal study identification numbers for individual

Summary of experimental design, materials, and methods (continued)

Experimental Design	
Type and Frequency of Observations (continued)	clinical observations (PND 14-21 included in this report) and individual body weights (PND 16-21 available in NTP Provantis but will not be included in this report). Pups remained with dam during these collections and pups appendage tattoos were used to identify the animals which correlated to the assignment of F_1 animal study identification numbers.<br><br>Core F_0 Dam and F_1 Animal Cohorts:<br>For all F_0 dams, clinical observations for signs of toxicity, body weights, and food consumption measurements were recorded on PND 22, weekly thereafter, and at removal from study (see DR-T06055-AMP-0009, DR-T06055-AMP-0020 and DR-T06055-AMP-0032 for exceptions).<br><br>_______________
PND 4 Biological Sampling	On PND 4, dams (with litters) and culled pups from surviving dams were randomly selected for biological sampling as indicated in Table 10 (see DR-T06055-AMP-0013 and DR-T06055-AMP-0015 Selected dams were gavage dosed on the day of collection. Blood and tissue collection occurred approximately 2 hours after dosing and all samples on a given day were collected within a 2-hour period.<br><br>Whole blood (up to 2 mL) was collected from the aortic trunk (via decapitation) of dams into tubes containing K_3EDTA. Two aliquots of whole blood (125 µL each) were collected, and the remaining blood was processed to plasma (up to 5 aliquots of 125 µL per animal).<br><br>Whole blood (as much as possible) was collected from pups from the aortic trunk (via decapitation) into tubes containing K_3EDTA and processed to plasma (up to 5 aliquots of 125 µL per pup). This included all culled pups in the control group, all pups in the control litters

Summary of experimental design, materials, and methods (continued)

Experimental Design	
PND 4 Biological Sampling (continued)	selected for biological sampling, and 5 pups/sex/litter selected from the test article 150/12.5/75 and 300/25/150 mg/kg groups. Blood was kept on wet ice until plasma isolation was completed (within 60 minutes of collection) using a refrigerated centrifuge set at a target of approximately 4°C for at least 10 minutes at a relative centrifugal force of at least 1800g. Sample were flash frozen in liquid nitrogen within 2 hours of collection and stored in a freezer set to maintain -85 to -60°C until shipment.<br><br>Following blood collection, whole hearts were collected and weighed as one sample per animal (see DR-T06055-AMP-0001). Hearts were flash frozen in liquid nitrogen and stored in a freezer set to maintain -85 to -60°C. All remaining tissues, including the carcass, were discarded.<br>All plasma and heart samples were shipped frozen on dry ice to an NTP-designated laboratory for analysis and reporting.
Cardiovascular and Hemodynamic Assessments	Electrocardiography (ECG):<br>All animals were lightly anesthetized with isoflurane to effect (~3%) via induction chamber and then maintained with ~1.5 to 3% isoflurane with a cone mask for the conduct of cardiovascular data collection. Both ECG and ECHO were collected in the same session.<br><br>Three electrodes were attached to animals for lead II ECG monitoring and recorded on a computer using the IOX Data Acquisition system (EMKA Technologies) and the following parameters were recorded and assessed: PQ interval (msec), QRS interval (msec), QT interval (msec), and QTcV (QT corrected for heart rate using Van de Water's formula) (msec).<br><br>Echocardiography (ECHO):<br>Following stabilization under anesthesia, left ventricular (LV) function and dimensions were monitored and recorded using a Vevo 3100 ultrasound machine with a 25 to 55MHz probe (FUJIFILM VisualSonics Corporation, Canada). Transthoracic two-dimensional (2D) B-mode, as well as 2D-guided M-mode, echocardiography was obtained from the long- and short-axis views at the level of papillary muscles. A simultaneous single-lead (e.g., lead II) electrocardiogram

Summary of experimental design, materials, and methods (continued)

Experimental Design	
Cardiovascular and Hemodynamic Assessments (continued)	(ECG) was obtained and recorded for the determination of cardiac cycle intervals during echo evaluation with the echo images and cine loops. The recorded images and cine loops were exported and analyzed offline.<br><br>The following ECHO parameters were recorded and assessed: heart rate (beats per minute [bpm]), systolic diameter (mm), diastolic diameter (mm), systolic volume (µL), diastolic volume (µL), stroke volume (µL), ejection fraction (%), fractional shortening (%),cardiac output (mL/min), left ventricular (LV) mass (mg), LV mass corrected (mg), systolic LV anterior wall thickness (mm), diastolic LV anterior wall thickness (mm), systolic LV posterior wall thickness (mm), and diastolic LV posterior wall thickness (mm).<br><br>Hemodynamic Assessment (HEMO):<br>For the hemodynamic assessments, anesthesia was induced by isoflurane (up to 5%) via a gas chamber and maintained via face mask with 95% O_2/5% CO_2. Following anesthesia induction, the animal was shaved for vascular access and positioned in dorsal recumbency on a heated surgery table. Infiltration anesthesia at the incision site(s) (i.e., femoral triangle, jugular furrow, or ventral cervical region) was done with 2% lidocaine (~0.05 mL diluted with 0.9% NaCl) administered prior to the instrumentations. A tracheostomy was performed for ventilatory support, and the animal was ventilated (~100 breaths/min, ~2.5 mL tidal volume with 95% O_2/5% CO_2) with an adjustable small animal ventilator (Harvard Apparatus). A suitable vein (left or right jugular or femoral vein) was isolated and cannulated for the administration of dobutamine.<br><br>Transthoracic needle electrodes forming a single-lead ECG (e.g., lead II) were placed. To record arterial pressures, a high-fidelity micromanometer catheter was placed via a cut down and inserted into a femoral artery or carotid artery and advanced towards the aorta. To record LV pressures, the right carotid artery was isolated, dissected free from the surrounding tissue, and cannulated with a high-fidelity micromanometer catheter. This catheter was advanced retrograde across the aortic valve and into the LV chamber.

Summary of experimental design, materials, and methods (continued)

Experimental Design	
Cardiovascular and Hemodynamic Assessments (continued)	A vein (jugular or femoral vein) was isolated and cannulated with an appropriately sized IV catheter or polyethylene (PE) tubing for drug administrations, as necessary. Following a baseline/hemodynamic stabilization period (up to 20 minutes), the overall cardiovascular responses were monitored for up to 120 minutes following baseline data collection.<br><br>Dobutamine challenges were administered by IV infusion for F_0 dams or by intraperitoneal (IP) injection f For the F_0 animals, dobutamine was delivered in a dose escalation pattern at 0, 1, 3.33, and 10 µg/kg by IV infusion targeting 5-7 minutes at each dose. Hemodynamic recovery was allowed (up to 10 minutes) between each challenge. For the F_1 pups (4 weeks only), dobutamine was delivered in a dose escalating pattern at 0, 0.5, 1, and 2 mg/kg IP injections. Hemodynamic recovery was allowed (up to 10 minutes) between each challenge. All animals were euthanized at the end of the experiment while they were under a surgical anesthetic plane.<br><br>The following parameters were recorded and assessed for the hemodynamics-left ventricle: pressure at end diastole (EDP), peak LV pressure (PeakLVP), systolic ejection period (SEP), diastolic filling period (DFP), contraction time (CT), relaxation time (RT), maximal rate of pressure change during systole (dP/dt+), maximal rate of pressure change during diastole ((dP/dt-), contractility index, dP/dtmax divided by instantaneous pressure (CI), time-constant of LV relaxation (Tau), heart rate (HR). The following parameters were recorded and assessed for the hemodynamics-systemic blood pressure: systolic, diastolic, mean arterial blood pressure and pulse pressure.<br><br>See DR-T06055-AMP-0018 for hemodynamic collection exceptions.

Summary of experimental design, materials, and methods (continued)

Experimental Design	
Cardiovascular and Hemodynamic Assessments (continued)	Core Cohort F_0 Dams<br>Noninvasive survival cardiovascular ECG and ECHO assessments were performed on all surviving Core F_0 dams (before PND 21), and at 4 and 8 weeks after weaning. Terminal cardiovascular ECG, ECHO, and hemodynamic assessments were performed on all surviving Core F_0 dams 13 weeks after weaning.
Cardiac Biomarkers	Changes in cardiac health as monitored by cardiac troponins C, I and T (cTnC, cTnI and cTnT), and Atrial Natriuretic Peptide (ANP). Blood collection occurred on PND 117 or 118 and processed to serum for analysis. Serum concentrations of cTnC, cTnI and cTnT were quantified by immunoassay using commercially available immunoassay kits (BEST Center, Battelle).
Blood Sample Collection	Interim Cohort F_0 Dams:<br>Whole blood was collected from Interim Cohort F_0 dams on PND 17 (survival, via jugular vein, approximately 2 hours after dosing) and PND 19-22 (terminal, via catheter placed during cardiovascular assessment) into tubes containing K_3EDTA and processed to plasma (2 aliquots of 125 µL per animal on PND 17 and up to 5 aliquots of 125 µL per animal on PND 19-22) (see DR-T06055-AMP-0030 for exceptions). Blood was kept on wet ice until plasma isolation was completed (within 60 minutes of collection) using a refrigerated centrifuge set at a target of approximately 4°C for at least 10 minutes at a relative centrifugal force of at least 1800g. Samples were flash frozen in liquid nitrogen within 2 hours of collection and stored in a freezer set to maintain -85 to -60°C until shipped.<br><br>All plasma samples were shipped to an NTP-designated laboratory for analysis. Results of the analyses are not included in this report.<br><br>Core Cohort F_0 Dams:<br>Following terminal cardiovascular assessment (13 weeks post weaning), whole blood (up to 10 mL) was collected from all surviving F_0 dams (via catheter placed during cardiovascular assessment while anesthetized with

Summary of experimental design, materials, and methods (continued)

Experimental Design	
	isoflurane) into a serum separator tube and processed to serum (up to 5 aliquots of 1 mL per animal). Blood was kept on wet ice until serum isolation was completed (within 60 minutes of collection) using a centrifuge set at room temperature for at least 10 minutes at a relative centrifugal force of at least 1800g. Samples were flash frozen in liquid nitrogen within 2 hours of collection and stored in a freezer set to maintain -85 to -60°C.<br><br>One aliquot, designated for serum chemistry evaluation, was analyzed for total cholesterol, LDL, HDL, and triglycerides). One aliquot was submitted to the NTP Frozen Tissue Bank. The remaining aliquots were shipped to the BEST Center for analysis of the following cardiac biomarkers: cTroponin (cTnC, cTnI, and cTnT).<br><br>____________________<br><br>All serum samples were shipped to an NTP-designated laboratory for analysis. Results of the analyses are not included in this report.

Summary of experimental design, materials, and methods (continued)

Blood Sample Collection (continued)	All serum samples were shipped to an NTP-designated laboratory for analysis. Results of the analyses are not included in this report.
Necropsy and Histopathological Evaluation	Perinatal Phase:<br>A gross necropsy was performed on all dams that were euthanized on GD 26 (for females that did not deliver) or at scheduled humane termination on PND 4 (see DR-T06055-AMP-0008 and DR-T06055-AMP-0031 for exceptions). Females that did not deliver were humanely terminated by asphyxiation using 100% CO_2 and PND 4 dams and pups were humanely terminated by decapitation. Water and feed were provided up until termination. Tissues were collected at scheduled terminations on PND 4 (see PND 4 Biological Sampling section). The diaphragm from 2 culled pups/sex/litter and gross lesions from all culled pups were collected and fixed in 10% neutral buffered formalin (NBF), trimmed, processed, embedded, sectioned, and stained with hematoxylin and eosin for microscopic evaluation. Carcasses were discarded following collections. Results of the analyses are not included in this report.<br><br>Interim Cohort F_0 Dams<br>A gross necropsy was performed on all interim dams and pups at scheduled necropsy. Water and feed were provided up until termination. Following terminal cardiovascular procedures or blood collection, interim animals were humanely terminated via exsanguination.<br><br>Immediately following euthanasia, the heart was removed from each animal, weighed, rinsed with phosphate buffered saline (PBS) and sectioned (see DR-T06055-AMP-0034 for exceptions). The heart was bisected along its longitudinal axis parallel to the interventricular septum (IVS), with the IVS retained with the right side of the heart. The apex of the heart was removed from the left side and flash frozen. The remainder of the left side was flash frozen. The left apex and remaining left side were collected individually and stored in a freezer set to maintain -85 to -60°C until shipped. The right side was emersed in NBF for

Summary of experimental design, materials, and methods (continued)

	Experimental Design
Necropsy and Histopathological Evaluation (continued)	24 hours, after which the heart was removed, and a thin slice of the right ventricular free wall was transferred to glutaraldehyde and processed for ultrastructural analysis. The remainder of the right side of the heart was returned to NBF until fully fixed and processed for histopathology. Sections included portions of the atria, ventricles, segments of the atrioventricular valves and the origin of the aorta (when present).<br><br>All tissue samples were shipped (hearts shipped frozen and blocks were shipped at ambient temperature to the to the NTP Frozen Tissue Bank for analysis. Results of the analyses are not included in this report.<br><br>Core Cohort F_0 Dams :<br>A gross necropsy was performed on all core dams a that were humanely terminated at an unscheduled interval or at scheduled necropsy. The<br>F_0 dam that died at an unscheduled interval was humanely terminated by exsanguination. Following terminal cardiovascular procedures or blood collection, interim animals were humanely terminated via exsanguination (see DR-T06055-AMP-0019 for exceptions). Water and feed were provided up until termination.<br><br>Immediately following euthanasia, the heart, brain, liver, and kidney were collected as follows. The heart was collected, weighed, rinsed with PBS, and sectioned as described for Interim Cohort animals above. The brain was collected and separated by region and frozen separately by hemisphere. Two samples (approximately 250 mg total) were collected from the left liver lobe and the right kidney and cut into approximate 5 mm^3 pieces and aliquoted into two screw cap tubes. All tissues were flash frozen and stored in a freezer set to maintain -85 to -60°C until shipped. All frozen specimens were shipped on dry ice to the NTP Frozen Tissue Bank. Any further analyses were not provided to the study director and are not included in this report.<br><br>All collected tissues (listed in Table 11) were preserved in 10% neutral buffered formalin (NBF), trimmed, embedded, sectioned, and stained with hematoxylin and

Summary of experimental design, materials, and methods (continued)

	Experimental Design
	eosin. Histopathological evaluation of the following tissues was performed on all animals: gross lesions, heart, left kidney, liver, lung, mesenteric roll, and diaphragm with central tendon. Carcasses were discarded following collections. Results of the analyses of the Histopathology are included in this report.<br><br>___________________<br><br>All frozen tissues were flash frozen and stored in a freezer set to maintain -85 to -60°C until shipped. All frozen specimens were shipped on dry ice to the NTP Frozen Tissue Bank. Any further analyses were not provided to the study director and are not included in this report.<br><br>12-Month Histopathology Cohort (and Early Death

Summary of experimental design, materials, and methods (continued)

Experimental Design	
Necropsy and Histopathological Evaluation (continued)	u<br>Organ weights were recorded for brain, epididymides, heart, kidneys, liver, lungs, spleen, and thymus. Histopathological evaluation of the following tissues was performed on all animals: brain [seven sections including (1) olfactory bulbs, (2) frontal-parietal cortex including basal ganglia, (3) mid-parietal cortex and thalamus,<br>(4) mid-brain with substantia nigra and red nucleus, (5) posterior colliculi, (6) mid-cerebellum including cranial nerve VIII, and (7) posterior medulla]; diaphragm with central tendon, epididymides; esophagus; eyes; femur [including diaphysis with marrow cavity and epiphysis (femoral condyle with epiphyseal cartilage plate, articular cartilage and articular surface)]; gross lesions; heart and aorta; kidneys; large intestine (cecum and colon); small intestine (duodenum, jejunum, and ileum); liver (two sections including left lateral lobe and median lobe); lungs and mainstem bronchi; lymph nodes (mandibular and mesenteric); mammary gland with adjacent (inguinal) skin; spleen; stomach (forestomach and glandular); thigh muscle; thymus; and thyroid gland.
**HIV Tricombo Therapy Formulation**	
Formulation Preparation	Formulations of HIV Tricombo Therapy (ABC/DTG/3TC) in aqueous 0.2% methylcellulose/0.1% Tween 80 were prepared at concentrations of 150/12.5/75 and 300/25/150 mg/kg by the Sponsor's analytical chemistry contract laboratory and shipped, ready to use, to the testing facility.
Storage Conditions	Room temperature
