# Supplementary material for: Antiretroviral therapy in the peripartum period impairs post pregnancy cardiac reverse remodeling in a rodent model
Source: Nat Commun. 2026 Jul 17;17:8765. doi: 10.1038/s41467-026-75510-x (PMC13494002; doi:10.1038/s41467-026-75510-x)
Supplement: Supplementary file 2 [file 41467_2026_75510_MOESM2_ESM.pdf]

Title: Supplementary Data 1.

Description: mtDNA sequencing results. Mitochondrial DNA (mtDNA) sequencing data as measured via qPCR (n=5 rats/group/timepoint).

Title: Supplementary Data 2.

Description: Cardiac RNA-sequencing results. Gene list of all differentially expressed genes (DEGs) with different comparisons including low dose PPD21 vs low dose PPD120, vehicle PPD21 vs vehicle PPD120, high dose PPD21 vs high dose PPD120, high dose PPD21 vs vehicle PPD21 and high dose PPD120 vs vehicle PPD120. Statistical significance was determined by DESeq2 with a Benjamini Hochberg multiple testing correction (n=6 rats/group/timepoint, FDR < 0.05).

Title: Supplementary Data 3.

Description: Cardiac Metabolomics. All significant and non-significant metabolites in the heart at all doses at PPD21 and PPD120 and all comparisons. Differential metabolite abundance was assessed using ANOVA on log-transformed metabolomics data, and fold changes were calculated relative to the control group; metabolites with an adjusted significance level (FDR < 0.05) are shown.

Supplementary Data 4. Liver RNA-sequencing results.

Description: Liver RNA-sequencing results. Gene list of all differentially expressed genes measured by high dose PPD120 vs vehicle PPD120 and low dose vs vehicle PPD120. Statistical significance was determined by DESeq2 with a Benjamini Hochberg multiple testing correction (n=6 rats/group/timepoint, FDR < 0.05).
