# Supplementary material for: Antiretroviral therapy in the peripartum period impairs post pregnancy cardiac reverse remodeling in a rodent model
Source: Nat Commun. 2026 Jul 17;17:8765. doi: 10.1038/s41467-026-75510-x (PMC13494002; doi:10.1038/s41467-026-75510-x)
Supplement: Supplementary file 3 [file 41467_2026_75510_MOESM4_ESM.pdf]

nature portfolio

Corresponding author(s): Janine Hertzog Santos

Last updated by author(s): June 22, 2026

Reporting Summary

Nature Portfolio wishes to improve the reproducibility of the work that we publish. This form provides structure for consistency and transparency in reporting. For further information on Nature Portfolio policies, see our Editorial Policies and the Editorial Policy Checklist.

Statistics

For all statistical analyses, confirm that the following items are present in the figure legend, table legend, main text, or Methods section.

n/a	Confirmed	
☐	☒	The exact sample size (*n*) for each experimental group/condition, given as a discrete number and unit of measurement
☐	☒	A statement on whether measurements were taken from distinct samples or whether the same sample was measured repeatedly
☐	☒	The statistical test(s) used AND whether they are one- or two-sided<br>*Only common tests should be described solely by name; describe more complex techniques in the Methods section.*
☒	☐	A description of all covariates tested
☐	☒	A description of any assumptions or corrections, such as tests of normality and adjustment for multiple comparisons
☐	☒	A full description of the statistical parameters including central tendency (e.g. means) or other basic estimates (e.g. regression coefficient) AND variation (e.g. standard deviation) or associated estimates of uncertainty (e.g. confidence intervals)
☐	☒	For null hypothesis testing, the test statistic (e.g. *F*, *t*, *r*) with confidence intervals, effect sizes, degrees of freedom and *P* value noted<br>*Give P values as exact values whenever suitable.*
☒	☐	For Bayesian analysis, information on the choice of priors and Markov chain Monte Carlo settings
☒	☐	For hierarchical and complex designs, identification of the appropriate level for tests and full reporting of outcomes
☒	☐	Estimates of effect sizes (e.g. Cohen's *d*, Pearson's *r*), indicating how they were calculated

Our web collection on statistics for biologists contains articles on many of the points above.

Software and code

Policy information about availability of computer code

Data collection	Measurement of electron microscopy images of mitochondria were generated using Visiopharm, a commercially available AI based image analysis software platform.
Data analysis	The following commercial programs were used to perform data and statistical analyses:<br>- Microsoft Excel 365<br>- GraphPad Prism 10.0.1<br>- Rsubread version 2.14 and DESeq2 version 1.42 for transcriptomics analysis<br>- Metabolomics analysis was performed using Metabolon bioinformatics pipeline

For manuscripts utilizing custom algorithms or software that are central to the research but not yet described in published literature, software must be made available to editors and reviewers. We strongly encourage code deposition in a community repository (e.g. GitHub). See the Nature Portfolio guidelines for submitting code & software for further information.

Data

Policy information about availability of data

All manuscripts must include a data availability statement. This statement should provide the following information, where applicable:

- Accession codes, unique identifiers, or web links for publicly available datasets
- A description of any restrictions on data availability
- For clinical datasets or third party data, please ensure that the statement adheres to our policy

The data generated in this study are provided in Supplementary Information/Source Data files. Metabolomics data is available in Supplemental Data 3. RNA-seq genomics data are deposited at the GEO and can be accessed through GSE325635 [https://www.ncbi.nlm.nih.gov/geo/query/acc.cgi?acc=GSE325635], mtDNA sequencing data is available through the SRA accession number PRJNA1480161 [PRJNA1480161 - SRA - NCBI].

Research involving human participants, their data, or biological material

Policy information about studies with human participants or human data. See also policy information about sex, gender (identity/presentation), and sexual orientation and race, ethnicity and racism.

Reporting on sex and gender	This study considered sex in study design as this study focuses on pregnancy and the postpartum period. Due to study design, we only utilized female rats (dams) for this study and all study findings.
Reporting on race, ethnicity, or other socially relevant groupings	Not applicable
Population characteristics	No human data
Recruitment	No participants / recruitment needed
Ethics oversight	All animal work was performed at a third party contractor (Battelle)

Note that full information on the approval of the study protocol must also be provided in the manuscript.

Field-specific reporting

Please select the one below that is the best fit for your research. If you are not sure, read the appropriate sections before making your selection.

☒ Life sciences ☐ Behavioural & social sciences ☐ Ecological, evolutionary & environmental sciences

For a reference copy of the document with all sections, see nature.com/documents/nr-reporting-summary-flat.pdf

Life sciences study design

All studies must disclose on these points even when the disclosure is negative.

Sample size	Sample size was determined based on prior toxicokinetic studies performed at NIEHS for ABC/DTG/3TC and power analysis to determine an n=12 animals per group.
Data exclusions	Data was excluded from analyses based on Grubbs outlier analysis on an experiment by experiment basis - no large amounts of data were excluded from analysis.
Replication	Experiments were all performed at least two independent times on different days. No attempts at replication failed.
Randomization	Sample allocation was random. Additionally, rats were randomized into control vs treated groups upon arrival.
Blinding	Sample collection was not blinded to study group, and cardiac assessment was also not blinded to study group. Biochemical analyses were not blinded because experiments were performed and analyzed by the same researchers; genomics and metabolomics data were analyzed by either a bioinformatician or contracted vendor that had no prior knowledge about the samples other than their labels. Electron microscopy images were analyzed utilizing AI by investigators that had no prior knowledge about samples other than labels.

Reporting for specific materials, systems and methods

We require information from authors about some types of materials, experimental systems and methods used in many studies. Here, indicate whether each material, system or method listed is relevant to your study. If you are not sure if a list item applies to your research, read the appropriate section before selecting a response.

Materials & experimental systems

n/a	Involved in the study	
	x	Antibodies
	x	Eukaryotic cell lines
x		Palaeontology and archaeology
	x	Animals and other organisms
x		Clinical data
x		Dual use research of concern
x		Plants

Methods

n/a	Involved in the study	
x		ChIP-seq
x		Flow cytometry
x		MRI-based neuroimaging

Antibodies

Antibodies used	NDUFS1 (Cell Signaling, Cat.060153S, 1:1000, Rabbit mAb), SDHA (Cell Signaling, Cat. 5839S, 1:1000, Rabbit mAb), TOMM20 (Novus, NBP1-81556, 1:50), anti-rabbit secondary IR antibody (Biotechne, 043-820, 1:15,000), anti-MAP1LC3A (Millipore Sigma, L8918, 1:1000), HSP90 (Invitrogen, PA5-27410, 1:1000), goat-anti rabbit IRDye 680RD (LI-COR, 925-681817, 1:15,000)
Validation	Antibodies are validated rigorously by companies. Cell Signaling extensively validates their antibodies and that information can be found here: https://www.cellsignal.com/about-us/our-approach-process/antibody-validation-western-blotting?srsltid=AfmBOoq3VFGQAs5MQRk51pOhiZbwJOTLoKwEBluckF342YL9PhE6mKWs. Novus also validates antibodies and that information can be found here: https://www.novusbio.com/reproducibility.html?srsltid=AfmBOopOQ8wpFc5EGKHRO9_2AAVXpIW8pvBfvQM-vjZpjduhgHaSLFQZ. The same for biotechne: https://www.bio-techne.com/reagents/antibodies/antibody-validation. Additionally, all antibodies are regularly used in our lab and place bands at the expected molecular weight.

Eukaryotic cell lines

Policy information about cell lines and Sex and Gender in Research

Cell line source(s)	Cell experiments were performed in Fujifilm iCell Cardiomyocyte2. Cells were purchased from FujiFilm Biosciences (Cat. C1058) and from a female donor.
Authentication	Cells were authenticated and tested by Fujifilm before purchase by SNP genotyping, and viability and sterility were also tested. Additionally, functionality was tested via field potential duration and beat rate.
Mycoplasma contamination	Cells were tested for mycoplasma by Fujifilm and only cultured for 7 days at a time - no evidence of contamination was present through the study. Additionally, cells were tested for mycoplasma before acquisition by PCR.
Commonly misidentified lines (See ICLAC register)	No commonly misidentified cell lines were used.

Animals and other research organisms

Policy information about studies involving animals; ARRIVE guidelines recommended for reporting animal research, and Sex and Gender in Research

Laboratory animals	Female Sprague-Dawley rats aged 11-14 weeks were used for this study. Animals were sourced from Envigo (Indianapolis, IN). Genetic background was wild type, and no knockouts were used for this study.
Wild animals	No wild animals were used
Reporting on sex	These findings apply to only one sex. We only investigated females for this study as we are focused on changes to the heart during pregnancy and the postpartum period.
Field-collected samples	No field collected samples
Ethics oversight	Protocol for this study was approved internally at NIEHS, and all animal ethical protocols were approved through Battelle where all animal research was conducted (ACUC protocol T06055). All animal studies were conducted in an animal facility accredited by AAALAC International.

Note that full information on the approval of the study protocol must also be provided in the manuscript.

Plants

Seed stocks	No seed stocks
Novel plant genotypes	No plants used in this study
Authentication	No plants used in this study
