# Supplementary material for: Antiretroviral therapy in the peripartum period impairs post pregnancy cardiac reverse remodeling in a rodent model
Source: Nat Commun. 2026 Jul 17;17:8765. doi: 10.1038/s41467-026-75510-x (PMC13494002; doi:10.1038/s41467-026-75510-x)
Supplement: Supplementary file 4 [file 41467_2026_75510_MOESM5_ESM.pdf]

nature portfolio

Peer Review File

Antiretroviral therapy in the peripartum period impairs post pregnancy cardiac reverse remodeling in a rodent model

Corresponding Author: Dr Janine Santos

This file contains all reviewer reports in order by version, followed by all author rebuttals in order by version.

Version 0:

Reviewer comments:

Reviewer #1

(Remarks to the Author)
In the manuscript by Taube et al, the authors explore the functional impact of antiretroviral therapeutics (ARTs) on the physiological remodleing of the rat heart that manifests following pregnancy. Pregnancy is known to induce physiological hypertrophy and cardiac remodeling that is, under normal conditions, a fully reversalable process. The authors propose, based on cited litterature, that ARTs could negatively impact this reverse remodeling (RR) via mitochondrial DNA since ARTs have been proposed to have off-target effects on mitochondrial DNA replication. In this study, the authors perform physiological characterization of rats treated with low or high dose (LD or HD) ARTs from DG6 to PPD120 and investigate cellular and molecular remodeling using endpoint RNAseq, Metabolomics, and molecular biology and biochemistry analyses. Some mechanisms are explored in parallel in iPSC-derived human cardiomyocytes. The authors prpose that ARTs modulate RR and are associated with heart failure with preserved ejection fraction (HfpEF) and mitochondrial dysfunction. While this is outside my field of expertise, it appears that a number of studies have explore the impact of ARTs (e.g. AZT) on cardiac health in rat and mouse models since the invention of ARTs, with insights into mitochondrial structure and functon(PMID: 14522417, 9895221, 26248377). In my opinioin, the study addresses a very important open question regarding the impact of ARTs on cardiac health in the context of pregnancy. I am unaware whether this is the first study to explore mitochondria and cardiomyopathy by ARTs during pregnancy-induced reverse remodeling in rats, and if so this study has tackled a novel question in biology and medicine.
The overall design of the study is solid, especially the detailed cardiac phenotyping in vivo and in vitro, which allowed them to observe subtle differences induced by ART using very large samples sizes. The metabolomic phenotyping is also impressive and informative and it is very important to know that the LD and HD can be quantified in situ and tracks as expected. Unfortunately, the quantitative characterization of mitochondrial function/dysfunction in this study is rather limited and primarily restricted to qPCR measurements of mtDNA levels and enzymatic measurements based on frozen material that provides limited insights into how ARTs might functionally impact mitochondrial bioenergetics at the molecular level. RNAseq was performed to monitor changes in mitochondrial gene expression and this is helpful to support the notion that mtDNA dysfunction impacts mtRNAs. However, I would caution against the overinterpretation of transcriptional levels of metabolic and OXPHOS genes as a proxy for functional enzymatic changes, since the degree of correlation between transcriptomes, proteomes, and metabolic activities in the rodent heart is known to be variable and limited (PMID: 37276142, 36493852). Beyond these limitations, the remaining conclusions are fair and well-argued, and the data are not overinterpreted. Nevertheless, I have a number of outstanding questions and confusions raised throughout the study that require further clarification and explanation and several areas in which additional experimentation is required to substantiate the very important conclusions that were drawn in the current manuscript. I would encourage that quantitative measurements of histological and TEM changes should be provided in place of or in addition to the pathology report provided in the supplementals, which alone are insufficient to support the conclusions in the manuscript. The discussion part of the manuscript is very well-written and well-balanced.

Major concerns
1. The major substrate for energy metabolism in the adult heart is fatty acids, and it is important to assess respiration using fatty acids and not carbohydrate substrates, particularly given the intriguing fatty acid measurements reported in Figure 5. Substrate preference changes during cardiac hypertrophy and its metabolic inflexibility in this regard could underscore defects in reverse remodeling. The authors acknowledge this important possibility in lines 483-485 of the discussion but do not directly test it.
2. Of course, altered lipid profiles could reflect modified bioenergetic substrate usage (as the authors propose) or lipotoxicity

leading to myocyte death. Neither are mutually exclusive and both are functionally different and important. Neither has been functionally evaluated and both together, or individually, could contribute impaired reverse remodeling. Measurement of TUNEL staining of cardiac histology or serum cardiac troponin levels can be used to provide insights into myocyte death.
3. I am highly skeptical of the ability to conclude on mitochondrial respiration (rather than specific enzymatic activities of OXPHOS) using frozen rather than fresh biopsies. While enzymatic assays of I-III-IV and II-III-IV have been performed on frozen material for decades, these assess the specific activities of enzymes and not mitochondrial respiration. I am aware that protocols (cited by the authors) have been published on this topic, but the limits of this system do not allow one to assess the coupling of mitochondria, which is critical for cardiac function in vivo. In the absence of pregnancy, ARTs have been proposed to trigger mitochondrial toxicity through various mechanisms that can have direct (cristae) or indirect impacts on respiration and coupling therefore, making such studies highly relevant for functional insights into reverse remodeling that is (according to the authors fig Supplementary 3D), independent of mitochondrial content.
4. Given the literature and proposed mechanisms of ARTs on mtDNA (including reference 16 provided by the authors), I would have expected mtDNA mutations and deletions to accompany the depletion of mtDNA. It would be valuable if the authors could use alternative approaches to explore mtDNA deletions and mutational load triggered by long-term ART because it is difficult to imagine that the modest depletion of mtDNA content alone reported in Figure 3 has any meaningful impact on respiratory chain activities reported in the study. A clear indication of the fold changes should be reported in the manuscript.
5. Defects in the integrity or amount of mtDNA would be expected to quantitatively impact that amount of functional OXPHOS complexes, particularly since the authors report changes in CI, CII, and IV activities (Figures 4F, 6R). The only quantitative measurements I could find were of SDHA and TOM20 (by SDS-PAGE?), which showed no differences, but examining one structural subunit out of the >100 proteins of the OXPHOS system is hardly sufficient to exclude effects on OXPHOS assembly/maintenance. Hence, it remains to be demonstrated how or even if (see major concern point 2 above) OXPHOS differences are differentially regulated. Proteomic profiling of these hearts to measure the steady state levels of mitochondrial proteins or BN-PAGE using cardiac mitochondria would provide meaningful insights into this phenomenon.
6. The same care for detailed quantification of in vivo cardiac function and cardiomyocyte function should be applied to the histology and imaging data in a way that is easily visualized in the main or supplementary figures, notably for Figure S3D, given the defects of mitochondrial structure that have been documented in HIV+ and/or hypertrophic hearts. There is a wealth of fascinating and relevant observations in the pathology report that are not quantified in the supplemental data, which limits their appreciation and their value in the manuscript's current form.

Minor concerns.
1. The authors' use of LD and HD ARTs reveals physiological differences that might be attributed to different degrees of cardiac and mitochondrial toxicity. According to the authors, the LD is already 2.4x the abacavir and lamivudine prescribed to humans, while the dolutegravir is 10x the amount. For the HD, these values are 4.8x and 20x respectively (lines 118 to 120). What is the rationale of using such artificially high doses (even the LD, which incurs limited effects throughout the study) if the aim of the study is to provide mechanistic insights into the pathophysiological relevance of ARTs for human health? Is there a biological basis for the use of these elevated doses in rats, perhaps based different susceptibilities and pharmacokinetics between species? If so, it must be more clearly explained and the correct references used for the selection of these doses must be provided.
2. The authors propose that HD induces molecular and functional changes not observed with LD. Indeed, the severity of the cardiac dysfunction is more substantial in HD versus LD rats in Figures 2A, B, C, D, E, F, G, H, and I. However, in some functional tests, the LD procures more dysfunction than the HD relative to vehicle control, as in Figures 2K and 1H, as well as in Supplementary Data 2B and 2D. These observations seem at odds and necessitate explanation.
3. Figure legends need further explanations, particularly Figures 3F, 6R, 4E-I (statistically significant differences must be identified). Statistical tests and significant differences (or lack thereof) must be indicated. In some heat maps, I have the impression that all genes/metabolites are shown while in others, it is only the statistically significantly dysregulated ones. This should be more clearly defined in the figure legends. Figure 6N-P gene labels are missing.
4. References used to support the notion that ARTs drive mitochondrial DNA depletion (references 9, 12, and 16) should be reconsidered and revised or replaced to better substantiate their claims. For example, reference 9 is a review that references two studies (ref 17 and 19), neither of which directly support the claim with experimental evidence relevant to the study
• PMID: 35631648 – this study examined the impact of ARTs on placenta mtDNA, which was not depleted but rather modestly elevated in some conditions. Difficult to conclude since embryonic development was also negatively impacted.
• PMID: 25635584 is itself a review
• Reference 12 is incomplete and I was therefore unable to source the study
• Reference 16 is a much more thorough review – what I gather is that misincorporation nucleotides is more frequent or perhaps more frequently explored than mtDNA depletion, which begs the question about mtDNA mutational load (see major concerns point 3 above)
5. On line 251-260, the authors state that the "half-life of cardiac mitochondria is estabimated to be ~20 days" and hence by PPD21 "~75% of the intial population of mitochondria would have turned over, by PPD120 all that content would have been replaced". This arugment is based on Reference 19 – which is a review with little experimental data to suggest proposed turnover rate and is too simple a view. In fact the 70% figure comes from a referenced paper – (PMID: 20364102) from this review in which HL-1 cells were starved for 3.5h and then mito content was assessed by fluorescence. A more accurate representation of cardiac mitochondrial protein turnover rates in the rodent heart in vivo was performed using heavy water labeling (PMID: 22915825, 26977904) under healthy and diseased conditions, revealing that half-lives of mitochondrial proteins vary dramatically even for proteins belonging to the same macromolecular complex and can be much more or much less than 20days. It remains possible and untested whether mitochondrial protein content is altered in this study.

6. In Figure 6, it is surprising that ALP and ALT levels revealed no obvious liver injury, although liver metabolism and lipids were dysregulated. It is possible that initial liver injury (e.g. PPD20) was present but compensated through hepatocyte

proliferation by PPD120. If ALP and ALT levels cannot be measured at early time points, supporting the absence of livery injury at PPD120 by normal histology would strengthen this claim.

7. Given the impact of ART on body weight, it may be more appropriate to quantify the ration of heart weight/tibia length rather than heart weight/body weight. However, in light of the extensive in vivo echo and ECG studies, this index has relatively little value.

8. I wondered whether the arrhythmia triggers HFpEF or are the defects in cardiac structure causing arrhythmia?

9. There appears to be an error on line 694 (frozen placental lysate).

10. The authors indicate that statistical outliners were identified (line 763) but were these then excluded? If so, on which basis and in which data sets?

11. Error on line 791 for reference 12

Reviewer #2

(Remarks to the Author)
The goal of this study was to examine the impact of a combination therapy of three nucleotide reverse transcriptase inhibitors (NRTIs) on pregnancy-induced changes in cardiac remodeling in the rat. The study shows that NRTIs contributed to impaired pregnancy-associated remodeling and reversal and changes in mitochondrial biology in the hearts and livers of the dam's receiving treatment. Although there is a lot of data provided, significant issues were noted which must be addressed. These are noted as major and minor issues below.

Major Comments
1. One significant major flaw of the present studies is that NRTIs are not examined in the context of pregnancy impacted by HIV. Although rationale is stated suggests that this was done to easily understand the impact of NTRIs on maternal physiology, pregnant women would not take NTRIs during pregnancy without HIV. In addition, without having an HIV group, it is unclear from these studies what the impact of HIV alone is on the examined parameters during pregnancy, and without knowing this it is hard to fully determine how the NRTIs are impacting these parameters. Furthermore, other rodent models have observed that HIV alone does impact many of the key processes examined.
2. Although a substantial amount of data is provided, there is limited mechanistic insight into how NRTIs impact cardiac remodeling and metabolism and many of the key observations remain superficial without in-depth analyses or complete integration, such as the interrogations into the examination of reverse remodeling, diastolic dysfunction, mitochondrial dynamics, and interorgan crosstalk. Because of this, some conclusions are made for which no data is provided, such as the stated differences in substrate utilization and the modest integration of the heart and liver omics datasets to comment on organ cross talk. In addition, changes in glycogen metabolism are stated; however, if TEM samples are not rapidly collected, glycogen can be released and deposited quickly, which reflect an artefact of sample collection rather than a biological change, so glycogen metabolism should be examined further should these conclusions be made.

Minor Comments
1. Line 453 seems to suggest that the treatment regimen tested in the context of these studies is no longer than the ideal treatment regimen for pregnant women with HIV. This should be discussed further because without additional discussion the clinical importance of these studies becomes unclear. In addition, a discussion should be provided as to why NTRIs were started at day 6 of pregnancy in these studies, when women entering pregnancy who are HIV positive may already be on a therapeutic cocktail of treatments, which could have an impact on maternal physiology if they are on these therapies for a longer pre-pregnancy duration.
2. Please include additional information regarding the litters in this study, such as sex distribution and birth weights. It also remains unclear whether the treatment regimens impact on the offspring because minimal interrogation has been performed, which should be discussed.
3. There is a reference in line 490 that is not yet published and should be removed from this; however, given the direct relevance to the present studies, it is unclear why this data is not included here.
4. The title should be changed to accurately match the key observations in the study. At present, based on the data provided, it is unclear whether HFpEF is truly observed.
5. There are a handful of inaccurate statements made regarding the timeline of the reversal of pregnancy-associated remodeling and CVD being the leading cause of maternal mortality that needs to be revised.
6. The manuscript would benefit from additional proofreading to address typographical and grammatical errors, and to reduce the use of non-standard abbreviations and correct gene-related nomenclature. In addition, some of the citations in the reference section are not entered into in the appropriate manner and some of the included figures need to include additional labeling so that it is clear what timepoint is being examined.

Reviewer #3

(Remarks to the Author)
The authors used multiple approaches to rigorously investigate the effects of antiretroviral treatment (ART) containing

nucleoside reverse transcriptase inhibitors (NRTIs) on maternal cardiac function and reverse remodeling in the postpartum period. Given the significant maternal morbidity and mortality related to cardiovascular disease, particularly in pregnant patients living with HIV, and the long-term impacts on cardiovascular (CV) health, this work is timely and significant. I do have several questions and comments about this manuscript, as detailed below. My main concern relates to the authors' framing of the study, particularly the lack of acknowledgment that viremic control through ART (with NRTIs as a core component of most modern ART regimens) is critical in order to reduce risk of vertical transmission to the child and to promote long-term wellbeing of the mother. Based on current evidence, these reductions in harm markedly outweigh the cardiometabolic risks associated with ART, as described in the national guidelines that dictate clinical practice in the United States (Panel on Treatment of HIV During Pregnancy and Prevention of Perinatal Transmission. Recommendations for the Use of Antiretroviral Drugs During Pregnancy and Interventions to Reduce Perinatal HIV Transmission in the United States. Department of Health and Human Services. Available at https://clinicalinfo.hiv.gov/en/guidelines/perinatal). While I realize that the authors may not be clinicians, when examining risks of NRTI-containing ART, it is essential to understand the tremendous benefits of these medications and not to present the risks in isolation. I think that both the Introduction and the Discussion need to be re-framed to reflect this so that these results and their implications can be properly interpreted.

Major Specific Comments:

- The Introduction discusses CV risks during pregnancy and the postpartum period as well as CV risks with long-term antiretroviral use. However, it proposes investigating both the process of reverse remodeling as well as the impact of ART on reverse remodeling. A more thorough discussion about what is currently known about the impact of antiretrovirals on cardiac health could be beneficial here (or in the discussion), and then connected back to how ART can further impact cardiovascular health during the critical period of pregnancy and postpartum. Also, some explanation of the impact of lactation on CVD and how that informs the authors' decision to analyze cardiometabolic health after weaning would be helpful to frame the study design.

- Justification of the selection of the specific ART regimen and dosing studied here, in addition to the time points for assessments and the relationship to lactation (as above), would be helpful towards the beginning of the manuscript. As a clinician who prescribes ART to my pregnant patients, I was confused about the choice of ART regimen as it is not commonly used in current practice.

- More specifics in the Results sections in general would be helpful. The authors tend to describe general trends and then reference figures, without providing numerical results to help convey the effect size and the statistical significance of the findings. In light of this, I did not fully appreciate until the end of the Discussion section that the observed cardiac phenotypes were mild and subclinical. This is an important point and it was not immediately clear from the Results as currently presented.
  - o Example: While line 207 describes the collective findings as indicating of "subclinical features of diastolic dysfunction," it is not clear to me how you arrived at that conclusion. Can you specifically describe the effect sizes for the above findings and how they compare to a full clinical phenotype of diastolic dysfunction? What are the thresholds for clinical and subclinical findings, and are these clearly defined in your mouse model or is this your team's expert opinion? These distinctions are important for understanding the implications of your results.
  - o Example: Lines 272-274 describe the collective differences in mice exposed to ART in terms of mtDNA content, mitochondrial function, and cardiac ultrastructure, but the magnitude of the differences (effect sizes) and their statistical significance are not clearly stated, which makes it difficult to understand the implications.

- Overall, there should be more care when discussing HIV and the critical role that ART plays in improving health outcomes. For example, in lines 92-93, the sentence should clarify that people living with HIV can have a normal expected lifespan if able to maintain an undetectable viral load by using ART throughout their life. Also, be consistent with descriptors and use non-stigmatizing language when possible. Consider using "pregnant patients living with HIV" throughout (see lines 86 and 102).

Minor Specific Comments:

- The title should clearly indicate that this study is focused on animal models without any data from human subjects.

- The first sentence of the abstract (lines 47-48) seems overly vague and somewhat misleading to me. Cardiovascular disease contributes to maternal mortality for a myriad of reasons (i.e. pre-existing cardiovascular disease, lack of access to consistent healthcare), and the cardiometabolic remodeling during pregnancy is only one of them.
  - o Similar comment for the Introduction, lines 80-81. Impaired reverse remodeling is one component of severe cardiovascular disease in pregnancy, but other factors must also be acknowledged.

- Similarly, I think the final sentence in the abstract (lines 57-58) over-states the findings. Given that this is preliminary data showing mild, subclinical cardiac phenotypes associated with NRTI-containing ART, I do not think that these findings justify clinical changes to prescribing practices for pregnant patients living with HIV. As above, it is also essential to accurately frame your findings by discussing the many well-established benefits for both maternal and fetal health of effective ART leading to viral suppression during pregnancy and postpartum.

- Line 82: The reason that there is so little understanding of mechanisms underlying the important changes in maternal physiology during pregnancy is that pregnant patients have been largely excluded from research studies, and overall there has been limited funding and support for studies during pregnancy. In light of this dearth of funding and prior research, the lack of understanding of these mechanisms is not surprising. I believe that this should be clearly stated in the Introduction.

- Lines 98-99: I recommend citing this source for the most up-to-date HIV treatment guidelines: Panel on Treatment of HIV During Pregnancy and Prevention of Perinatal Transmission. Recommendations for the Use of Antiretroviral Drugs During Pregnancy and Interventions to Reduce Perinatal HIV Transmission in the United States. Department of Health and Human Services. Available at https://clinicalinfo.hiv.gov/en/guidelines/perinatal. Accessed (insert date) [include page nubers, table number, etc., if applicable].

- Can you further explain the "vehicle control" for your study? You begin describing results in comparison to the vehicle control without describing it in the overview of your experimental design (lines 114-124).

- I found the section, "cART-induced changes in liver metabolism impact systemic lipid levels," to be well-written and the logic of your experiments was well-described. The emerging understanding of the liver-heart axis in drug-induced cardiometabolic dysfunction is an important avenue for future research.

- At the start of the Discussion section (lines 451-454) you describe the recent changes in guidelines for ART for pregnant patients living with HIV. This deserves a citation and further discussion of what the new guidelines recommend and how these medications relate to your findings.

- Need to define HFpEF (line 505) before use in the Discussion and connect this to diastolic dysfunction. This is clear to me as a clinician, but I think many readers will not understand this abbreviation and how it relates to your findings.

- There is inconsistency throughout the manuscript in terminology, i.e. the use of ART vs cART and the description of this patient population (I would suggest using "pregnant patients living with HIV," as above). Please be consistent to improve clarity.

- References 1,2, and 18 need to be reformatted as the have the title then "- PubMed." Also, there is no reference for 6, 7, or 12. There should be consistency if all the authors are listed or only the first with "et al" after. Finally, no DOIs are listed.

Alison N. Goulding, MD, MSCR
Assistant Professor, Division of Maternal-Fetal Medicine, Department of Obstetrics & Gynecology
Investigator, Behavioral Health Program, Center for Innovations in Quality, Effectiveness and Safety (IQuESt), Michael E. DeBakey VA Medical Center
Baylor College of Medicine | Texas Children's Hospital

Reviewer #4

(Remarks to the Author)
I co-reviewed this manuscript with one of the reviewers who provided the listed reports. This is part of the Nature Communications initiative to facilitate training in peer review and to provide appropriate recognition for Early Career Researchers who co-review manuscripts.

Version 1:

Reviewer comments:

Reviewer #1

(Remarks to the Author)
The revised version of the manuscript is significantly improved from an experimental perspective, as the authors have added important supporting evidence for their claims through experiments using iPSC-derived cardiomyocytes, mtDNA deep sequencing, and BN-PAGE analyses. These experiments have satsified all but one of my concerns.

While, I appreciated the quantification of mitochondria via Visiopharm (which is a reliable albeit costly, non-open source tool), the representative images provided by the authors clearly indicate to me that cristae width is increased and that mitochondria may be undergoing swelling. Indeed, the mitochondrial area they report is increased, but this seems to be only half the story. A statement regarding cristae and swelling is relevant, since this could represent a maladaptive respose (often seen in tissue damage) rather than a beneficial metabolic response. Finally, I would encourage the authors to properly label all graphs and indicate sample sizes for all main and supplemental data to improve comprehensions. Following this, I fully endorse publication from a scientific perspective.

Finally, I will take this opportunity to fully agree with the authors response to Reviewer 2's point number 2, who is totally out of line to request an HIV + ART preclinical model, even though this would be medically relevant for humans. Indeed, the

goal of the proposed manuscript is to evaluate ART treatment alone. It is relevant we understand this from a fundamental science perpspecitve before heaping on additoinal pathological insults one cannot ultimatley deconvolve. I also respectfully disagree with reviewer 3's request that "Also, be consistent with descriptors and use non-stigmatizing language when possible. Consider using “pregnant patients living with HIV” throughout (see lines 86 and 102)". I strongly encourage the authors to go back to using language that indicates referring to pregnant women and fully disagree with the changes the authors have decided to make. Honestly, I fail to see how this is stimagtizing, unless we fundamentally disagree on whether only women can become pregnant. If so, this paper is not the forum for such an assanine debate uncoupled from all logic, and would be better explored unpacked elsewhere.

Reviewer #2

(Remarks to the Author)
The authors have only been modestly responsive to my prior review. Many important factors have still not been appropriately addressed in the context of the revision and require additional attention.

Major Comments
1. In my previous review I stated the following “One significant major flaw of the present studies is that NRTIs are not examined in the context of pregnancy impacted by HIV. Although rationale is stated suggests that this was done to easily understand the impact of NTRIs on maternal physiology, pregnant women would not take NTRIs during pregnancy without HIV. In addition, without having an HIV group, it is unclear from these studies what the impact of HIV alone is on the examined parameters during pregnancy, and without knowing this it is hard to fully determine how the NRTIs are impacting these parameters. Furthermore, other rodent models have observed that HIV alone does impact many of the key processes examined.” This has not been appropriately addressed in the context of the manuscript revision, and this limitation is largely ignored in the manuscript text. Although, I understand that you wanted to examine the impact of ART on the heart independent of HIV, I am not sure of a context in which ART would be given in non-HIV patients, and because the present study design does not consider HIV alone and the combination of HIV with ART, it still remains unclear the impact of these data. It is important to decipher what phenomena stem from which scenario. Several of the examined endpoints in the present study, including mitochondrial function are known to change with HIV, so it becomes unclear what additional/additive effect ART has without first establishing a baseline phenotype of what HIV its does. At the absolute minimum these factors should be discussed in the manuscript discussion section.

2. Although a substantial amount of data is provided, there still remains limited mechanistic insight into how NRTIs impact cardiac remodeling and metabolism because many of the key findings remain superficial without in-depth analyses or complete integration. Because of this, some conclusions are made for which minimal/no data is provided, therefore, conclusions need to be further minimized unless additional supportive data is provided. Some specific examples include: Line 369 where it is stated that intracellular Na+ is likely increased giving rise to ECG changes, but Na+ is not quantified, another would be making conclusions on autophagy and mitochondrial dynamics from a single LC3 immunoblot, another would be definitively stating clear cut liver-heart cross talk based.

Minor Comments
1. The following should be articulated as a translational limitation/hurdle in the discussion: “A discussion should be provided as to why NTRIs were started at day 6 of pregnancy in these studies, when women entering pregnancy who are HIV positive may already be on a therapeutic cocktail of treatments, which could have an impact on maternal physiology if they are on these therapies for a longer pre-pregnancy duration.”
2. Some of the included immunoblots are of poor quality and need to be replaced. In addition, since no loading control or protein stain images are provided it is unclear how these blots were normalized for quantification purposes (e.g., Figure 4B and Supplemental Figure 4)
3. Line 79-80. There is also a lack of pregnant women included in clinical studies due to them being historically considered as protected subjects which likely also contributes to the lack of knowledge in this domain.
4. Please clarify the method of euthanasia used.
5. Line 624. Please indicate how much anesthesia was supplied for echocardiography.
6. Line 691. Unclear why the male line of iCells were selected for these pregnancy-related experiments over the commercially available female iCell lines from FujiFilm. Please clarify.
7. Lines 811-817. Please provide additional information regarding the AI technology used for image processing and add AI use to disclosures.
8. Lines 818-839. Please provide additional information regrading how RNA quality control strategies were used.
9. Lines 840-852. Please indicate whether the hearts used for metabolomics were freeze clamped or not. Freeze clamping is common practice for accurate examination of cardiac metabolites and by not doing so this will impact the abundance of some metabolite pools.
10. Figure 1F/G/H. Please show individual datapoints for these figures.
11. Figure 7N/O/P. Please show labeling to indicate specifically changed targets.
12. All figures please include appropriate error bars on each graph rather than solely indicating mean.
13. Figure 2. Please include representative ECG traces from examined groups.

14. The manuscript would still benefit from additional proofreading to address typographical and grammatical errors, and to reduce the use of non-standard abbreviations and correct gene-related nomenclature. In addition, the results section contains a lot of discussion, which should be moved to the discussion section to enhance clarity and accuracy of the results descriptions.
15. Some of the citations in the reference section are still not entered in the appropriate manner (e.g., Reference 1 v 2) and

some citations for unpublished, non-peer reviewed papers are included and would need to be replaced (e.g., Line 134, line 543).

Reviewer #3

(Remarks to the Author)
Overall, the authors have satisfactorily addressed all of my comments. I appreciate the time and effort they have put into addressing my comments, particularly in ensuring that their work is appropriately framed in terms of the critical role that ART plays in improving health outcomes for people living with HIV.

I have one remaining minor comment:

- Lines 494-498 (in revised manuscript): I find this phrasing confusing, and I am a clinician with expertise in the concept that I believe the authors are trying to convey. Clearer language would be helpful, i.e. " Many patients continue on alternative cART regimens during pregnancy, beyond the first-line regimens recommended in national guidelines. Some patients must change regimens in response to adverse side effects. For other patients with excellent viral suppression prior to pregnancy, expert opinion generally encourages continuation of this prior regimen even if it is not a first-line regimen recommended in pregnancy."

Reviewer #4

(Remarks to the Author)
I co-reviewed this manuscript with one of the reviewers who provided the listed reports. This is part of the Nature Communications initiative to facilitate training in peer review and to provide appropriate recognition for Early Career Researchers who co-review manuscripts.

Open Access This Peer Review File is licensed under a Creative Commons Attribution 4.0 International License, which permits use, sharing, adaptation, distribution and reproduction in any medium or format, as long as you give appropriate credit to the original author(s) and the source, provide a link to the Creative Commons license, and indicate if changes were made.
In cases where reviewers are anonymous, credit should be given to 'Anonymous Referee' and the source.
The images or other third party material in this Peer Review File are included in the article’s Creative Commons license, unless indicated otherwise in a credit line to the material. If material is not included in the article’s Creative Commons license and your intended use is not permitted by statutory regulation or exceeds the permitted use, you will need to obtain permission directly from the copyright holder.
To view a copy of this license, visit https://creativecommons.org/licenses/by/4.0/

We would like to start by thanking all reviewers for taking their time to critically review our manuscript and provide valuable feedback. The critiques were very positive and insightful, and we believe have helped strengthen the study. Below we have responded to the comments and concerns raised in a point-by-point manner. We hope the answers and experimental approaches will address the critiques and provide clarity.

Reviewer #1 (Remarks to the Author)

In the manuscript by Taube et al, the authors explore the functional impact of antiretroviral therapeutics (ARTs) on the physiological remodeling of the rat heart that manifests following pregnancy. Pregnancy is known to induce physiological hypertrophy and cardiac remodeling that is, under normal conditions, a fully reversable process. The authors propose, based on cited literature, that ARTs could negatively impact this reverse remodeling (RR) via mitochondrial DNA since ARTs have been proposed to have off-target effects on mitochondrial DNA replication. In this study, the authors perform physiological characterization of rats treated with low or high dose (LD or HD) ARTs from DG6 to PPD120 and investigate cellular and molecular remodeling using endpoint RNAseq, Metabolomics, and molecular biology and biochemistry analyses. Some mechanisms are explored in parallel in iPSC-derived human cardiomyocytes. The authors propose that ARTs modulate RR and are associated with heart failure with preserved ejection fraction (HfpEF) and mitochondrial dysfunction. While this is outside my field of expertise, it appears that a number of studies have explore the impact of ARTs (e.g. AZT) on cardiac health in rat and mouse models since the invention of ARTs, with insights into mitochondrial structure and function (PMID: 14522417, 9895221, 26248377). In my opinion, the study addresses a very important open question regarding the impact of ARTs on cardiac health in the context of pregnancy. I am unaware whether this is the first study to explore mitochondria and cardiomyopathy by ARTs during pregnancy-induced reverse remodeling in rats, and if so this study has tackled a novel question in biology and medicine.

The overall design of the study is solid, especially the detailed cardiac phenotyping in vivo and in vitro, which allowed them to observe subtle differences induced by ART using very large samples sizes. The metabolomic phenotyping is also impressive and informative and it is very important to know that the LD and HD can be quantified in situ and tracks as expected. Unfortunately, the quantitative characterization of mitochondrial function/dysfunction in this study is rather limited and primarily restricted to qPCR measurements of mtDNA levels and enzymatic measurements based on frozen material that provides limited insights into how ARTs might functionally impact mitochondrial bioenergetics at the molecular level. RNAseq was performed to monitor changes in mitochondrial gene expression and this is helpful to support the notion that mtDNA dysfunction impacts mtRNAs. However, I would caution

against the overinterpretation of transcriptional levels of metabolic and OXPHOS genes as a proxy for functional enzymatic changes, since the degree of correlation between transcriptomes, proteomes, and metabolic activities in the rodent heart is known to be variable and limited (PMID: 37276142, 36493852). Beyond these limitations, the remaining conclusions are fair and well-argued, and the data are not overinterpreted. Nevertheless, I have a number of outstanding questions and confusions raised throughout the study that require further clarification and explanation and several areas in which additional experimentation is required to substantiate the very important conclusions that were drawn in the current manuscript. I would encourage that quantitative measurements of histological and TEM changes should be provided in place of or in addition to the pathology report provided in the supplementals, which alone are insufficient to support the conclusions in the manuscript. The discussion part of the manuscript is very well-written and well-balanced.

Response: We thank the reviewer for the positive feedback. In response to one of the points raised above, we would like to clarify that, to the best of our knowledge, this is the only study publicly available that evaluated the effects of cART in a pre-clinical model of peripartum exposure using combined therapy (rather than single agents) as currently recommended and provided for patients living with HIV. It is also, to our knowledge, the first study to dissect the cardiovascular effects of the combined regimen in the absence of the HIV virus, addressing a critical gap of knowledge in the field. Although individuals living with HIV are at increased risk of developing cardiovascular disease, the relative contributions of chronic infection, cART, or their interaction remain largely unknown.

Major concerns

1. The major substrate for energy metabolism in the adult heart is fatty acids, and it is important to assess respiration using fatty acids and not carbohydrate substrates, particularly given the intriguing fatty acid measurements reported in Figure 5. Substrate preference changes during cardiac hypertrophy and its metabolic inflexibility in this regard could underscore defects in reverse remodeling. The authors acknowledge this important possibility in lines 483-485 of the discussion but do not directly test it.

Response: The reviewer raises an excellent point. We invested considerable effort in attempting to adapt the Seahorse assay using previously frozen samples to assess lipid-supported respiration by supplementing lysates with electron donors involved in β-oxidation, following principles similar to those described in the original protocol developed by the Shirihai group (PMID: 32432379). Unfortunately, none of the strategies we tested yielded reliable or interpretable measurements. To address the reviewer's question regarding substrate utilization, we performed complementary experiments using the human iPSC-derived cardiomyocytes exposed to the same cART for 7 days, followed by substrate

flexibility testing using the Seahorse flux analyzer. All data were normalized to protein content. Because this exposure reproduced the ECG results obtained in the animals (Fig. 2P-S), we reasoned that this approach would provide useful insights. We found that although both control and cART-treated cells were capable of respiring on multiple substrates, including fatty acids, amino acids and glucose, cART-treated cells exhibited diminished capacity and flexibility in oxidizing glucose (**new Fig. 4C**). Interestingly, a study recently showed that cardiac mitochondria increase their metabolic flexibility to support remodeling during pregnancy, and postpartum cardiac mitochondria respire more efficiently on carbohydrates and amino acids than on other substrates (PMID: 3855148). Given the accumulation of glycogen observed in the hearts of treated animals by EM, and the established role of glycogen mobilization in providing glucose to sustain cardiac function under stress (PMID: 24080183), these data collectively suggest that impaired metabolic flexibility and glucose utilization may contribute to cART-induced defects in post-partum cardiac reverse remodeling. This information has been added to **Page 15**.

2. Of course, altered lipid profiles could reflect modified bioenergetic substrate usage (as the authors propose) or lipotoxicity leading to myocyte death. Neither are mutually exclusive and both are functionally different and important. Neither has been functionally evaluated and both together, or individually, could contribute impaired reverse remodeling. Measurement of TUNEL staining of cardiac histology or serum cardiac troponin levels can be used to provide insights into myocyte death.

Response: This is an important point. Although not included in the initial submission, we measured serum levels of multiple cardiac injury biomarkers, including several troponin isoforms and natriuretic peptides, using ELISA. In contrast to the positive controls supplied with the assays, all biomarkers were below the detection limit (pmol range) in both control and treated animals. While the absence of these biomarkers in control samples was expected, the lack of detectable signal in treated animals suggests that their levels are unlikely to be meaningfully elevated relative to controls. We acknowledge that absence of detection does not preclude subtle changes; however, multiple independent lines of evidence provided in the manuscript argue against cardiac injury or cardiomyocyte death at a level sufficient to explain the reported phenotypes. First, RNA-seq analysis revealed no transcriptional changes between treated and control animals in troponin isoforms, natriuretic peptides, or dystrophin, genes commonly associated with cardiac damage (Supplementary Table 2). Second, H&E histological analysis did not reveal evidence of cardiomyocyte death in the treated samples, which would be expected to produce early and detectable morphological abnormalities. Third and most importantly, cardiomyocyte loss is typically associated with impaired cardiac function, including reduced ejection fraction and cardiac output (PMID: 14638549). In contrast, the echocardiographic measurements (Fig. 1)

provided in the manuscript demonstrate increased cardiac function, including ejection fraction, stroke volume, fractional shortening, and cardiac output, during pregnancy and the postpartum period. Although the sustained increase in function several months postpartum is abnormal, it is not consistent with cardiomyocyte death that would – conversely – lead to functional loss. Collectively, these findings indicate that cardiomyocyte death is unlikely to meaningfully, or at all, contribute to the cardiac effects reported.

3. I am highly skeptical of the ability to conclude on mitochondrial respiration (rather than specific enzymatic activities of OXPHOS) using frozen rather than fresh biopsies. While enzymatic assays of I-III-IV and II-III-IV have been performed on frozen material for decades, these assess the specific activities of enzymes and not mitochondrial respiration. I am aware that protocols (cited by the authors) have been published on this topic, but the limits of this system do not allow one to assess the coupling of mitochondria, which is critical for cardiac function in vivo. In the absence of pregnancy, ARTs have been proposed to trigger mitochondrial toxicity through various mechanisms that can have direct (cristae) or indirect impacts on respiration and coupling therefore, making such studies highly relevant for functional insights into reverse remodeling that is (according to the authors fig Supplementary 3D), independent of mitochondrial content.

Response: We appreciate the reviewer's skepticism and agree that fresh and frozen tissues are not identical for OCR measurements. However, the group that established this assay performed extensive validation, including direct 1:1 comparison between fresh and frozen samples, demonstrating preservation of relative respiratory measurements (PMID: 32432379). Because freezing disrupts membrane integrity and abolishes mitochondrial coupling and ADP control, OCR measurements with frozen samples measure ETC enzymatic capacities rather than integrated, coupled respiration, which we have now made clear by revising the text on **page 13**. In an attempt to gather more information, we used two complementary approaches. First, we performed blue-native gels which revealed no changes in complex I or II protein content relative to vehicle control at either time point (**new Fig. 4B).** Second**,** and in response to point 1 above, we established fuel dependency and flexibility of iPS-derived cardiomyocytes treated with cART, which showed decreased capacity of glucose utilization (**new Fig. 4C).** As postpartum cardiac mitochondria were shown to preferentially utilize glucose and amino acids, rather than lipids, for bionenergetics (PMID: 3855148), these data indicate that the changes in ETC enzymatic activities identified in the tissue were reflective of broader functional changes caused by cART that are needed to properly support cardiac RR.

4. *Given the literature and proposed mechanisms of ARTs on mtDNA (including reference 16 provided by the authors), I would have expected mtDNA mutations and deletions to*

accompany the depletion of mtDNA. It would be valuable if the authors could use alternative approaches to explore mtDNA deletions and mutational load triggered by long-term ART because it is difficult to imagine that the modest depletion of mtDNA content alone reported in Figure 3 has any meaningful impact on respiratory chain activities reported in the study. A clear indication of the fold changes should be reported in the manuscript.

Response: This is an important point that we too were curious about. To gather more information, we performed next-generation sequencing of mtDNA isolated from cardiac tissue. Due to limited material availability, we analyzed n=5 animals/group at each time point and employed a PCR-based pre-sequencing amplification approach, as previously described (PMID: 32036083). These **new data**, now included in **Fig. 3F–J** and **Supplementary Data 3B–C**, demonstrate an increase in mutation frequency associated with drug exposure that appears to accumulate over time. Specifically, at PPD120, we observed significant increases in the rate of short deletions (<200 bp) (Fig. 3H) and in relative deletion frequency (Fig. 3I), along with a borderline significant increase in deletion rates when rearrangements were included (Fig. 3J). Consistent with these findings, treated animals also exhibited increased heteroplasmy (Supplementary Data 3B). No significant differences were detected in base substitutions (transitions or transversions) or insertions (Supplementary Data 3C). Together, these results indicate that cART not only alters mtDNA content, as previously reported, but also increases mtDNA mutation frequency. The combined impact of these changes likely contributes to the mitochondrial and cardiac effects observed in the treated animals. This information has been included on **Page 13**.

5. Defects in the integrity or amount of mtDNA would be expected to quantitatively impact that amount of functional OXPHOS complexes, particularly since the authors report changes in CI, CII, and IV activities (Figures 4F, 6R). The only quantitative measurements I could find were of SDHA and TOM20 (by SDS-PAGE?), which showed no differences, but examining one structural subunit out of the >100 proteins of the OXPHOS system is hardly sufficient to exclude effects on OXPHOS assembly/maintenance. Hence, it remains to be demonstrated how or even if (see major concern point 2 above) OXPHOS differences are differentially regulated. Proteomic profiling of these hearts to measure the steady state levels of mitochondrial proteins or BN-PAGE using cardiac mitochondria would provide meaningful insights into this phenomenon.

Response: We thank the reviewer for the thoughtful comment and suggestion. As indicated in the response to point 3 above, we have performed blue-native PAGE (BN-PAGE) to determine potential changes in steady state levels of mitochondrial ETC proteins that might play a role in the phenotypes observed. Because of limited material left, we opted for BN-PAGE probing subunits of both complexes I and II, the same enzymatic activities assayed

using the Seahorse Flux Analyzer thereby allowing direct comparisons of the data. We reasoned that changes in those would be significantly more informative given the enzymatic output of these complexes probed previously. BN-PAGE confirmed that protein abundance of the ETC components was not different between the samples (**new Fig. 4B**). In addition, we performed Western blot analyses for the autophagy/mitophagy marker LC3-II, a key mediator of damaged mitochondrial removal and recycling in the heart (PMID: 28546358). We observed no significant induction of organelle turnover in treated animals (new Supplementary Data 4B). These findings are consistent with our RNA-seq results, which did not reveal significant changes in the expression of genes involved in mitophagy in the heart, including *Atg5, Bnip3, Lamp2, Pink1, Parkin, Fbxo32, Mfn1,* and *Mfn2* (PMID: 28546358; Supplementary Table 2). Collectively, these results support the conclusion that mitochondrial protein content and complex assembly are not significantly altered under our experimental conditions.

6. The same care for detailed quantification of in vivo cardiac function and cardiomyocyte function should be applied to the histology and imaging data in a way that is easily visualized in the main or supplementary figures, notably for Figure S3D, given the defects of mitochondrial structure that have been documented in HIV+ and/or hypertrophic hearts. There is a wealth of fascinating and relevant observations in the pathology report that are not quantified in the supplemental data, which limits their appreciation and their value in the manuscript's current form.

Response: We thank the reviewer for this thoughtful comment. In collaboration with our pathology core facility and a computational biologist, we employed an artificial intelligence (AI)–based approach to quantify mitochondrial area in the images. To validate the accuracy of the AI-based analysis, we compared its output with manual quantification of mitochondrial perimeter performed on samples from PPD21. Although perimeter and area are distinct metrics, both can reflect potential changes in mitochondrial size. Accordingly, data from both methods demonstrated high concordance at PPD21, which we judged sufficient to attest to the suitability of the method for quantifying data from samples at PPD120.The **new quantitative data** are provided in **Supplementary Data 3E and F**. Our analysis focused on mitochondria because they were the principal structures affected by cART exposure. More importantly, extending the AI-based analysis to other subcellular structures requires substantial additional time to train and validate new models, which must include manual benchmarking to ensure comparability. While we think this is a valid point, we also believe that specific characterization of all cardiac structural changes associated with cART exposure is beyond the scope of the current revision. We hope the reviewer will agree.

Minor concerns.

1. *The authors' use of LD and HD ARTs reveals physiological differences that might be attributed to different degrees of cardiac and mitochondrial toxicity. According to the authors, the LD is already 2.4x the abacavir and lamivudine prescribed to humans, while the dolutegravir is 10x the amount. For the HD, these values are 4.8x and 20x respectively (lines 118 to 120). What is the rationale of using such artificially high doses (even the LD, which incurs limited effects throughout the study) if the aim of the study is to provide mechanistic insights into the pathophysiological relevance of ARTs for human health? Is there a biological basis for the use of these elevated doses in rats, perhaps based different susceptibilities and pharmacokinetics between species? If so, it must be more clearly explained and the correct references used for the selection of these doses must be provided.*

Response: We appreciate the reviewer's important question regarding dose selection and its relevance to human health, which has made us realize that the doses of dolutegravir and lamivudine were wrongly reported in the text and in Fig. 1A, while correctly reported in Supplementary Data 1 and in the methods section. The correct data now show that all drugs are present as ~2.5 or ~5X higher relative to humans (see calculations below), for the LD and HD, respectively.

**Doses of HIV drugs utilized in the heart studies and their animal to human conversion**							
human triumeq		human/mg/kg		rat studies/mg/kg	HED mg/kg		HED mg/kg
600 mg of abacavir		10		150	24.19354839	300	48.38709677
50 mg of dolutegravi		0.8		12.5	2.016129032	25	4.032258065
300 mg of lamivudin		5		75	12.09677419	150	24.19354839
					**low dose**		**high dose**
				rat dose utilized compared to human dosage	**2.419354839**		**4.838709677**
					**2.52016129**		**5.040322581**
					**2.419354839**		**4.838709677**

These doses were selected based on data derived from toxicokinetics (TK) as well as dose-range finding (DRF) studies that took place prior to the reported experiments. These data are currently being prepared for submission (Beames et al., in preparation). TK and DRF studies are routinely performed by us to define drug plasma concentrations and doses that may show signs of toxicity, respectively, prior to a study. In the case of cART specifically, our experience indicates that they generally exhibit faster clearance, lower bioavailability, and reduced tissue exposure compared with humans, necessitating higher mg/kg doses to achieve systemic and tissue drug levels that approximate those observed clinically. The TK and DRF studies that we performed counted with 4 different cART concentrations, ranging from 50/6.12/37.5mg/Kg up to 600/50/300mg/kg. Accordingly, both the LD and HD regimens selected produced plasma exposures within, or modestly above, the therapeutic range observed in humans, rather than supraphysiological levels. Importantly, the LD group,

despite producing minimal overt phenotypic effects, served to establish a threshold for biological sensitivity, while the HD group was designed to unmask mechanistic pathways that may be engaged under conditions of increased stress. This approach is commonly employed in toxicological studies to identify subclinical or early cellular alterations that may not be readily detectable when using only low exposure levels. Finally, it is important to point that the drugs are delivered to patients living with HIV at a fixed dose, which is provided to any patient of 100lbs or above. Thus, a 12-year-old child, a pregnant woman and a 300 lbs adult receive the exact same dosage daily, which nevertheless has different concentration on a weight basis. We agree that this rationale should be more explicitly stated. We have therefore revised the manuscript to correct and clarify the biological basis for dose selection, which can be found on **page 6.**

2. *The authors propose that HD induces molecular and functional changes not observed with LD. Indeed, the severity of the cardiac dysfunction is more substantial in HD versus LD rats in Figures 2A, B, C, D, E, F, G, H, and I. However, in some functional tests, the LD procures more dysfunction than the HD relative to vehicle control, as in Figures 2K and 1H, as well as in Supplementary Data 2B and 2D. These observations seem at odds and necessitate explanation.*

Response: We thank the reviewer for carefully examining the functional data and for highlighting this important point. We agree that while HD treatment generally produces more pronounced cardiac dysfunction across most functional parameters, a subset of measures shows greater impairment in the LD group relative to HD when compared with vehicle controls. We believe these observations are not contradictory but instead reflect dose-dependent differences in the nature of the cardiac response. Specifically, HD exposure appears to induce a more uniform and sustained phenotype characterized by global changes in cardiac metabolism, structure and function, whereas LD exposure produces subtler, more variable alterations that may differentially affect specific functional endpoints. In this context, certain parameters, particularly those that are highly sensitive to loading conditions, heart rate variability, or compensatory remodeling, may exhibit non-linear or biphasic dose responses. Such non-monotonic effects are well documented in toxicology (PMID: 1460028), including in cardiovascular physiology and mitochondrial stress responses, where low-level perturbations can transiently exaggerate dysfunction prior to the engagement of adaptive or compensatory mechanisms that may (partially) normalize function at higher doses. Consistent with this interpretation, HD animals show coordinated molecular, mitochondrial, and structural changes that align with the predominant functional phenotype, whereas LD animals lack many of these convergent molecular signatures yet still display isolated functional deviations. This has been clarified in this revision (**Page 9**).

3. Figure legends need further explanations, particularly Figures 3F, 6R, 4E-I (statistically significant differences must be identified). Statistical tests and significant differences (or lack thereof) must be indicated. In some heat maps, I have the impression that all genes/metabolites are shown while in others, it is only the statistically significantly dysregulated ones. This should be more clearly defined in the figure legends. Figure 6N-P gene labels are missing.

Response: We apologize for the oversight regarding the figure legends, which have now been corrected. We note that gene labels were not included in Figure 6N–P due to the large number of features, which would have compromised readability. However, this information is provided separately in Supplementary Table 3 (now Supplementary Table 4 following the addition of a new table). To clarify the point raised regarding the genes and metabolites displayed in the heatmaps, all features shown represent statistically significant differences in at least one experimental group - now stated in the figure legend. This approach allows visualization of how each gene or metabolite behaves across all groups and time points.

4. References used to support the notion that ARTs drive mitochondrial DNA depletion (references 9, 12, and 16) should be reconsidered and revised or replaced to better substantiate their claims. For example, reference 9 is a review that references two studies (ref 17 and 19), neither of which directly support the claim with experimental evidence relevant to the study

• PMID: 35631648 – this study examined the impact of ARTs on placenta mtDNA, which was not depleted but rather modestly elevated in some conditions. Difficult to conclude since embryonic development was also negatively impacted.

• PMID: 25635584 is itself a review

• Reference 12 is incomplete and I was therefore unable to source the study

• Reference 16 is a much more thorough review – what I gather is that misincorporation nucleotides is more frequent or perhaps more frequently explored than mtDNA depletion, which begs the question about mtDNA mutational load (see major concerns point 3 above)

Response: As per the request, we have provided additional references and removed the ones that were not relevant.

5. On line 251-260, the authors state that the "half-life of cardiac mitochondria is estabimated to be ~20 days" and hence by PPD21 "~75% of the intial population of mitochondria would have turned over, by PPD120 all that content would have been replaced". This arugment is based on Reference 19 – which is a review with little experimental data to suggest proposed turnover rate and is too simple a view. In fact the 70% figure comes

from a referenced paper – (PMID: 20364102) from this review in which HL-1 cells were starved for 3.5h and then mito content was assessed by fluorescence. A more accurate representation of cardiac mitochondrial protein turnover rates in the rodent heart in vivo was performed using heavy water labeling (PMID: 22915825, 26977904) under healthy and diseased conditions, revealing that half-lives of mitochondrial proteins vary dramatically even for proteins belonging to the same macromolecular complex and can be much more or much less than 20days. It remains possible and untested whether mitochondrial protein content is altered in this study.

Response: We agree with the reviewer that these estimates may not be fully accurate. Accordingly, we have revised the manuscript to remove the paragraph in question (**Page 12**), while retaining the data in Supplementary Data 4A. Importantly, we have obtained **new data** using BN-PAGE indicating that complex I subunits remain unchanged (r**evised Fig. 4B**). In addition, we performed Western blot analyses for the autophagy/mitophagy marker LC3-II, a key mediator of damaged mitochondrial removal and recycling in non-dividing cells (PMID: 28546358). We observed no significant induction of organelle turnover in treated animals (new Supplementary Data 4B). These findings are consistent with our RNA-seq results, which did not reveal significant changes in the expression of genes involved in mitophagy in the heart, including *Atg5, Bnip3, Lamp2, Pink1, Parkin, Fbxo32, Mfn1,* and *Mfn2* (PMID: 28546358; Supplementary Table 2). Collectively, these results support the conclusion that mitochondrial protein content and complex assembly are not significantly altered under our experimental conditions to an extent that would influence the observed phenotypes.

6. *In Figure 6, it is surprising that ALP and ALT levels revealed no obvious liver injury, although liver metabolism and lipids were dysregulated. It is possible that initial liver injury (e.g. PPD20) was present but compensated through hepatocyte proliferation by PPD120. If ALP and ALT levels cannot be measured at early time points, supporting the absence of livery injury at PPD120 by normal histology would strengthen this claim.*

Response: We thank the reviewer for this comment and note that no signs of liver injury were identified through gross pathological evaluation as well as histopathological staining of the of the at PPD21 and at PPD120. It is important to note that ALP and ALT are relatively insensitive markers of subtle or chronic metabolic liver stress - often remaining within the normal range in the absence of overt hepatocellular injury or necrosis even in the context of liver diseases such as NAFDL or MASH (PMID: 23536736, PMID: 40243565, PMID: 39352015).

7. *Given the impact of ART on body weight, it may be more appropriate to quantify the ration of heart weight/tibia length rather than heart weight/body weight. However, in light of the extensive in vivo echo and ECG studies, this index has relatively little value.*

Response: We agree with the reviewer that normalization of heart weight to tibia length is a more appropriate metric than normalization to body weight. However, when the studies were designed, the tibia was not collected precluding us from using it for heart weight quantification.

8. I wondered whether the arrhythmia triggers HFpEF or are the defects in cardiac structure causing arrhythmia?

Response: This is an excellent and valid question, which we unfortunately cannot provide an unequivocal answer as our studies were not designed to directly address the temporal or causal relationship between arrhythmia and HFpEF. To the best of our knowledge, available evidence supports a bidirectional relationship between these phenotypes that is influenced by multiple factors, many of which were not systematically assessed in this study. Nonetheless, this is a valuable point for consideration for future studies.

9. There appears to be an error on line 694 (frozen placental lysate).

Response: This error has been fixed.

10. *The authors indicate that statistical outliners were identified (line 763) but were these then excluded? If so, on which basis and in which data sets?*

Response: Outlier identification was performed using GraphPad Prism. Specifically, the ROUT (Robust regression followed by Outlier Test) method was applied to each dataset. This approach first fits a robust regression model that minimizes the influence of potential outliers. Residuals, defined as the differences between the observed values and the robust fit, are then analyzed to identify data points that deviated significantly from the model and thus are labeled as outliers. Outliers were detected using a false discovery rate (FDR)-based approach. Data points identified as outliers were excluded from subsequent analyses and were not part of data presented in the figures.

11. Error on line 791 for reference 12 –

Response: we appreciate the identification of the error that we had not noticed; it has now been corrected.

Reviewer #2 (Remarks to the Author)

The goal of this study was to examine the impact of a combination therapy of three nucleotide reverse transcriptase inhibitors (NRTIs) on pregnancy-induced changes in cardiac remodeling in the rat. The study shows that NRTIs contributed to impaired pregnancy-associated remodeling and reversal and changes in mitochondrial biology in the hearts and livers of the dam's receiving treatment. Although there is a lot of data provided, significant

issues were noted which must be addressed. These are noted as major and minor issues below.

Major Comments

1. One significant major flaw of the present studies is that NRTIs are not examined in the context of pregnancy impacted by HIV. Although rationale is stated suggests that this was done to easily understand the impact of NTRIs on maternal physiology, pregnant women would not take NTRIs during pregnancy without HIV. In addition, without having an HIV group, it is unclear from these studies what the impact of HIV alone is on the examined parameters during pregnancy, and without knowing this it is hard to fully determine how the NRTIs are impacting these parameters. Furthermore, other rodent models have observed that HIV alone does impact many of the key processes examined.

Response: We appreciate the reviewer's concern. As stated in the manuscript, people living with HIV have an elevated risk for cardiovascular disease (CVD), yet it remains unclear whether this risk is driven by persistent low-level viremia, by ART itself, or by a combination of both (PMID: 31154814). Thus, the goal of this study was to dissect the contribution of ART – in the absence of the virus - to maternal cardiac adaptations during pregnancy. This represents a critical gap of knowledge in the field. To accomplish this, we used the combination of three antiretrovirals: two NRTIs and one integrase inhibitor that, until June 2025, constituted the recommended regimen for women during and after pregnancy. By examining drug exposure in the absence of HIV or viral proteins, we were able to isolate the physiological and molecular effects of the medications themselves. Our findings reveal two novel observations: (1) this ART combination significantly alters the cardiometabolic health of pregnant dams, and (2) tissue-level analyses indicate that these cardiac effects are driven primarily by the NRTIs, not by the integrase inhibitor.

The reviewer notes that "rodent models have observed that HIV alone does impact many of the key processes examined." However, it is important to clarify that there is no true rodent model of full HIV infection, as rodents cannot be infected with the human virus. The most widely used system is the HIV-1 transgenic (Tg) rat (Reid et al., 2001), which carries a gag-pol-deleted provirus expressing only three viral proteins: Gp120, Tat, and Nef. These proteins have been documented in immune cells, as well as in serum and cerebrospinal fluid, making the model useful for studying HIV-Associated Neurocognitive Disorder (HAND). However, there is minimal evidence that these viral proteins exert direct effects on the heart. In fact, a recent yet-to-be-published study (conference abstract) reported detection of viral proteins only in immune-cell–like populations within the heart, and not in cardiomyocytes. In contrast, the drug-induced effects observed in our study, transcriptional, metabolic, and functional, predominantly reflect changes within cardiomyocytes themselves. Given this,

the HIV-1 Tg rat is unlikely to provide a meaningful or mechanistically appropriate model to compare to the outcomes we examined. Lastly, it is worth highlighting that exposure to ART is not limited to individuals living with HIV. Pre-exposure prophylaxis (PrEP) is recommended by U.S. federal agencies for individuals at elevated risk of HIV acquisition, including HIV-negative women with HIV-positive partners, explicitly to keep them HIV-negative. Thus, ART exposure during pregnancy can occur even in the absence of HIV infection.

We hope these points help to clarify the rationale and the significance of our study design.

2. Although a substantial amount of data is provided, there is limited mechanistic insight into how NRTIs impact cardiac remodeling and metabolism and many of the key observations remain superficial without in-depth analyses or complete integration, such as the interrogations into the examination of reverse remodeling, diastolic dysfunction, mitochondrial dynamics, and interorgan crosstalk. Because of this, some conclusions are made for which no data is provided, such as the stated differences in substrate utilization and the modest integration of the heart and liver omics datasets to comment on organ cross talk. In addition, changes in glycogen metabolism are stated; however, if TEM samples are not rapidly collected, glycogen can be released and deposited quickly, which reflect an artefact of sample collection rather than a biological change, so glycogen metabolism should be examined further should these conclusions be made.

Response: We appreciate the reviewer's critique. However, we would like to clarify that the primary objective of this study was not to define precise mechanistic pathways, which are not readily feasible in a rat model given limitations on, for example, genetic tools Nevertheless, rats provide a more physiologically relevant model of human cardiac function than mice, and this consideration was essential for addressing the questions posed in this study. In response to the reviewer's comments on mechanisms, fuel utilization and glycogen, we have performed additional experiments in iPSC-derived cardiomyocytes exposed to the same cART for 7 days, followed by substrate flexibility testing using the Seahorse flux analyzer. All data were normalized to protein content. Because this exposure reproduced the ECG results obtained in the animals (Fig. 2P-S), we reasoned that this approach would provide relevant mechanistic insights. We found that, although both control and cART-treated cells were capable of respiring on multiple substrates, including fatty acids, amino acids and glucose, cART-treated cells exhibited increased capability of using fatty acids but decreased utilization of glucose, with therefore diminished flexibility in utilizing carbohydrates as a carbon source (**new Fig. 4C**). Interestingly, a recent study investigating cardiac mitochondrial metabolism during pregnancy and the postpartum period showed that these organelles increase metabolic flexibility to support cardiac remodeling during pregnancy. In the postpartum state, specifically, cardiac mitochondria

respire more efficiently on carbohydrates and amino acids than on other substrates (PMID: 3855148). Given the accumulation of glycogen observed in the hearts of treated animals by TEM, together with the established role of glycogen mobilization in providing glucose to sustain cardiac function under stress (PMID: 24080183), these findings collectively suggest that impaired metabolic flexibility in glucose utilization may contribute to cART-induced defects in postpartum cardiac reverse remodeling. This information has been added (**Page 15**). Lastly, we note that changes in glycogen were supported by TEM, by transcriptomic and by metabolomic analyses (Supplementary Tables 2 and 3). Furthermore, since TEM samples were processed identically, and glycogen accumulation was observed exclusively in the HD group, it is unlikely that a processing artifact occurred only in those. While we do not claim this represents a definitive mechanism underlying the observed effects, these findings highlight an area for further investigation.

Minor Comments

1. *Line 453 seems to suggest that the treatment regimen tested in the context of these studies is no longer than the ideal treatment regimen for pregnant women with HIV. This should be discussed further because without additional discussion the clinical importance of these studies becomes unclear. In addition, a discussion should be provided as to why NTRIs were started at day 6 of pregnancy in these studies, when women entering pregnancy who are HIV positive may already be on a therapeutic cocktail of treatments, which could have an impact on maternal physiology if they are on these therapies for a longer pre-pregnancy duration.*

Response: While the specific ART combination used in our study is no longer the preferred guideline recommendation (Biktarvy is now preferred) it remains a clinically relevant regimen. Current recommendations emphasize that pregnant patients living with HIV should remain on their existing ART throughout pregnancy whenever possible. Thus, this combination continues to be used when patients conceive while already stabilized on it, and the guideline-recommended preferred regimen is primarily implemented when complications arise or when no prior treatment is in place. We also note, as emphasized by our clinical collaborator at Thomas Jefferson University, Dr. Dagan Coppock, that clinicians currently lack a robust scientific basis to guide their selection among available regimens. Dr. Coppock has specifically highlighted the value of this work in providing concrete biological evidence that may help clinicians make more informed decisions about whether to continue, or reconsider, the combination investigated here.

We agree with the reviewer that, in clinical practice, women living with HIV would often begin cART before becoming pregnant. However, for this study, drug administration began on gestational day 6 for two key reasons. First, precise timing of pregnancy was essential across

all cohorts to ensure comparability, and detecting pregnancy in rats is considerably more challenging than in mice. Second, the cost of the antiretrovirals (approximately $300,000 for this study) necessitated beginning exposure at a time point that allowed rigorous experimental control without incurring prohibitive expenses. We are currently discussing a follow up study in which animals would be exposed prior to, during and after pregnancy to both define the effects of pre-pregnancy exposure but also to better recapitulate real life exposures. As per the request of the reviewer, we have added information on **Page 26** about GD6.

2. Please include additional information regarding the litters in this study, such as sex distribution and birth weights. It also remains unclear whether the treatment regimens impact on the offspring because minimal interrogation has been performed, which should be discussed.

Response: Additional information including litter sex distribution and birth weights among other parameters have been added in the supplementary data as requested. We note, however, that the health effects identified in the pups that were indirectly exposed are the subject of a separate manuscript that is currently under preparation.

3. There is a reference in line 490 that is not yet published and should be removed from this; however, given the direct relevance to the present studies, it is unclear why this data is not included here.

Response: The referenced work has since been submitted. That study involves a different experimental design incorporating co-exposures, in which the cART-only cohort represents one of several groups. Although it provides relevant information on the effects of the same drugs on cardiac function in non-pregnant animals, the data are not directly comparable and therefore cannot be included in the present manuscript.

4. The title should be changed to accurately match the key observations in the study. At present, based on the data provided, it is unclear whether HFpEF is truly observed.

Response: As per the request, we have removed HFpEF from the title.

5. There are a handful of inaccurate statements made regarding the timeline of the reversal of pregnancy-associated remodeling and CVD being the leading cause of maternal mortality that needs to be revised.

Response: Revised as requested.

6. The manuscript would benefit from additional proofreading to address typographical and grammatical errors, and to reduce the use of non-standard abbreviations and correct gene-related nomenclature. In addition, some of the citations in the reference section are not

entered into in the appropriate manner and some of the included figures need to include additional labeling so that it is clear what timepoint is being examined.

Response: We have thoroughly revised the paper and hope to have addressed all points raised.

Reviewer #3 (Remarks to the Author):

The authors used multiple approaches to rigorously investigate the effects of antiretroviral treatment (ART) containing nucleoside reverse transcriptase inhibitors (NRTIs) on maternal cardiac function and reverse remodeling in the postpartum period. Given the significant maternal morbidity and mortality related to cardiovascular disease, particularly in pregnant patients living with HIV, and the long-term impacts on cardiovascular (CV) health, this work is timely and significant. I do have several questions and comments about this manuscript, as detailed below. My main concern relates to the authors' framing of the study, particularly the lack of acknowledgment that viremic control through ART (with NRTIs as a core component of most modern ART regimens) is critical in order to reduce risk of vertical transmission to the child and to promote long-term wellbeing of the mother. Based on current evidence, these reductions in harm markedly outweigh the cardiometabolic risks associated with ART, as described in the national guidelines that dictate clinical practice in the United States (Panel on Treatment of HIV During Pregnancy and Prevention of Perinatal Transmission. Recommendations for the Use of Antiretroviral Drugs During Pregnancy and Interventions to Reduce Perinatal HIV Transmission in the United States. Department of Health and Human Services. Available at https://clinicalinfo.hiv.gov/en/guidelines/perinatal). While I realize that the authors may not be clinicians, when examining risks of NRTI-containing ART, it is essential to understand the tremendous benefits of these medications and not to present the risks in isolation. I think that both the Introduction and the Discussion need to be re-framed to reflect this so that these results and their implications can be properly interpreted.

Major Specific Comments:

- The Introduction discusses CV risks during pregnancy and the postpartum period as well as CV risks with long-term antiretroviral use. However, it proposes investigating both the process of reverse remodeling as well as the impact of ART on reverse remodeling. A more thorough discussion about what is currently known about the impact of antiretrovirals on cardiac health could be beneficial here (or in the discussion), and then connected back to how ART can further impact cardiovascular health during the critical period of pregnancy and

postpartum. Also, some explanation of the impact of lactation on CVD and how that informs the authors' decision to analyze cardiometabolic health after weaning would be helpful to frame the study design.

Response: To our knowledge, existing data on the effects of ART in pregnant women living with HIV have largely focused on fetal or offspring outcomes. Cardiovascular data in this population are therefore limited; however, relevant evidence exists in the broader HIV-positive population. To address the reviewer's comment without detracting from the primary focus of our study, we have revised text on the known cardiovascular effects of ART in people living with HIV (**pages 4,16 and 22**), citing the most recent review articles on the topic. We hope this addition adequately addresses the reviewer's concern.

- Justification of the selection of the specific ART regimen and dosing studied here, in addition to the time points for assessments and the relationship to lactation (as above), would be helpful towards the beginning of the manuscript. As a clinician who prescribes ART to my pregnant patients, I was confused about the choice of ART regimen as it is not commonly used in current practice.

Response: This was also a point raised by reviewer 1. We have revised the text on **page 6** to comprehensively address this comment.

- More specifics in the Results sections in general would be helpful. The authors tend to describe general trends and then reference figures, without providing numerical results to help convey the effect size and the statistical significance of the findings. In light of this, I did not fully appreciate until the end of the Discussion section that the observed cardiac phenotypes were mild and subclinical. This is an important point and it was not immediately clear from the Results as currently presented.

o Example: While line 207 describes the collective findings as indicating of "subclinical features of diastolic dysfunction," it is not clear to me how you arrived at that conclusion. Can you specifically describe the effect sizes for the above findings and how they compare to a full clinical phenotype of diastolic dysfunction? What are the thresholds for clinical and subclinical findings, and are these clearly defined in your mouse model or is this your team's expert opinion? These distinctions are important for understanding the implications of your results. o Example: Lines 272-274 describe the collective differences in mice exposed to ART in terms of mtDNA content, mitochondrial function, and cardiac ultrastructure, but the magnitude of the differences (effect sizes) and their statistical significance are not clearly stated, which makes it difficult to understand the implications.

Response: We thank the reviewer for this comment and agree that the presentation of effect sizes and clinical relevance could be clearer. GraphPad Prism, the software used for

statistical analysis in this study, provides effect size estimates as part of its output. However, while some of the results reflect a statistically robust effect in terms of variance explained (for example the effect on cardiac output on Fig. 2A), from a physiological perspective this corresponds to ~10–12% change. Such a difference in cardiac output is modest when compared with established rodent models of overt cardiac dysfunction, where substantially larger changes (>40%) are typically observed (PMID: 32710530; PMID: 40818383). Most changes detected in this study were in the ~10% range, with some exceptions. Therefore, while effect size provides important statistical context, we believe that relying on effect size alone as a metric of biological or clinical significance may be misleading in this setting, particularly given the potential implications of these findings for a broad population that remains reliant on cART.

Lastly, all significant p-values are shown on the figure, and the statistical threshold presented in the figure legend, as per journal guidelines.

- Overall, there should be more care when discussing HIV and the critical role that ART plays in improving health outcomes. For example, in lines 92-93, the sentence should clarify that people living with HIV can have a normal expected lifespan if able to maintain an undetectable viral load by using ART throughout their life. Also, be consistent with descriptors and use non-stigmatizing language when possible. Consider using "pregnant patients living with HIV" throughout (see lines 86 and 102).

Response: We thank the reviewer for this important feedback. We have revised the manuscript to more carefully and accurately reflect the critical role of antiretroviral therapy in improving health outcomes for people living with HIV. (**Page 5 and Page 22**). In addition, we have reviewed the manuscript for consistency and have updated terminology to use person-first, non-stigmatizing language throughout (e.g., "pregnant patients living with HIV"), including at the locations noted by the reviewer. These changes were made throughout to ensure clarity, accuracy, and respectful framing.

Minor Specific Comments:

- The title should clearly indicate that this study is focused on animal models without any data from human subjects.

Response: The title has been revised as requested.

- The first sentence of the abstract (lines 47-48) seems overly vague and somewhat misleading to me. Cardiovascular disease contributes to maternal mortality for a myriad of reasons (i.e. pre-existing cardiovascular disease, lack of access to consistent healthcare), and the cardiometabolic remodeling during pregnancy is only one of them.
o Similar comment for the Introduction, lines 80-81. Impaired reverse remodeling is one

component of severe cardiovascular disease in pregnancy, but other factors must also be acknowledged.

- Similarly, I think the final sentence in the abstract (lines 57-58) over-states the findings. Given that this is preliminary data showing mild, subclinical cardiac phenotypes associated with NRTI-containing ART, I do not think that these findings justify clinical changes to prescribing practices for pregnant patients living with HIV. As above, it is also essential to accurately frame your findings by discussing the many well-established benefits for both maternal and fetal health of effective ART leading to viral suppression during pregnancy and postpartum.

Response: We thank the reviewer for pointing this out; we have revised the abstract to reflect the points raised while maintaining the word count required by the journal.

- Line 82: The reason that there is so little understanding of mechanisms underlying the important changes in maternal physiology during pregnancy is that pregnant patients have been largely excluded from research studies, and overall there has been limited funding and support for studies during pregnancy. In light of this dearth of funding and prior research, the lack of understanding of these mechanisms is not surprising. I believe that this should be clearly stated in the Introduction.

Response: We thank the reviewer for this comment and have included this in the revised introduction (see **page 4**).

- Lines 98-99: I recommend citing this source for the most up-to-date HIV treatment guidelines: Panel on Treatment of HIV During Pregnancy and Prevention of Perinatal Transmission. Recommendations for the Use of Antiretroviral Drugs During Pregnancy and Interventions to Reduce Perinatal HIV Transmission in the United States. Department of Health and Human Services. Available at https://clinicalinfo.hiv.gov/en/guidelines/perinatal. Accessed (insert date) [include page nubers, table number, etc., if applicable].

- Can you further explain the "vehicle control" for your study? You begin describing results in comparison to the vehicle control without describing it in the overview of your experimental design (lines 114-124).

Response: We have included this resource page and better explained the vehicle control as requested. These are presented on page 6 where information on dose and drug selection, as per reviewer 1, have been included.

- I found the section, "cART-induced changes in liver metabolism impact systemic lipid levels," to be well-written and the logic of your experiments was well-described. The

emerging understanding of the liver-heart axis in drug-induced cardiometabolic dysfunction is an important avenue for future research.

Response: We appreciate the positive comment and agree that this is an important area that needs more in-depth studies and understanding. In fact, we are actively developing a complex in vitro system incorporating placental, hepatic, intestinal, and cardiac cells to investigate underlying mechanisms, including the role of inter-organ crosstalk. These models will allow for more direct mechanistic testing with detailed understanding of molecular targets and tissue interdependencies.

- At the start of the Discussion section (lines 451-454) you describe the recent changes in guidelines for ART for pregnant patients living with HIV. This deserves a citation and further discussion of what the new guidelines recommend and how these medications relate to your findings.

Response: We agree that this is important and have revised the first paragraph of the discussion accordingly (see revised **page 22**).

- Need to define HFpEF (line 505) before use in the Discussion and connect this to diastolic dysfunction. This is clear to me as a clinician, but I think many readers will not understand this abbreviation and how it relates to your findings.

- There is inconsistency throughout the manuscript in terminology, i.e. the use of ART vs cART and the description of this patient population (I would suggest using "pregnant patients living with HIV," as above). Please be consistent to improve clarity.

Response: Changes have been made throughout for consistency and clarity as requested. Regarding ART vs cART, the former refers to single therapy while cART reflects the use of *combined* antiretrovirals, which - by definition - includes more than one drug type.

- References 1,2, and 18 need to be reformatted as the have the title then "- PubMed." Also, there is no reference for 6, 7, or 12. There should be consistency if all the authors are listed or only the first with "et al" after. Finally, no DOIs are listed.

Response: We thank the reviewer for pointing these out, which have been fixed in the revised manuscript.

We appreciate the positive feedback and minor requests from Reviewers 1 and 3, and thank them for their careful evaluation of the manuscript. We also thank Reviewer 2 for the constructive feedback. All remaining issues raised by the reviewers have now been addressed.

REVIEWER COMMENTS

Reviewer #1 (Remarks to the Author):

The revised version of the manuscript is significantly improved from an experimental perspective, as the authors have added important supporting evidence for their claims through experiments using iPSC-derived cardiomyocytes, mtDNA deep sequencing, and BN-PAGE analyses. These experiments have satsified all but one of my concerns.

While, I appreciated the quantification of mitochondria via Visiopharm (which is a reliable albeit costly, non-open source tool), the representative images provided by the authors clearly indicate to me that cristae width is increased and that mitochondria may be undergoing swelling. Indeed, the mitochondrial area they report is increased, but this seems to be only half the story. A statement regarding cristae and swelling is relevant, since this could represent a maladaptive respose (often seen in tissue damage) rather than a beneficial metabolic response. Finally, I would encourage the authors to properly label all graphs and indicate sample sizes for all main and supplemental data to improve comprehensions. Following this, I fully endorse publication from a scientific perspective.

Finally, I will take this opportunity to fully agree with the authors response to Reviewer 2's point number 2, who is totally out of line to request an HIV + ART preclinical model, even though this would be medically relevant for humans. Indeed, the goal of the proposed manuscript is to evaluate ART treatment alone. It is relevant we understand this from a fundamental science perpspecitve before heaping on additoinal pathological insults one cannot ultimatley deconvolve. I also respectfully disagree with reviewer 3's request that "Also, be consistent with descriptors and use non-stigmatizing language when possible. Consider using “pregnant patients living with HIV” throughout (see lines 86 and 102)". I strongly encourage the authors to go back to using language that indicates referring to pregnant women and fully disagree with the changes the authors have decided to make. Honestly, I fail to see how this is stimagtizing, unless we fundamentally disagree on whether only women can become pregnant. If so, this paper is not the forum for such an assanine debate uncoupled from all logic, and would be better explored unpacked elsewhere.

Response to Reviewer #1:

We thank the thoughtful evaluation of the revised manuscript and for recognizing the additional experimental work performed. We appreciate the reviewer’s important

observation regarding mitochondrial morphology. In response, we have revised the manuscript to acknowledge that the increased mitochondrial area observed in our analyses may reflect not only mitochondrial remodeling, but also potential mitochondrial swelling and altered cristae architecture that can be associated with maladaptive responses in the context of cellular stress or tissue injury. This information has now been added to the revised text (Lines 317-319, p. 15).

Reviewer #2 (Remarks to the Author):

The authors have only been modestly responsive to my prior review. Many important factors have still not been appropriately addressed in the context of the revision and require additional attention.

Major Comments

1. In my previous review I stated the following “One significant major flaw of the present studies is that NRTIs are not examined in the context of pregnancy impacted by HIV. Although rationale is stated suggests that this was done to easily understand the impact of NTRIs on maternal physiology, pregnant women would not take NTRIs during pregnancy without HIV. In addition, without having an HIV group, it is unclear from these studies what the impact of HIV alone is on the examined parameters during pregnancy, and without knowing this it is hard to fully determine how the NRTIs are impacting these parameters. Furthermore, other rodent models have observed that HIV alone does impact many of the key processes examined.” This has not been appropriately addressed in the context of the manuscript revision, and this limitation is largely ignored in the manuscript text. Although, I understand that you wanted to examine the impact of ART on the heart independent of HIV, I am not sure of a context in which ART would be given in non-HIV patients, and because the present study design does not consider HIV alone and the combination of HIV with ART, it still remains unclear the impact of these data. It is important to decipher what phenomena stem from which scenario. Several of the examined endpoints in the present study, including mitochondrial function are known to change with HIV, so it becomes unclear what additional/ additive effect ART has without first establishing a baseline phenotype of what HIV its does. At the absolute minimum these factors should be discussed in the manuscript discussion section.

2. Although a substantial amount of data is provided, there still remains limited

mechanistic insight into how NRTIs impact cardiac remodeling and metabolism because many of the key findings remain superficial without in-depth analyses or complete integration. Because of this, some conclusions are made for which minimal/no data is provided, therefore, conclusions need to be further minimized unless additional supportive data is provided. Some specific examples include: Line 369 where it is stated that intracellular Na+ is likely increased giving rise to ECG changes, but Na+ is not quantified, another would be making conclusions on autophagy and mitochondrial dynamics from a single LC3 immunoblot, another would be definitively stating clear cut liver-heart cross talk based.

Minor Comments

1. The following should be articulated as a translational limitation/hurdle in the discussion: “A discussion should be provided as to why NTRIs were started at day 6 of pregnancy in these studies, when women entering pregnancy who are HIV positive may already be on a therapeutic cocktail of treatments, which could have an impact on maternal physiology if they are on these therapies for a longer pre-pregnancy duration.”
2. Some of the included immunoblots are of poor quality and need to be replaced. In addition, since no loading control or protein stain images are provided it is unclear how these blots were normalized for quantification purposes (e.g., Figure 4B and Supplemental Figure 4)
3. Line 79-80. There is also a lack of pregnant women included in clinical studies due to them being historically considered as protected subjects which likely also contributes to the lack of knowledge in this domain.
4. Please clarify the method of euthanasia used.
5. Line 624. Please indicate how much anesthesia was supplied for echocardiography.
6. Line 691. Unclear why the male line of iCells were selected for these pregnancy-related experiments over the commercially available female iCell lines from FujiFilm. Please clarify.
7. Lines 811-817. Please provide additional information regarding the AI technology used for image processing and add AI use to disclosures.
8. Lines 818-839. Please provide additional information regrading how RNA quality control strategies were used.
9. Lines 840-852. Please indicate whether the hearts used for metabolomics were freeze clamped or not. Freeze clamping is common practice for accurate examination of cardiac metabolites and by not doing so this will impact the abundance of some metabolite pools.
10. Figure 1F/G/H. Please show individual datapoints for these figures.
11. Figure 7N/O/P. Please show labeling to indicate specifically changed targets.
12. All figures please include appropriate error bars on each graph rather than solely indicating mean.

13. Figure 2. Please include representative ECG traces from examined groups.
14. The manuscript would still benefit from additional proofreading to address typographical and grammatical errors, and to reduce the use of non-standard abbreviations and correct gene-related nomenclature. In addition, the results section contains a lot of discussion, which should be moved to the discussion section to enhance clarity and accuracy of the results descriptions.
15. Some of the citations in the reference section are still not entered in the appropriate manner (e.g., Reference 1 v 2) and some citations for unpublished, non-peer reviewed papers are included and would need to be replaced (e.g., Line 134, line 543).

Response to Reviewer #2:

We thank the reviewer for the additional comments, but note that careful evaluation of the revised manuscript would have clarified issues that remain – as noted below - as they were addressed in the first round of revision. We also note that comments, like the assumption that we used male iPSC to model pregnancy, were surprising and incorrect. As per editorial decision, no additional in vivo experiments were carried out.

Major Comment 1

We agree that understanding the independent and combined effects of HIV infection and antiretroviral exposure during pregnancy represents an important translational question. Once again, we remind the reviewer that the primary objective of the present study, however, was to isolate the maternal cardiovascular and metabolic effects of NRTI-containing cART exposure only during pregnancy **independent of viral effects**, given that both HIV infection and antiretroviral therapy can independently influence mitochondrial function, cardiac remodeling, and systemic metabolism. Dissecting drug-specific effects required an experimental design that minimized confounding effects from viral pathology. We also reiterate that **antiretroviral exposure in the absence of HIV infection is clinically relevant in the context of pre-exposure prophylaxis (PrEP). As per CDC guidelines, women at high risk of contracting HIV are recommended to take PrEP ART** during conception and pregnancy despite not being infected with HIV.

While the absence of an HIV-only comparator represents a translational limitation if only considering that drugs are taken by people carrying the virus, which is not the case, adding a model in which the expression of ectopic viral proteins that do not reach the heart is not the appropriate approach. It is feasible to speculate that the presence of those proteins at the systemic level may lead to cardiovascular outcomes, but those experiments remain beyond the scope of the manuscript. We have revised the discussion to include this point, and the importance of future studies to address this question (Lines 573–574).

Major Comment 2

Once again, we would respectfully remind the reviewer that the primary goal of this study was to establish whether maternal cART exposure induces cardiac, metabolic, and mitochondrial alterations in pregnancy using complementary vivo and cellular approaches. It was not our goal to fully define the molecular mechanisms underlying each phenotype. We have softened the statements in the areas highlighted by the reviewer.

Minor Comments

1. Gestational day 6 treatment initiation

Information regarding this was included in the previous revision in the Materials and Methods. It is now also mentioned in the results section (Lines 126-128, 609-613).

2. Immunoblot quality and normalization

Representative total protein/loading control images have been added to the revised supplementary materials. Methods have also been updated with appropriate antibody information (Lines 765-769).

3. Clinical studies in pregnancy

While this is a valid point, having pregnant women protected in clinical trials raises ethical concerns beyond the scope of this study.

4. Method of euthanasia

Information regarding this was included in the previous revision in the Materials and Methods. It has been highlighted again for the reviewer.

5. Echocardiography anesthesia

Information regarding this was included in the previous revision in the Materials and Methods. It has been highlighted again for the reviewer.

6. Male iCell line selection

It is unclear where the reviewer defined that the cells used in this study are from male origin. We carefully selected the cells for this study specifically because they are from a female donor – see information below.

Components:

Donor Background

**Cell Type:** Cardiomyocytes	**Sex:** Female
**Donor Status:** Apparently Healthy Normal	**Modeled Disease:** Apparently Healthy Normal
**Race:** Caucasian	**Tissue Source:** Fibroblast
**Age At Sampling:** <18	**Engineered Line:** No

7. AI-assisted image processing

Additional details regarding the AI-assisted image processing workflow have now been provided in the Methods, and AI use has been added to the disclosures (Line 831).

8. RNA quality control

Additional RNA quality control procedures and inclusion criteria have now been described in the Methods (Line 836).

9. Freeze clamping for metabolomics

We thank the reviewer for this important methodological point. Hearts were not freeze clamped. While we understand that freeze clamping, particularly after excision and perfusion by Langendorff, is ideal, all hearts were processed the same way and therefore should mitigate any differences due to collection method.

10. Figure 1F/G/H

Individual data points have now been added.

11. Figure 7N/O/P

Labels have now been added to all heat maps.

12. Error bars

Error bars have now been added to all relevant graphs.

13. Representative ECG traces

Representative ECG traces will be provided in open-source data. We refer the reviewer there.

14. Proofreading and manuscript organization

The manuscript has undergone additional proofreading to correct typographical and grammatical errors, standardize nomenclature, and reduce non-standard abbreviations.

15. References

The reference list has been carefully reviewed and reformatted for consistency. Non-peer reviewed literature will be updated as it undergoes peer review.

Reviewer #3 (Remarks to the Author):

Overall, the authors have satisfactorily addressed all of my comments. I appreciate the time and effort they have put into addressing my comments, particularly in ensuring that their work is appropriately framed in terms of the critical role that ART plays in improving health outcomes for people living with HIV.

I have one remaining minor comment:

- Lines 494-498 (in revised manuscript): I find this phrasing confusing, and I am a clinician with expertise in the concept that I believe the authors are trying to convey. Clearer language would be helpful, i.e. " Many patients continue on alternative cART regimens during pregnancy, beyond the first-line regimens recommended in national guidelines. Some patients must change regimens in response to adverse side effects. For other patients with excellent viral suppression prior to pregnancy, expert opinion generally encourages continuation of this prior regimen even if it is not a first-line regimen recommended in pregnancy."

Response to Reviewer #3:

We thank the reviewer for this helpful feedback and for your thoughtful review of our revisions. We appreciate your recognition of our efforts to appropriately frame the importance of ART in improving outcomes for people living with HIV. We agree that the phrasing in lines 494–498 could be clearer. In response to your suggestion, we have revised this section accordingly (Lines 497-502, p. 22-23).

Reviewer #4 (Remarks to the Author):

I co-reviewed this manuscript with one of the reviewers who provided the listed reports. This is part of the Nature Communications initiative to facilitate training in peer review and to provide appropriate recognition for Early Career Researchers who co-review manuscripts.
